# Coordination Chemistry of Thorium Supported by a Single Polyoxoalkoxide Ligand

**DOI:** 10.1021/acs.inorgchem.6c03105

**Published:** 2026-09-12

**Authors:** Dominic Shiels, Adriana C. Berlfein, William W. Brennessel, Ellen M. Matson

**Affiliations:** Department of Chemistry, 6927 University of Rochester, Rochester, New York 14627, United States

## Abstract

Actinide-polyoxometalate (An-POM) literature is dominated by the formation of 1:2 An­(POM)_2_ sandwich-type complexes. Here, we report selective formation of a 1:1 An-POM complex using non-aqueous methods. Treatment of the lacunary POM precursor, (TBA)_2_[Mo_5_O_13_(OMe)_4_NO]­[Na­(MeOH)], or the 1:2 Th-POM sandwich-type complex, (TBA)_2_[Th­{Mo_5_O_13_(OMe)_4_NO}_2_], with ThCl_4_·2DME (DME = dimethoxyethane) in dimethylformamide leads to selective formation of the 1:1 Th-POM complex, (TBA)­[ThCl_2_(DMF)_2_Mo_5_O_13_(OMe)_4_NO]. (TBA)­[ThCl_2_(DMF)_2_Mo_5_O_13_(OMe)_4_NO] represents a rare example of an An-POM complex which possesses multiple labile ligands (DMF/Cl^–^) in its inner coordination sphere, providing an opportunity to study fundamental coordination chemistry processes. We report initial investigations probing neutral ligand exchange, halide abstraction, and salt metathesis at the thorium center supported by a POM, isolating a series of mixed-ligand species spanning a range of Th coordination geometries. This work establishes a versatile route to the controlled modification of actinide coordination environments in An-POM chemistry.

## Graphical abstract

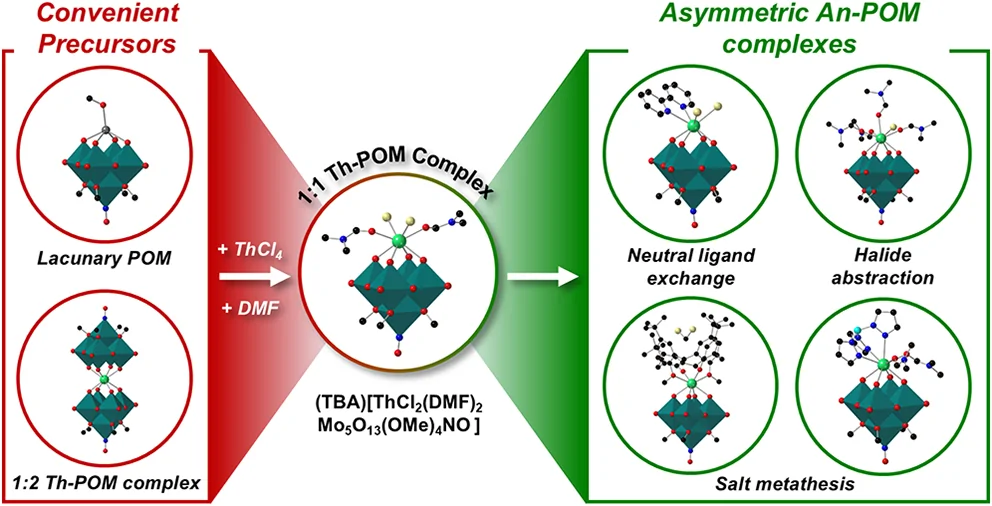

## Introduction

The coordination chemistry of actinides is an active area of research, with a primary goal being to develop an understanding of how the access to relatively low energy, and radially extended, valence 5f orbitals influences electronic structure and bonding.^1–3^ This has potential implications in separations chemistry,^4–9^ small molecule activation,^3,10–15^ and catalysis,^16–19^ where 5f orbital participation in bonding can give rise to unique properties of compounds and materials. Historically, studies have probed the fundamental coordination preferences of actinides with multidentate organic ligands.^20–25^ In recent years, polyoxometalates (POMs), which are multi-metallic molecular metal oxides typically constructed from W^VI^, Mo^VI^, or V^V^ ions, have become an increasingly popular platform for studying the coordination chemistry of actinides.^26–40^ A driving factor behind this trend is that the high molecular weights of POMs (1,000–20,000 g mol^–1^) limit the quantities of radioactive material required for complex formation on measurable scales. Furthermore, POMs are chemically (and radiolytically) robust, oxidatively stable, and readily manipulated in the presence of air/water.

A survey of the literature reveals that An-POM chemistry is dominated by two broad structural classes. In one class, the actinide is encapsulated within a POM framework, as observed in Silverton-type [AnMo_12_O_42_]^x–^ and Preyssler-type [AnP_5_W_30_O_110_]^x–^ assemblies.^39,41–45^ In the other class of complexes, the actinide is sandwiched between two lacunary POMs (e.g., Keggin-type [EW_11_O_39_]^X–^, where E = B, Si, P, Ge, Ga, or Lindqvist-type [W_5_O_18_]^6–^) to form An­(POM)_2_ complexes.^26–37^ A consistent feature of both broad structural classes, and other An-POM structures that are not easily classified,^46–50^ is that the coordination sphere of the actinide is typically saturated, with no (or few) vacant coordination sites available for binding of substrates or additional ligands. Consequently, An-POM chemistry has remained focused largely on structural and spectroscopic characterization, while studies examining fundamental coordination chemistry and reactivity at the actinide center remain rare.

To carry out reactivity studies of actinide centers supported by POM metalloligands, one would first have to prevent the formation of An­(POM)_2_ sandwich-type complexes to free up sites for ligand and/or substrate binding. Interestingly, a handful of aqueous studies examining the solution speciation of either An- or lanthanide-POM complexes using NMR, UV-Vis/NIR and/or luminescence spectroscopy support the formation of a mixture of 1:2 An­(POM)_2_ and 1:1 (H_2_O)_*x*_An­(POM) complexes in solution, with the exact nature of the mixture depending on factors including the identity of the POM precursor and solution pH.^32,33,51–54^ These works provide evidence for the existence of 1:1 An-POM complexes in which the actinide coordination sphere contains numerous water ligands (Figure 1). While in theory these solvent molecules might be readily displaced by other ligands/substrates, the dynamic nature of these aqueous solutions has prevented isolation of 1:1 An-POM complexes to date.

**Figure 1:**
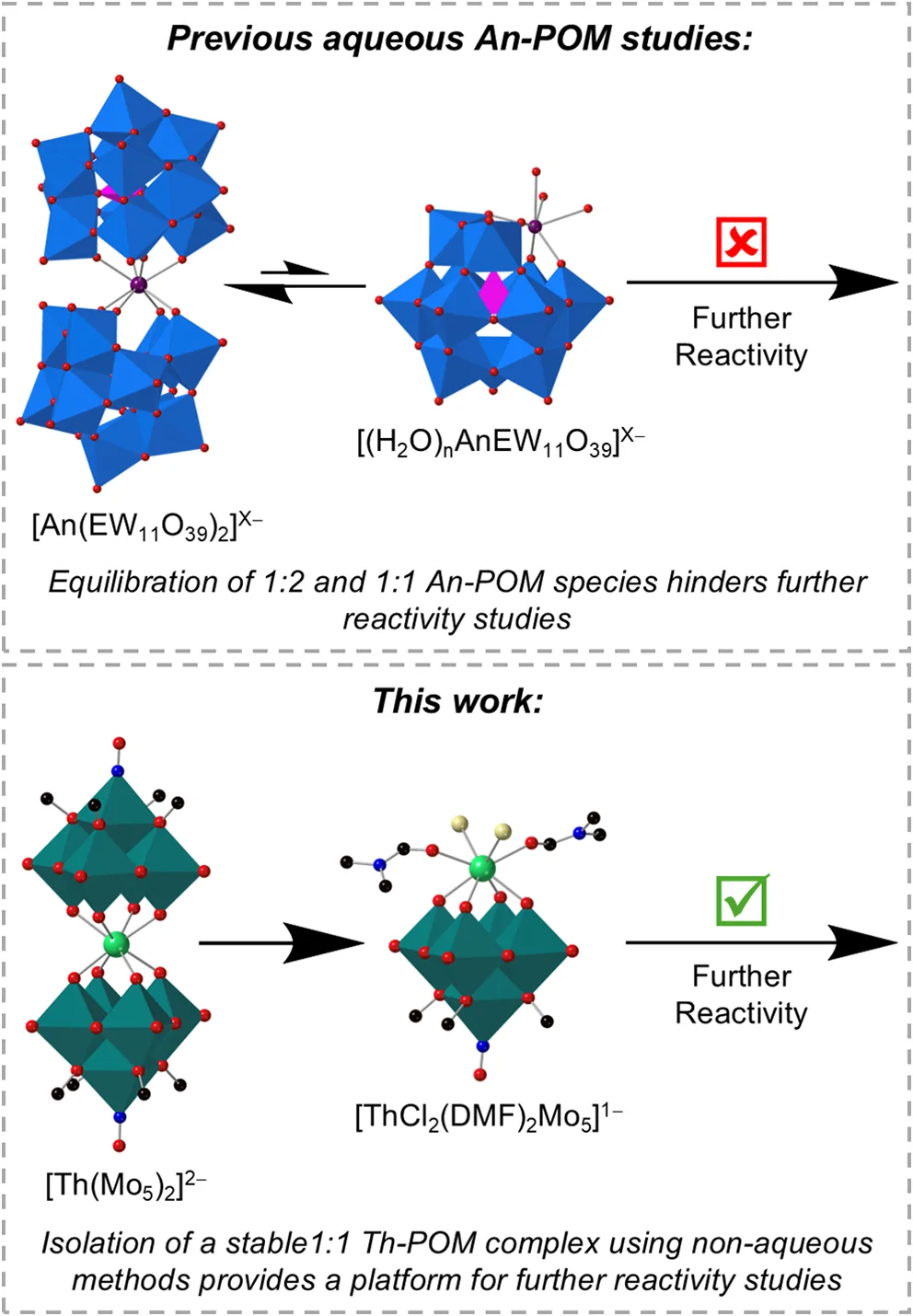
Overview of the dynamic nature of classic An-POM species, which readily equilibrate between 1:2 and 1:1 species (top), compared to the 1:1 Th-POM complex studied in this work which is stable with respect to the formation of the 1:2 complex (bottom).

Our group has been investigating the formation of a series of An­(POM)_2_ complexes using non-aqueous methods. Treatment of (TBA)_2_[M_4_O_13_(OMe)_4_MoNO]­[Na­(MeOH)] (M = Mo or W) with AnCl_4_·xDME salts (An = Th, Np; *x* = 2, An = U; *x* = 0; DME = dimethoxyethane) in methanol (MeOH) results in selective formation of (TBA)_2_[An^IV^{M_4_O_13_(OMe)_4_MoNO}_2_] sandwich-type complexes.^55–57^ ^1^H NMR studies performed on the uranium-centered derivatives in a range of solvents (including dichloromethane, acetone, acetonitrile, and dimethyl sulfoxide) show that in all cases only the sandwich-type species (i.e., the 1:2 complex) exists in solution.^56^ This lack of dynamic behavior led us to hypothesize that if we could form the corresponding 1:1 An-POM complex in organic solvent, it might be less likely to equilibrate with the 1:2 complex. This in turn could act as a starting point for studies focused on examining the coordination chemistry of actinide centers supported by a single POM.

Herein, we demonstrate that the 1:1 Th-POM complex (TBA)­[ThCl_2_(DMF)_2_Mo_5_O_13_(OMe)_4_NO] can be prepared by treatment of either (TBA)_3_[Mo_5_O_13_(OMe)_4_NO]­[Na­(MeOH)] (**1-NaMo**_**5**_) or (TBA)_2_[Th­{Mo_5_O_13_(OMe)_4_NO}_2_] (**2-Th­(Mo**_**5**_ **)**_**2**_) with one equivalent of ThCl_4_•2DME in DMF (Figure 1, bottom). The presence of the strongly coordinating solvent appears to be crucial in stabilizing the 1:1 Th-POM complex, with previous reactions performed in solvents like MeOH or THF failing to yield the target species.^56^ Once formed, (TBA)­[ThCl_2_(DMF)_2_Mo_5_O_13_(OMe)_4_NO] resists equilibration with **2-Th­(Mo**_**5**_ **)**_**2**_ or exchange of the DMF ligands with common solvents such as MeCN or THF. The stable 1:1 Th-POM complex provides a platform for further reactivity studies at the Th center, with neutral ligand exchange, chloride abstraction and salt metathesis reactions all reported here. These studies provide convenient access to asymmetric sandwich-type complexes in which Th is stabilized by two different multidentate ligands, expanding the range of accessible coordination geometries for actinide centers supported by a POM ligand.

## Experimental Section

### General Considerations

Air- and moisture-sensitive manipulations with all other complexes were carried out using a standard high-vacuum line, Schlenk techniques, or an MBraun inert atmosphere drybox containing an atmosphere of purified dinitrogen. The MBraun glovebox was equipped with a cold well designed for freezing samples in liquid nitrogen, as well as a −35 °C freezer for cooling samples and crystallizations. Solvents for sensitive manipulations were dried and deoxygenated using literature procedures with a Seca solvent purification system or a glass contour solvent purification system (Pure Process Technology, LLC) and stored over activated 4 Å molecular sieves (Fisher Scientific) prior to use. Deuterated solvents were purchased from Cambridge Isotope Laboratories, dried with molecular sieves and degassed by three freeze–pump–thaw cycles. (TBA)_4_[Mo_8_O_26_],^58^ **1-NaMo**_**5**_,^59^ ThCl_4_·2DME,^60^ **2-Th­(Mo**_**5**_ **)**_**2**_,^56^ and ^tBu^CalixMe_2_Na_2_ were synthesized using previously reported methods.^61^ ^17^O enriched **1-NaMo**_**5**_ and **2-Th­(Mo**_**5**_ **)**_**2**_ were synthesized using approaches similar to those used to acquire non-enriched materials, as previously described.^57^

### Safety Considerations

^232^Th is a weak α-emitter (4.082 MeV) with a half-life of 1.41 × 10^10^ years. Manipulations and reactions should be carried out in monitored fume hoods or in an inert atmosphere drybox in a radiation laboratory equipped with α and β counting equipment.

### Synthesis of (TBA)­[ThCl_2_(DMF)_2_Mo_5_O_13_(OMe)_4_NO] (3-ThCl_2_(DMF)_2_Mo_5_)

#### Method A

In a 20 mL scintillation vial, (TBA)_2_[Mo_5_O_13_(OMe)_4_NO]­[Na­(MeOH)] (0.5 g, 0.36 mmol, 1 equiv) was dissolved in DMF (2 mL) forming a pink-purple solution. This solution was then added to a separate 20 mL scintillation vial containing solid ThCl_4_·2DME (0.2 g, 0.36 mmol, 1 equiv) with stirring. A dark blue, slightly cloudy, solution formed. The mixture was stirred for 10 min before adding to a vial containing vigorously stirred Et_2_O (ca. 15 mL). This led to the precipitation of a light purple powder. The powder was allowed to settle before the solvent was discarded. The powder was washed with Et_2_O (2 × 15 mL) to ensure complete removal of free DMF. The solid was dissolved in MeCN (ca. 5 mL) and then filtered through a bed of Celite (approx. 1 cm). The solution was dried under vacuum leaving a slightly tacky solid. The solid was triturated with Et_2_O (2 × 5 mL) and then dried under vacuum leaving a free-flowing pale purple powder (0.34 g, 62% yield). Blue-purple crystals were obtained by vapor diffusion of Et_2_O into a saturated solution of the product in MeCN. ^**1**^H NMR (500 MHz, CD_2_Cl_2_) δ 1.04 (t, *J* = 7.4 Hz, 12H), 1.49 (h, *J* = 7.7 Hz, 8H), 1.70 (m, 8H), 3.06 (s, 6H), 3.09 (s, 6H), 3.23 (m, 8H), 4.73 (s, 12H), 8.12 (s, 2H). ^17^O enriched (TBA)­[ThCl_2_(DMF)_2_Mo_5_O_13_(OMe)_4_NO] was obtained by following the procedure described above with ^17^O enriched (TBA)_2_[Mo_5_O_13_(OMe)_4_NO]­[Na­(MeOH)]. ^17^O NMR (67.8 MHz, CD_2_Cl_2_) δ 914.9 (4 O), 717.3 (4 O), 519.2 (4 O), 14.5 (1 O). UV-Vis/NIR (21 ^o^C, DCM): *λ*_max_ (*ε*) = 568 nm (53 M^–1^ cm^–1^). Anal. calcd for C_26_H_62_N_4_O_20_Cl_2_Mo_5_Th: C, 20.36%; H, 4.08%; N, 3.65%. Found: C, 20.33%; H, 4.10%; N, 3.66%.

#### Method B

In a 20 mL scintillation vial, (TBA)_2_[Th­{Mo_5_O_13_(OMe)_4_NO}_2_] (0.81 g, 0.34 mmol, 1 equiv) was dissolved in DMF (1 mL) forming a purple solution. ThCl_4_·2DME (0.19 g, 0.34 mmol, 1 equiv) was dissolved in DMF (1 mL) and added to the solution of (TBA)_2_[Th­{Mo_5_O_13_(OMe)_4_NO}_2_]. The mixture immediately turned dark blue. The mixture was stirred for 10 min before adding to a vial containing vigorously stirred Et_2_O (15 mL). This led to the precipitation of a light purple powder. The powder was allowed to settle before the solvent was discarded. The powder was then washed with Et_2_O (2 × 15 mL) to ensure complete removal of free DMF. The solid was dried under vacuum to give crude product (0.89 g, 86%) as a pale purple powder. Blue-purple crystals were obtained by vapor diffusion of Et_2_O into a saturated solution of the product in MeCN. ^17^O enriched (TBA)­[ThCl_2_(DMF)_2_Mo_5_O_13_(OMe)_4_NO] was obtained by following the procedure described above with ^17^O enriched (TBA)_2_[Th­{Mo_5_O_13_(OMe)_4_NO}_2_]. The obtained characterization data matched that given above using method A.

### Synthesis of (TBA)­[ThCl_2_(bipy)­Mo_5_O_13_(OMe)_4_NO] (4-ThCl_2_(bipy)­Mo_5_)

In a 20 mL scintillation vial, (TBA)­[ThCl_2_(DMF)_2_Mo_5_O_13_(OMe)_4_NO] (0.050 g, 0.032 mmol, 1 equiv) was dissolved in MeCN (1 mL) forming a dark blue solution. This solution was added to a vial containing solid 2,2’-bipyridine (0.010 g, 0.064 mmol, 2 equiv). The mixture was stirred for 5 min before adding to a vial containing vigorously stirred Et_2_O (15 mL). This led to the precipitation of a light grey/green powder. The powder was allowed to settle before the solvent was discarded. The powder was then washed with Et_2_O (2 × 10 mL) to ensure complete removal of DMF and free bipy. The solid was dried under vacuum to remove residual solvent, yielding the crude product (0.030 g, 60%) as a grey/green powder. Green-blue crystals were obtained by vapor diffusion of Et_2_O into a saturated solution of the product in DCM. ^1^H NMR (500 MHz, CD_3_CN) δ 0.94 (t, *J* = 7.4 Hz, 12H), 1.33 (m, *J* = 7.5 Hz, 8H), 1.62–1.52 (m, 8H), 3.05 (t, *J* = 8.7 Hz, 8H), 4.67 (s, 12H), 7.71 (t, *J* = 5.9 Hz, 2H), 8.16 (t, *J* = 7.6 Hz, 2H), 8.32 (d, *J* = 8.0 Hz, 2H), 9.26 (s, 2H). ^17^O enriched (TBA)­[ThCl_2_(bipy)­Mo_5_O_13_(OMe)_4_NO] was obtained by following the procedure described above with ^17^O enriched (TBA)­[ThCl_2_(DMF)_2_Mo_5_O_13_(OMe)_4_NO]. ^17^O NMR (67.8 MHz, CD_3_CN) δ 13.5 (1 O), 527.7 (4 O), 711.6 (4 O), 922.5 (4 O). UV-Vis/NIR (21 ^o^C, DCM): λ_max_ (ε) = 568 nm (67 M^–1^ cm^–1^). Anal. calcd for C_30_H_56_N_4_O_18_Cl_2_Mo_5_Th·DCM: C, 22.87%; H 3.59%; N, 3.44%. Found: C, 22.62%; H, 3.39%; N, 3.61%.

### Synthesis of (TBA)­[CalixThMo_5_O_13_(OMe)_4_NO] (5-CalixThMo_5_)

In a 20 mL scintillation vial, (TBA)­[ThCl_2_(DMF)_2_Mo_5_O_13_(OMe)_4_NO] (0.1 g, 0.065 mmol, 1 equiv) was dissolved in THF (7 mL) forming a blue solution. This solution was added to a separate 20 mL scintillation vial containing solid CalixNa_2_ (0.047 g, 0.065 mmol, 1 equiv). The mixture was stirred for 30 min during which time the mixture turned golden yellow and the majority of the solid dissolved, leaving a slightly cloudy solution. The mixture was filtered through a bed of Celite (approx. 1 cm) before the solvent was removed. The obtained tan solid was washed with Et_2_O (2 × 5 mL) to remove free DMF, before being dissolved in DCM (5 mL). The solution was filtered through a bed of Celite (approx. 1 cm) before drying under vacuum to give the crude product as a tan powder (0.102 g, 79% yield). Golden-brown crystals were obtained by vapor diffusion of Et_2_O into a saturated solution of the product in DCM. ^**1**^H NMR (500 MHz, CD_2_Cl_2_) δ 0.99 (s, 18H), 1.03 (t, *J* = 7.4 Hz, 12H), 1.24 (s, 18H), 1.46 (m, 8H), 1.58 (m, 8H), 3.06 (m, 8H), 3.17 (d, *J* = 12.7 Hz, 4H), 4.07 (s, 6H), 4.37 (d, *J* = 12.3 Hz, 4H), 4.79 (s, 12H), 6.81 (s, 4H), 7.03 (s, 4H). ^17^O enriched (TBA)­[CalixThMo_5_O_13_(OMe)_4_NO] was obtained by following the procedure described above with ^17^O enriched (TBA)­[ThCl_2_(DMF)_2_Mo_5_O_13_(OMe)_4_NO]. ^17^O NMR (67.8 MHz, CD_2_Cl_2_) δ 13.8 (1 O), 501.4 (2 O), 513.8 (2 O), 722.5 (4 O), 909.2 (4 O). UV-Vis/NIR (21 ^o^C, DCM): λ_max_ (ε) = 558 nm (69 M^–1^ cm^–1^) + more intense absorption that onsets below 550 nm. Anal. calcd for C_66_H_108_N_2_O_22_Mo_5_Th·0.5DCM: C, 39.23%; H, 5.40%; N, 1.38%. Found: C, 39.20%; H, 5.10%; N, 1.40%.

### Synthesis of ThCl­(DMF)_4_Mo_5_O_13_(OMe)_4_NO (6-ThCl­(DMF)_4_Mo_5_)

In a 20 mL scintillation vial, (TBA)­[ThCl_2_(DMF)_2_Mo_5_O_13_(OMe)_4_NO] (0.5 g, 0.33 mmol, 1 equiv) was dissolved in a mixture of DCM (2 mL) and DMF (0.5 mL), forming a dark blue solution. To this, a solution of AgOTf (0.084 g, 0.33 mmol, 1 equiv) dissolved in DCM (2 mL) was added quickly with stirring. The mixture quickly turned a lighter blue and solid precipitated. The mixture was stirred at room temperature for 1 h before filtering through a bed of Celite (approx. 1 cm). The DCM was removed under reduced pressure to leave a dark blue oil. This was then treated with Et_2_O (20 mL) with stirring leading to the formation of a light blue powder. The powder was allowed to settle before the solvent was discarded. The powder was washed with Et_2_O (2 × 15 mL) to ensure complete removal of free DMF. The solid was extracted with DCM (10 mL) and filtered through a bed of Celite (approx. 1 cm). The solvent was removed under vacuum to give crude product (0.31 g, 67%) as a blue powder. Blue crystals were obtained by vapor diffusion of Et_2_O into a saturated solution of the product in DCM. ^**1**^H NMR (500 MHz, CD_2_Cl_2_) δ 3.00 (s, 12 H), 3.09 (s, 12H), 4.77 (s, 12 H), 8.04 (s, 4H). ^17^O enriched ThCl­(DMF)_4_Mo_5_O_13_(OMe)_4_NO was obtained by following the procedure described above with ^17^O enriched (TBA)­[ThCl_2_(DMF)_2_Mo_5_O_13_(OMe)_4_NO]. ^17^O NMR (67.8 MHz, CD_2_Cl_2_) δ 13.8 (1 O), 525.7 (4 O), 707.8 (4 O), 919.1 (4 O) (Note: ^17^O enriched samples consistently contained small amounts of **3-ThCl**_**2**_ **(DMF)**_**2**_ **Mo**_**5**_, as evidenced by an additional peak at 717.6 ppm). UV-Vis/NIR (21 ^o^C, DCM): λ_max_ (ε) = 572 nm (61 M^–1^ cm^–1^). Anal. calcd for C_16_H_40_N_5_O_22_ClMo_5_Th·0.25DMF: C, 14.17%; H, 2.96%; N, 5.18%. Found: C, 13.84%; H, 2.69%; N, 5.58%.

### Synthesis of TpTh­(DMF)_2_Mo_5_O_13_(OMe)_4_NO (7-TpTh­(DMF)_2_Mo_5_)

#### Method A, from 6-ThCl­(DMF)_4_Mo_5_

In a 20 mL scintillation vial, ThCl­(DMF)_4_Mo_5_O_13_(OMe)_4_NO (0.45 g, 0.32 mmol, 1 equiv) was suspended in THF (5 mL) and then DMF was added dropwise until a homogeneous blue solution was obtained. To this, a solution of potassium tris­(pyrazolyl)­borate (KTp, 0.081 g, 0.32 mmol, 1 equiv) dissolved in THF (2 mL) was added quickly with stirring. The mixture turned a slightly lighter blue and became cloudy. The mixture was stirred at room temperature for 15 min before filtering through a bed of Celite (approx. 1 cm). The THF was removed under reduced pressure to leave a dark blue oil. This was treated with Et_2_O (20 mL) with stirring leading to the formation of a light blue powder. The powder was allowed to settle before the solvent was discarded. The powder was then washed with Et_2_O (2 × 15 mL) to ensure complete removal of free DMF. The solid was extracted with DCM (10 mL) and filtered through a bed of Celite (approx. 1 cm). The solvent was removed under vacuum to give crude product (0.39 g, 85%) as a blue powder. Blue crystals were obtained by vapor diffusion of Et_2_O into a saturated solution of the product in DCM. ^**1**^H NMR (500 MHz, CD_2_Cl_2_) δ 2.87 (s, 6H), 3.12 (s, 6H), 4.78 (s, 12 H), 6.16 (s, 1 H), 6.23 (s, 2H), 7.64 (s, 1H), 7.71 (s, 2H), 7.80 (s, 1H), 7.94 (s, 4H, overlapping protons associated with Tp and DMF). ^17^O enriched TpTh­(DMF)_2_Mo_5_O_13_(OMe)_4_NO was obtained by following the procedure described above with ^17^O enriched ThCl­(DMF)_4_Mo_5_O_13_(OMe)_4_NO. ^17^O NMR (67.8 MHz, CD_2_Cl_2_) δ 15.9 (1 O), 518.5 (2 O), 522.8 (2 O), 691.4 (2 O), 706.8 (1 O), 731.2 (1 O), 911.4 (1 O), 917.6 (3 O). UV-Vis/NIR (21 °C, DCM): λ_max_ (ε) = 575 nm (57 M^–1^ cm^–1^). Anal. calcd for C_19_H_36_N_9_O_20_BMo_5_Th·DCM: C, 15.82%; H, 2.52%; N, 8.30%. Found: C, 15.65%; H, 2.20%; N, 8.38%.

#### Method B, from 3-ThCl_2_(DMF)_2_Mo_5_

In a 20 mL scintillation vial, (TBA)­[ThCl_2_(DMF)_2_Mo_5_O_13_(OMe)_4_NO] (0.12 g, 0.078 mmol, 1 equiv) was dissolved in a mixture of DMF (0.3 mL) and THF (3 mL), giving a homogeneous blue solution. A solution of KTp (0.02 g, 0.078 mmol, 1 equiv) dissolved in THF (1 mL) was added. The mixture was stirred for 15 min before a solution of Ag­(OTf) (0.02 g, 0.078 mmol, 1 equiv) dissolved in THF (1 mL) was added. The mixture was stirred for a further 30 min during which time a cloudy brown solution formed. The mixture was filtered through a bed of Celite (approx.1 cm), isolating a dark blue/green filtrate. The solvent was removed under vacuum and the obtained solid was triturated with Et_2_O (2 × 5 mL). The solid was dissolved in DCM (5 mL) and was filtered through a bed of Celite (approx.1 cm). The obtained solution was dried under vacuum to isolate crude product (0.078 g, 70%) as a dark blue powder. Blue crystals were obtained by vapor diffusion of Et_2_O into a saturated solution of the product in DCM. ^17^O enriched TpTh­(DMF)_2_Mo_5_O_13_(OMe)_4_NO was obtained by following the procedure described above with ^17^O enriched (TBA)­[ThCl_2_(DMF)_2_Mo_5_O_13_(OMe)_4_NO]. The obtained characterization data matched that given above using method A.

### Physical Measurements

^1^H NMR and ^17^O NMR spectra were recorded at room temperature on a 500 MHz JEOL spectrometer or a 500 MHz Bruker AVANCE spectrometer locked to the signal of deuterated solvents. All chemical shifts are reported relative to SiMe_4_ (for ^1^H NMR spectra) or D_2_O (for ^17^O NMR spectra) by using the chosen deuterated solvent as an internal standard. Cyclic voltammetry was performed using a three-electrode setup inside a positive-pressure glovebox (MBraun UniLab, USA) using a Bio-Logic SP 150 potentiostat/galvanostat. The concentrations of the cluster and the supporting electrolyte (TBAPF_6_) were kept at 1 mM and 100 mM, respectively, throughout all measurements. Cyclic voltammograms (CVs) were recorded using a 3 mm diameter glassy carbon working electrode (CH Instruments, USA), a Pt wire auxiliary electrode (CH Instruments, USA), and a Ag/Ag^+^ nonaqueous reference electrode with 0.01 M AgNO_3_ in 0.1 M TBAPF_6_ in MeCN (BASi, USA). Ferrocene was used as an internal standard after completion of the measurements, and potentials were referenced versus the Fc^+/0^ couple. Electronic absorption measurements were recorded at room temperature in anhydrous DCM in sealed 1 cm quartz cuvettes using an Agilent Cary 6000i UV–vis-NIR spectrophotometer. Elemental analysis data were obtained from the Elemental Analysis Facility at The University of Rochester. Microanalysis samples were weighed with a PerkinElmer model AD6000 autobalance, and their compositions were determined with a PerkinElmer 2400 series II analyzer. Air-sensitive samples were handled in a VAC Atmospheres glovebox.

### X-ray Crystallography

Crystals were placed onto a nylon loop and mounted on a Rigaku XtaLAB Synergy-S Dualflex diffractometer equipped with a HyPix-6000HE HPC area detector for data collection at 100.00(10) K. A preliminary set of cell constants and an orientation matrix were calculated from a small sampling of reflections.^62^ A short pre-experiment was run, from which an optimal data collection strategy was determined. The full data collection was carried out using a PhotonJet (Mo) X-ray source. After the intensity data were corrected for absorption, the final cell constants were calculated from the xyz centroids of the strong reflections from the actual data collection after integration.^62^ The structure was solved using SHELXT or isomorphic replacement and refined using SHELXL.^63,64^ Most or all non-hydrogen atoms were assigned from the solution. Full-matrix least squares/difference Fourier cycles were performed which located any remaining non-hydrogen atoms. All non-hydrogen atoms were refined with anisotropic displacement parameters. All hydrogen atoms were placed in ideal positions and refined as riding atoms with relative isotropic displacement parameters.

## Results and Discussion

### Synthesis and Characterization of (TBA)­[ThCl_2_(DMF)_2_Mo_5_O_13_(OMe)_4_NO]

A prerequisite to studying the coordination chemistry and reactivity of actinides supported by POMs is formation of a stable complex in which the actinide coordination sphere is not saturated by the POM ligand(s). We hypothesized that this is most readily achieved by *avoiding* the formation of sandwich-type An­(POM)_2_ complexes, instead targeting the 1:1 An-POM complex. As mentioned in the introduction, prior work has demonstrated equilibration of 1:2 An­(POM)_2_ complexes with 1:1 (H_2_O)_X_An­(POM) in aqueous media, revealing that solvent coordination to the An stabilizes the 1:1 complex.^32,51–53^ We therefore reasoned that use of a strongly coordinating non-aqueous solvent in the synthesis of an An-polyoxoalkoxide complex could stabilize the 1:1 (solvent)_X_An­(POM) complex relative to the 1:2 An­(POM)_2_ complex. We opted to investigate this hypothesis using thorium (Th^IV^) as our model actinide, as its closed shell electron configuration allows convenient reaction monitoring using ^17^O and ^1^H NMR spectroscopy. As a solvent, we chose to use dimethylformamide (DMF) due to its high affinity for An^IV^ centers, with numerous DMF solvated complexes reported in the literature (e.g., U­(DMF)_9_I_4_).^65–70^

We first examined the reactivity of the lacunary precursor **1-NaMo**_**5**_ toward Th^IV^ in DMF (Scheme 1, Method A). Treatment of a DMF solution of **1-NaMo**_**5**_ with 1 eq. of ThCl_4_·2DME results in the formation of a dark blue solution. Precipitation of this species by addition of Et_2_O enables isolation of a light purple powder. The crude material was then extracted into MeCN to separate it from NaCl, before crystallization from DMF/Et_2_O.

**Scheme 1:**
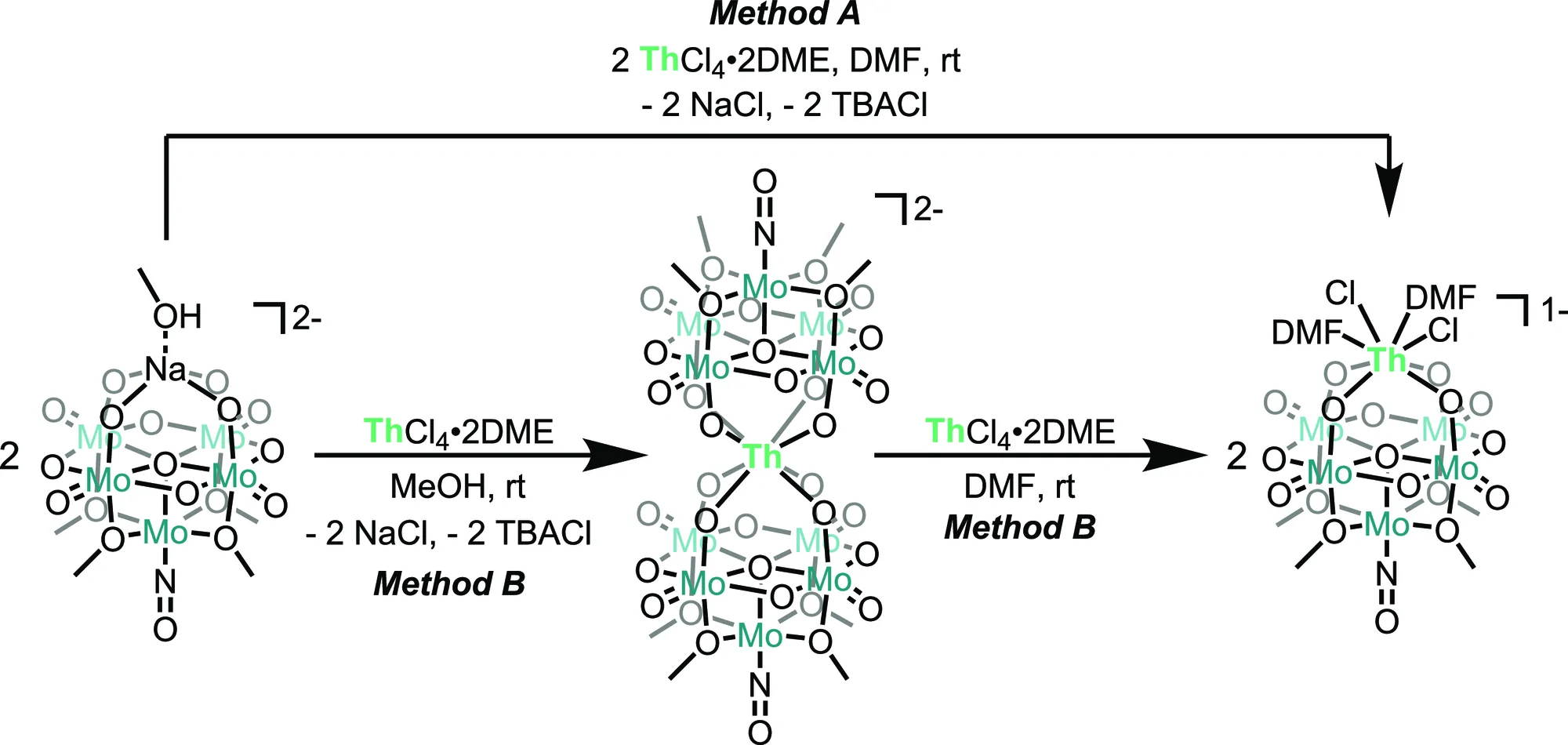
Synthetic Approaches used to Produce (TBA)­[ThCl_2_(DMF)_2_Mo_5_O_13_(OMe)_4_NO] (**3-ThCl**_**2**_ **(DMF)**_**2**_ **Mo**_**5**_)

The ^1^H and ^17^O NMR spectra of the isolated material are shown in Figure 2. The ^1^H NMR spectrum shows a singlet at 4.73 ppm which is characteristic of the -OCH_3_ groups of [Mo_5_O_13_(OMe)_4_NO]^3–^ bound to a diamagnetic M^IV^ cation. This signal possesses the same chemical shift as that observed in the ^1^H NMR spectrum of **2-Th­(Mo**_**5**_ **)**_**2**_ (as well as the Zr^IV^ and Hf^IV^ derivatives). Additional peaks at 3.06 ppm (6H), 3.09 ppm (6H), and 8.12 ppm (2H) are consistent with the presence of DMF directly coordinated to the polyoxoalkoxide complex, with integration supporting the presence of two DMF molecules per {Mo_5_O_13_(OMe)_4_NO}^3–^ unit. The remaining features (1.04, 1.49, 1.70, and 3.23 ppm) are assigned to the TBA counterion, with integration suggesting the presence of a single TBA cation per {Mo_5_O_13_(OMe)_4_NO}^3–^ unit. These results support the formation of (TBA)­[ThCl_2_(DMF)_2_Mo_5_O_13_(OMe)_4_NO] (**3-ThCl**_**2**_ **(DMF)**_**2**_ **Mo**_**5**_), with the presence of two chloride anions required to balance the overall charge. ^17^O enriched material was obtained by following the same procedure with ^17^O enriched **1-NaMo**_**5**_, obtained by stirring **1-NaMo**_**5**_ with 40% ^17^O enriched H_2_O at 50 °C, according to our previously reported method.^57^ This allows ^17^O labelling of the unique oxo-environments within the structure via O atom exchange, with only the oxygen nuclei of the –OCH_3_ groups and the {Mo-NO}^4^ unit not enriched. The ^17^O NMR spectrum obtained from the reaction of ^17^O enriched **1-NaMo**_**5**_ with ThCl_4_·2DME in DMF (Figure 2, bottom) further supports this formation of **3-ThCl**_**2**_ **(DMF)**_**2**_ **Mo**_**5**_, with four peaks in a 4:4:4:1 ratio confirming the integrity of the {Mo_5_O_13_(OMe)_4_NO}^3–^ ligand. The chemical shift of the oxygens associated with Mo–O–M bridges (red peak), which are the most sensitive to the nature of the heterometal, is very close (ca. 0.7 ppm) to that of **2-Th­(Mo**_**5**_ **)**_**2**_. However, the remaining peaks associated with the terminal Mo=O, bridging Mo–O–Mo, and central μ_5_-O nuclei are shifted (ca. 1-8 ppm). Collectively, these data all point toward the formation of the new 1:1 thorium-polyoxoalkoxide complex **3-ThCl**_**2**_ **(DMF)**_**2**_ **Mo**_**5**_.

**Figure 2:**
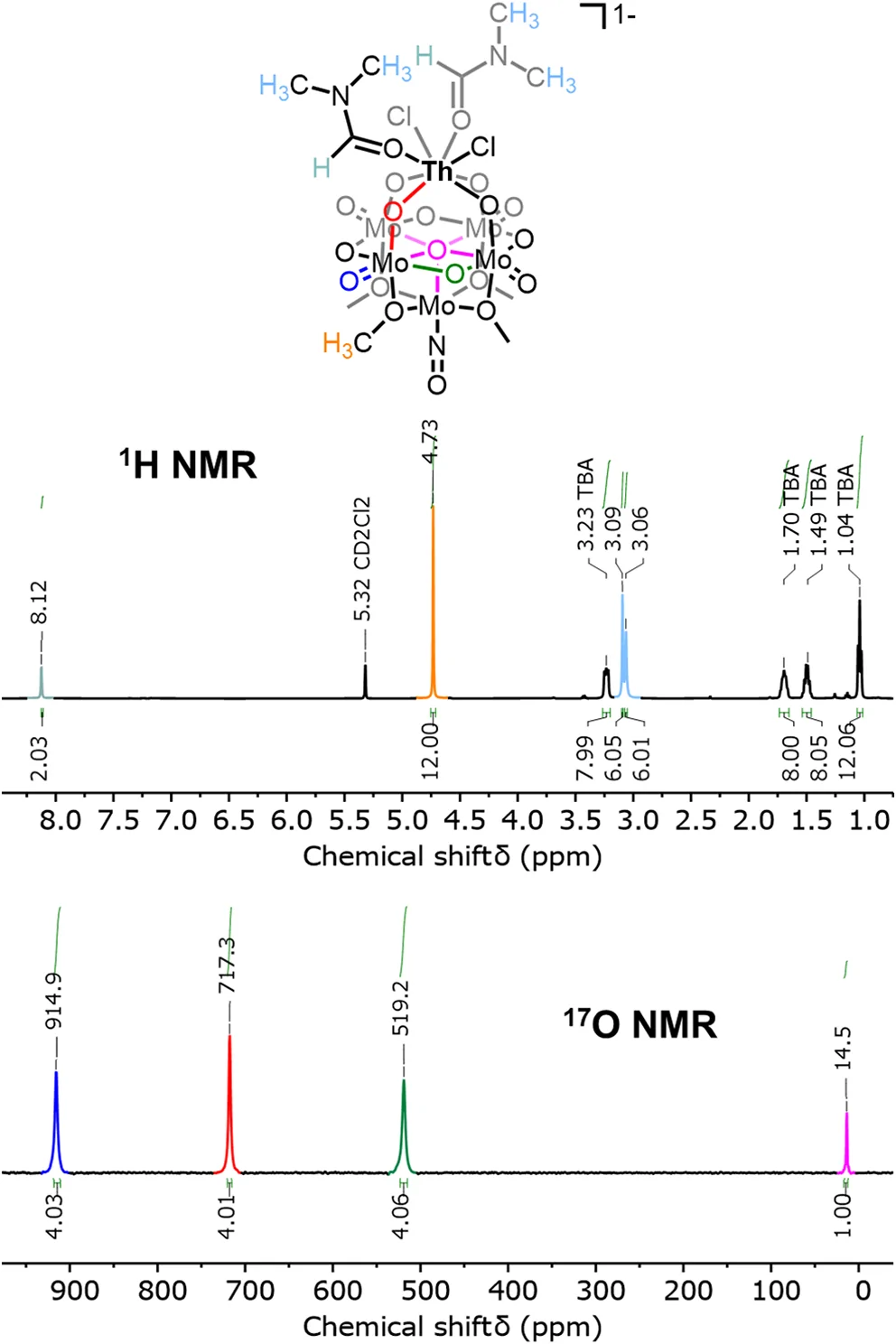
^1^H NMR (top) and ^17^O NMR (bottom) spectra of **3-ThCl**_**2**_ **(DMF)**_**2**_ **Mo**_**5**_. Peaks in the NMR spectra are color matched to the appropriate nuclei in the representative structure above to show assignments. Spectra were recorded at 20.8 °C in CD_2_Cl_2_. The corresponding spectra obtained in CD_3_CN are given in Figures S3 and S4.

To confirm formation of the targeted 1:1 thorium-polyoxoalkoxide, single crystal X-ray diffraction (SCXRD) experiments were pursued. Blue-purple crystals were obtained by vapor diffusion of Et_2_O into a saturated solution of **3-ThCl**_**2**_ **(DMF)**_**2**_ **Mo**_**5**_ dissolved in MeCN. Diffraction experiments yield, after refinement of the data, the structure shown in Figure 3, a 1:1 Th:{Mo_5_} complex in which the other face of the actinide center is stabilized through the binding of two chloride ions and two DMF solvent molecules. The observed structure reinforces the fact that strong donor solvents are important for the stabilization of 1:1 An-POM complexes.

**Figure 3:**
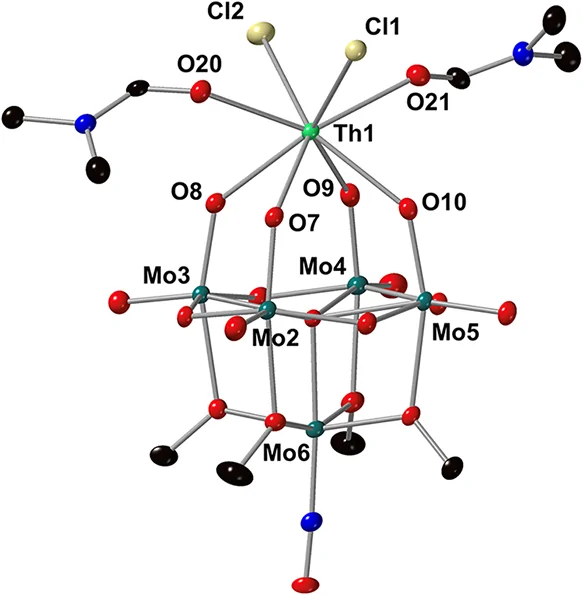
SCXRD structure of **3-ThCl**_**2**_ **(DMF)**_**2**_ **Mo**_**5**_ with probability ellipsoids set at 50%. Cations and solvent molecules have been removed for clarity.

**3-ThCl**_**2**_ **(DMF)**_**2**_ **Mo**_**5**_ possesses a distorted square antiprismatic (SAPR) geometry at the Th center. The {Mo_5_} ligand binds to the Th center via four bridging oxygen atoms (O7–O10), with an average Th–O bond length of 2.437(5) Å. This is slightly longer than the Th–O bond distances observed in **2-Th­(Mo**_**5**_ **)**_**2**_ (2.410(15) Å), suggesting a slightly weaker interaction between the Th^IV^ center and the {Mo_5_} ligand in **3-ThCl**_**2**_ **(DMF)**_**2**_ **Mo**_**5**_ as compared to the sandwich-type assembly. The four oxygen atoms of the {Mo_5_} ligand which bind to Th form an almost perfect plane, possessing a root-mean-square deviation (RMSD) of only 0.001 Å. In contrast, the plane formed by the two oxygen atoms of the DMF molecules bound to Th and the two chloride ions is distorted (RMSD of 0.395 Å). This is likely caused by the discrepancy in Th–Cl (2.739(8) Å) and Th–O_DMF_ (2.420(18) Å) bond lengths, as well as the differing steric profiles of the ligands. Similar distortions have been observed in other structures containing {AnCl_2_} fragments;^71–75^ in these cases, the observed geometries maximize separation between monodentate ligands. The deviation between the observed Th geometry and an ideal SAPR geometry can be quantified using continuous shape measure (CShM) analysis.^76–81^ This approach provides a set of CShM values that compare the observed coordination environment to idealized geometries of the same coordination number, with values approaching zero indicating a closer match to the ideal structure. Performing this analysis on **3-ThCl**_**2**_ **(DMF)**_**2**_ **Mo**_**5**_ gives a SAPR CShM value of 1.657, with only a slightly higher value obtained for triangular dodecahedron (CShM value = 1.976) (Table S2). These values are much higher than the SAPR CShM value (0.419) obtained for **2-Th­(Mo**_**5**_ **)**_**2**_, highlighting that replacement of a {Mo_5_} unit with a mixture of monodentate ligands leads to deviations from “ideal” coordination geometries at the actinide.^56^

Given **3-ThCl**_**2**_ **(DMF)**_**2**_ **Mo**_**5**_ is the dominant product from the reaction of **1-NaMo**_**5**_ and ThCl_4_·2DME in DMF, and it is stable once formed, we envisaged that it may be possible to drive conversion of the 1:2 complex **2-Th­(Mo**_**5**_ **)**_**2**_ to the 1:1 complex **3-ThCl**_**2**_ **(DMF)**_**2**_ **Mo**_**5**_ by adding ThCl_4_·2DME to a solution of the sandwich-type complex dissolved in DMF (Scheme 1, Method B). Doing this leads to an instant color change from purple to dark blue. *In-situ* characterization by ^17^O NMR spectroscopy (Figure S5) shows the characteristic 4:4:4:1 pattern (+DMF) with peaks in positions similar to those observed during reactions of **1-NaMo**_**5**_ with ThCl_4_·2DME (Figure S2). Addition of an excess of Et_2_O to this solution drives precipitation of a light purple powder. Characterization of this powder, after drying, by ^1^H and ^17^O NMR spectroscopy (Figures S6 and S7) gives identical spectra to those given in Figure 2, illustrating that **3-ThCl**_**2**_ **(DMF)**_**2**_ **Mo**_**5**_ can be accessed using both methods. Though this method requires the additional step of preparing **2-Th­(Mo**_**5**_ **)**_**2**_, the overall yield is higher (77% yield over two steps vs. 62% yield in one step) and the work-up procedure is more straightforward.^56^

Successful syntheses of **3-ThCl**_**2**_ **(DMF)**_**2**_ **Mo**_**5**_ reveal that stabilization of 1:1 An-POM complexes is promoted by the presence of neutral ligands that bind strongly to the actinide center. We found that dissolution of **3-ThCl**_**2**_ **(DMF)**_**2**_ **Mo**_**5**_ in a range of solvents (e.g., MeCN, THF, and DCM) failed to drive the displacement of the DMF ligands of **3-ThCl**_**2**_ **(DMF)**_**2**_ **Mo**_**5**_ (Figure 2, S3, and S8), with **3-ThCl**_**2**_ **(DMF)**_**2**_ **Mo**_**5**_ often readily crystallizing from these solvents. This demonstrates both that **3-ThCl**_**2**_ **(DMF)**_**2**_ **Mo**_**5**_ is stable with respect to reformation of the sandwich-type complex **2-Th­(Mo**_**5**_ **)**_**2**_ and that a substantial driving force is required to drive exchange of the DMF ligands of **3-ThCl**_**2**_ **(DMF)**_**2**_ **Mo**_**5**_ with other neutral donors.

### Synthesis of Asymmetric Complexes from (TBA)­[ThCl_2_(DMF)_2_Mo_5_O_13_(OMe)_4_NO]

As alluded to in the introduction, a large proportion of An-POM complexes are symmetric 1:2 complexes in which the An is sandwiched between two POM ligands. The inability to cleanly produce 1:1 An-POM complexes in aqueous media has hindered the ability to form asymmetric sandwich-type complexes in which the actinide center is stabilized by two different multidentate ligands, with only a couple of reported examples.^82,83^ Complexes of this type are very interesting as they allow the interactions between the An and POM to be studied as a function of ligand variation. Furthermore, the coordination environment at the actinide center can be precisely controlled based on the choice of ligand. This is significant as symmetric An-POM sandwich-type complexes invariably possess SAPR coordination environments at the An center, with only small distortions introduced via changes to the secondary coordination sphere.^27,37,51^ Recent work by Carter and co-workers highlights that distortions away from perfect D_4d_ SAPR geometries can have important consequences for the magnetic (and vibrational) properties of An-POM (and lanthanide-POM) complexes and thus the ability to precisely control the An coordination environment is desirable.^37,84,85^

Given **3-ThCl**_**2**_ **(DMF)**_**2**_ **Mo**_**5**_ resists exchange of its DMF ligands with common solvents that can act as neutral ligands (i.e., MeCN or THF), we were first keen to verify if neutral ligand exchange with **3-ThCl**_**2**_ **(DMF)**_**2**_ **Mo**_**5**_ was possible. To do this we identified a ligand which would be likely to drive the displacement of DMF from **3-ThCl**_**2**_ **(DMF)**_**2**_ **Mo**_**5**_. One such ligand is 2,2-bipyridine (bipy). This bidentate ligand binds through basic nitrogen lone pairs, with its binding affinity enhanced via the chelate effect. This has made bipy a common ligand in actinide chemistry, with several examples of the An^IV^-bipy complexes present in the literature.^86–88^ To assess the ability of bipy to displace the DMF molecules in **3-ThCl**_**2**_ **(DMF)**_**2**_ **Mo**_**5**_, a solution of **3-ThCl**_**2**_ **(DMF)**_**2**_ **Mo**_**5**_ in MeCN was treated with two equivalents of bipy (Scheme 2). No immediate color change was observed and therefore the outcome of the reaction was assessed using ^1^H and ^17^O NMR spectroscopy (Figure 4). After work-up (see Experimental Section for more details) and recrystallization from DCM/Et_2_O, the ^1^H NMR spectrum (Figure 4, top) shows the same characteristic shift associated with the −OCH_3_ of [Mo_5_O_13_(OMe)_4_NO]^3–^ at 4.70 ppm (12H), though it is slightly shifted compared to the corresponding peak in **2-Th­(Mo**_**5**_ **)**_**2**_ and **3-ThCl**_**2**_ **(DMF)**_**2**_ **Mo**_**5**_.^56^ Importantly, the peaks at 3.06, 3.09, and 8.12 ppm, which are assigned to the DMF ligands in **3-ThCl**_**2**_ **(DMF)**_**2**_ **Mo**_**5**_ are no longer present; instead, four new resonances are observed at 7.73 (2H), 8.18 (2H), 8.33 (2H), and 9.28 (2H) ppm (see Figure 4, top, inset). These signals are assigned to the four unique proton environments of bipy, with the intensities and chemical shifts supporting the binding of a single bipy ligand to the Th^IV^ center.^86,87^ The remaining signals in the spectrum are assigned to the TBA counterion (and residual Et_2_O). Again, integration of the resonances associated with TBA vs. that of the −OCH_3_ peak of {Mo_5_} suggests one TBA ion per {Mo_5_} unit, supporting that the two chloride anions bound to Th in **3-ThCl**_**2**_ **(DMF)**_**2**_ **Mo**_**5**_ have been retained.

**Figure 4:**
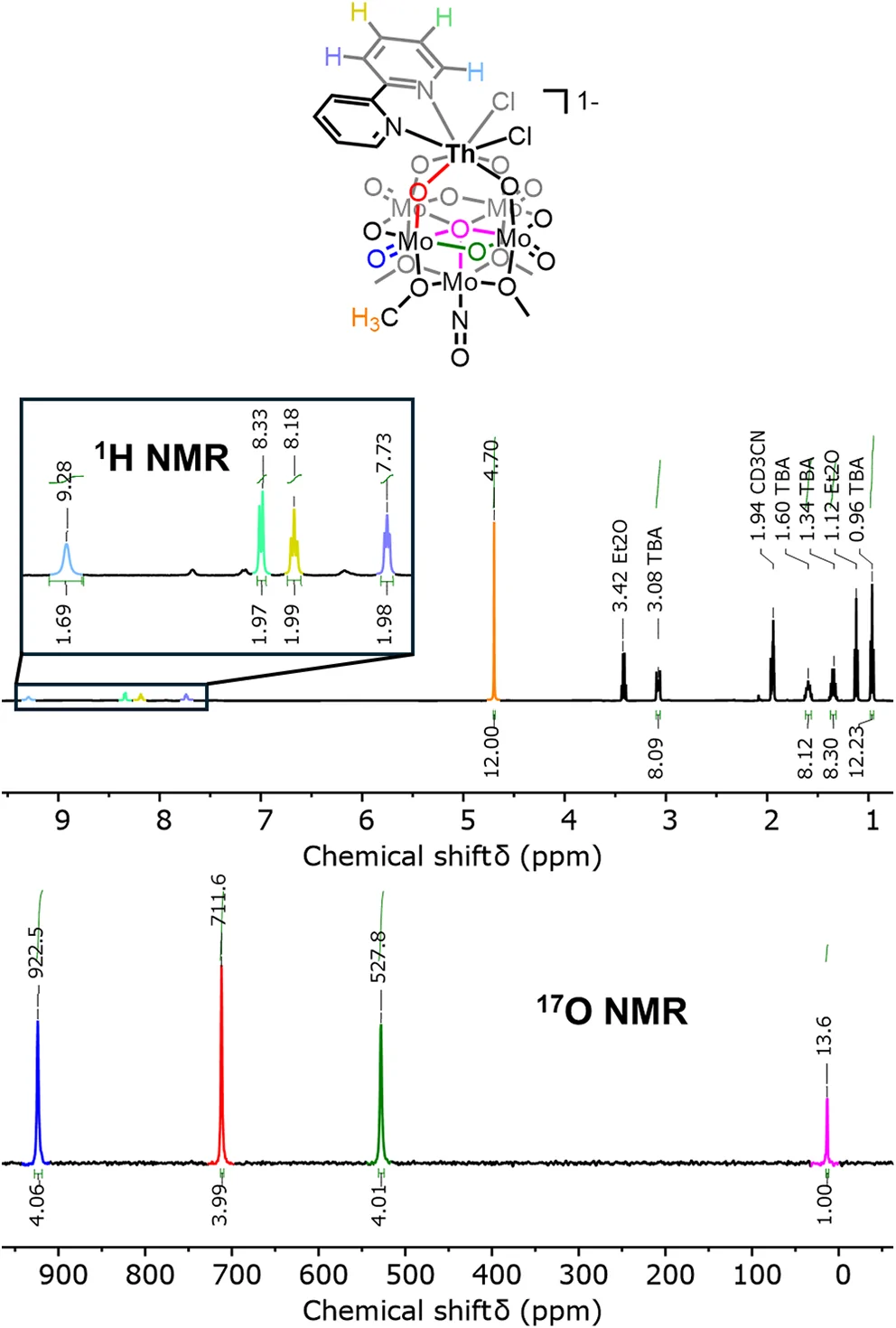
^1^H NMR (top) and ^17^O NMR (bottom) spectra of **4-ThCl**_**2**_ **(bipy)­Mo**_**5**_. Peaks in the NMR spectra are color matched to the appropriate nuclei in the representative structure above to show assignments. Spectra were recorded at 20.8 °C in CD_3_CN.

**Scheme 2:**
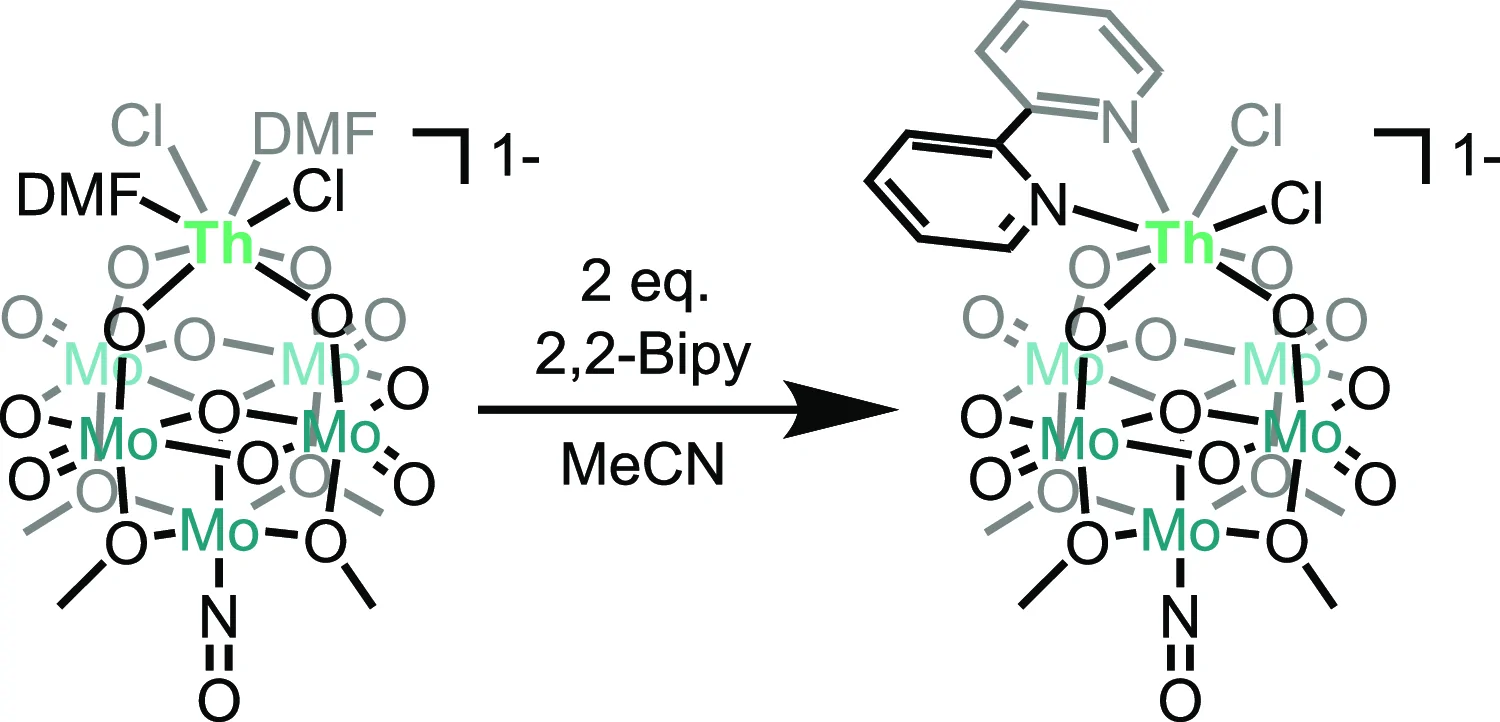
Reactivity of **3-ThCl**_**2**_ **(DMF)**_**2**_ **Mo**_**5**_ toward Excess 2,2-Bipyridine

The ^17^O NMR spectrum (Figure 4, bottom) shows the characteristic 4:4:4:1 pattern caused by the four unique oxo environments of {Mo_5_}. The peaks assigned to the terminal Mo=O (blue) and bridging Mo–O–Mo (green) nuclei are shifted slightly downfield (ca. 7–8 ppm), while the resonance associated with the Mo–O–Th (red) nuclei is shifted upfield by approximately 6 ppm relative to the corresponding peaks in the ^17^O NMR spectrum of **3-ThCl**_**2**_ **(DMF)**_**2**_ **Mo**_**5**_. These shifts are indicative of a stronger interaction between Th and the {Mo_5_} ligand upon replacement of the DMF ligands with bipy. Interestingly, the appearance of a single resonance for the Mo=O, Mo–O–Mo, and Mo–O–Th nuclei (and the −OCH_3_ groups in the ^1^H NMR spectrum) suggests that, in solution, the coordination of bipy/Cl to the Th^IV^ center is dynamic in some way (e.g., through rotation or self-exchange of ligands). This is because strict adherence to the structure shown at the top of Figure 4 would impose C_S_ symmetry and thus lead to a more complex spectrum.

Collectively, the NMR spectra discussed above support formation of (TBA)­[ThCl_2_(bipy)­Mo_5_O_13_(OMe)_4_NO] (**4-ThCl**_**2**_ **(bipy)­Mo**_**5**_). To further confirm this, single crystals of the complex were obtained via vapor diffusion of Et_2_O into a saturated solution of the complex dissolved in DCM and the crystals were subsequently analyzed by SCXRD. The structure obtained, after data refinement, is shown in Figure 5. As expected, the obtained complex features a single Th^IV^ center bound to one {Mo_5_} ligand, one bipy ligand, and two chloride ions. The chelating nature of bipy enforces a “cis” arrangement of the chloride ions, contrasting the arrangement in **3-ThCl**_**2**_ **(DMF)**_**2**_ **Mo**_**5**_, where the chloride ions were found opposite to each other. The average Th–O bond length is 2.408(2) Å, which is shorter than the Th–O bond distances in **3-ThCl**_**2**_ **(DMF)**_**2**_ **Mo**_**5**_ and more similar to those observed in **2-Th­(Mo**_**5**_ **)**_**2**_. The Th–O bond contraction observed in **4-ThCl**_**2**_ **(bipy)­Mo**_**5**_ (relative to **3-ThCl**_**2**_ **(DMF)**_**2**_ **Mo**_**5**_) verifies the changes in the ^17^O NMR spectrum which indicate a stronger interaction between Th^IV^ and the {Mo_5_} unit in the bipy-ligated complex. Further analysis of the structure of **4-ThCl**_**2**_ **(bipy)­Mo**_**5**_ reveals that binding bipy leads to a more ideal SAPR environment at Th, with CShM analysis giving a SAPR CShM value of 0.991 (Table S4). This value is significantly lower than the value obtained for **3-ThCl**_**2**_ **(DMF)**_**2**_ **Mo**_**5**_ (1.657) but still higher than the value obtained for **2-Th­(Mo**_**5**_ **)**_**2**_ (0.419). This shows that replacement of monodentate DMF ligands with a bidentate bipy ligand leads to a more ideal SAPR Th coordination geometry, however the variable bond metrics that accompany the mixed ligand environment (Th–O = 2.408(2) Å; Th–N = 2.632(3) Å; Th–Cl = 2.7384(8) Å) prevent formation of a perfect SAPR, which is more closely approximated by the symmetrical complex **2-Th­(Mo**_**5**_ **)**_**2**_.

**Figure 5:**
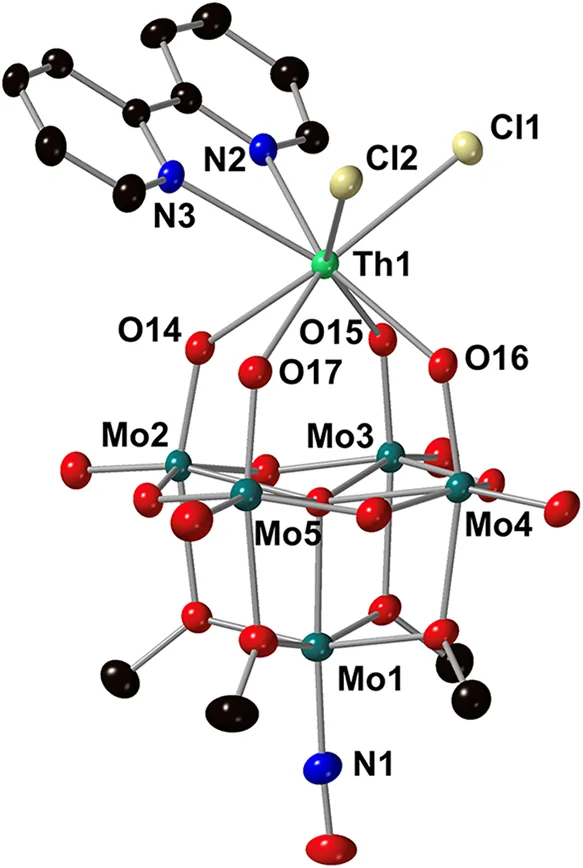
SCXRD structure of **4-ThCl**_**2**_ **(bipy)­Mo**_**5**_ with probability ellipsoids set at 50%. Cations and solvent molecules have been removed for clarity.

After successful displacement of the neutral ligands **3-ThCl**_**2**_ **(DMF)**_**2**_ **Mo**_**5**_, we next turned our attention to the reactivity of the Th–Cl units. We opted to first investigate reactivity toward a dianionic multidentate ligand, hypothesizing that such a ligand could be installed cleanly with concomitant loss of two equivalents of MCl (M = alkali metal). The ligand chosen was 1,3-dimethoxy-4-*tert*-butylcalix­[4]­arene (referred to as CalixH_2_ for the remainder of this work). This ligand has recently been used to synthesize asymmetric lanthanide-polyoxoalkoxide complexes with the general formula [CalixMMo_5_O_13_(OMe)_4_NO]^2–^ (M = Y, Nd, Gd, Dy, and Er) and therefore may be expected to stabilize analogous complexes with a central Th^IV^ ion.^89–91^ The ligand was first deprotonated by treatment with Na/naphthalene in THF, following literature methods, to produce the dianionic ligand CalixNa_2_.^61^ Solid CalixNa_2_ was then treated with a solution of **3-ThCl**_**2**_ **(DMF)**_**2**_ **Mo**_**5**_ dissolved in THF (Scheme 3). This led to gradual dissolution of the calixarene ligand and a color change to pale yellow as the reaction proceeded. Following work-up (see Experimental Section for details), formation of the target asymmetric complex, (TBA)­[CalixThMo_5_O_13_(OMe)_4_NO] (**5-** **CalixThMo**_**5**_), was confirmed using ^1^H and ^17^O NMR spectroscopy. The ^1^H NMR spectrum of **5-** **CalixThMo**_**5**_ showed a total of 12 resonances, consistent with the expected *C*_2*V*_ symmetry of the product, with peaks readily assigned to the {Calix} ligand, the {Mo_5_} unit, and the TBA counterion (Figure S9). The ^17^O NMR spectrum (Figure S10) also supports the formation of **5-** **CalixThMo**_**5**_. The relative orientation of the {Calix} ligand with respect to the {Mo_5_} leads to a single resonance associated with the Mo=O nuclei (909.2 ppm, 4 O) and a single resonance associated with the Th–O–Mo nuclei (722.5 ppm, 4 O). However, two resonances are observed for the Mo–O–Mo nuclei (501.4 and 513.8 ppm, 2 O each). The nuclei within this group are rendered inequivalent via their positions directly below the different types of donor atom of the {Calix} (i.e., Ar–O vs Ar-OCH_3_). A final resonance assigned to the central μ_5_-O is observed at 13.8 ppm.

**Scheme 3:**
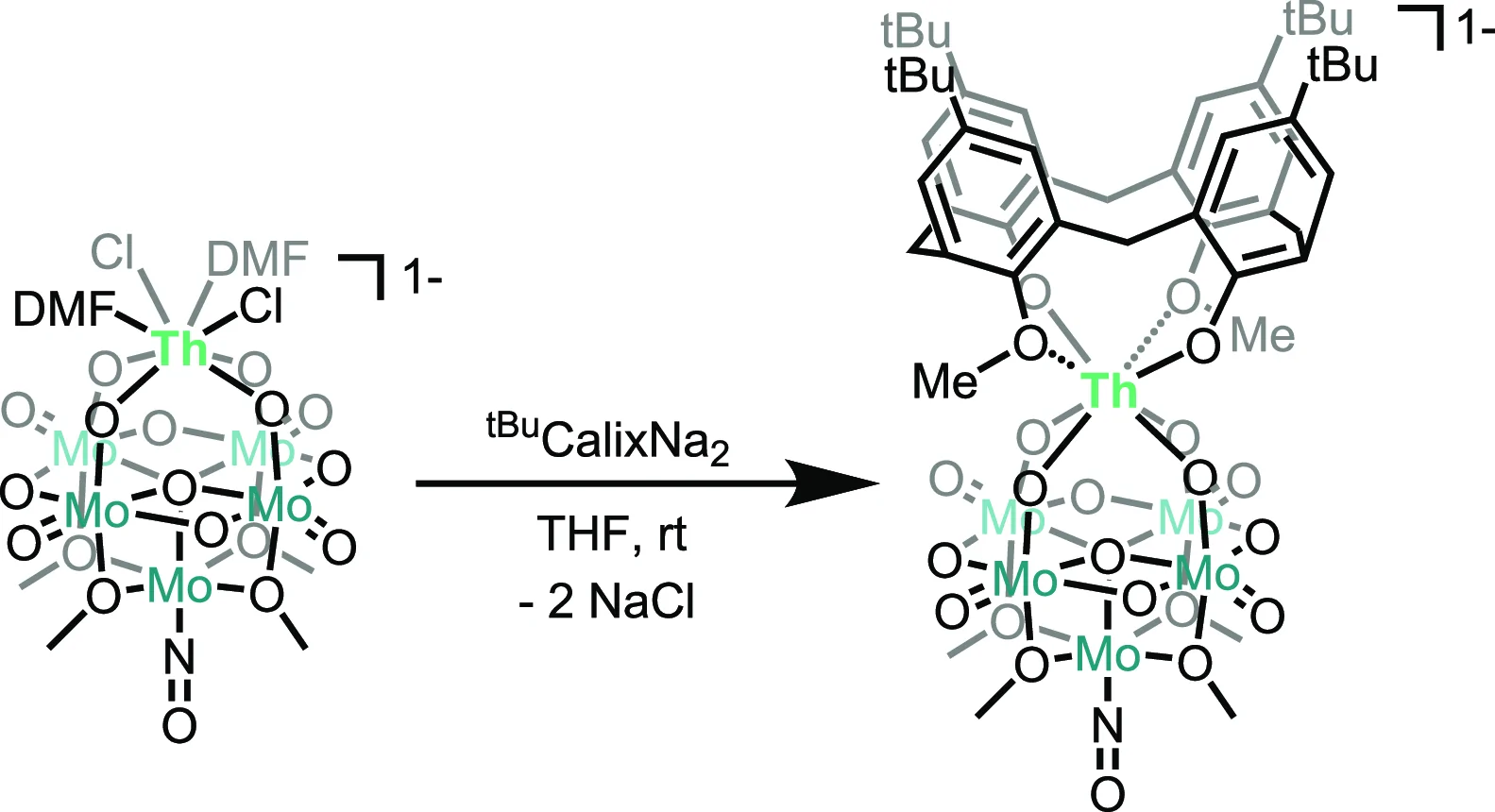
Synthetic Approach Used to Synthesize **5-** **CalixThMo**_**5**_ from **3-ThCl**_**2**_ **(DMF)**_**2**_ **Mo**_**5**_

To further confirm the formation of **5-** **CalixThMo**_**5**_, single crystals of the complex were grown by vapor diffusion of Et_2_O into a saturated solution of the complex dissolved in DCM. SCXRD experiments yielded, after refinement of the data, the structure shown in Figure 6a. The structure confirms the formation of the asymmetric complex, with the {Calix} ligand encapsulating a single DCM molecule, in line with previous reports of small molecule capture by calixarenes.^92–94^

**Figure 6:**
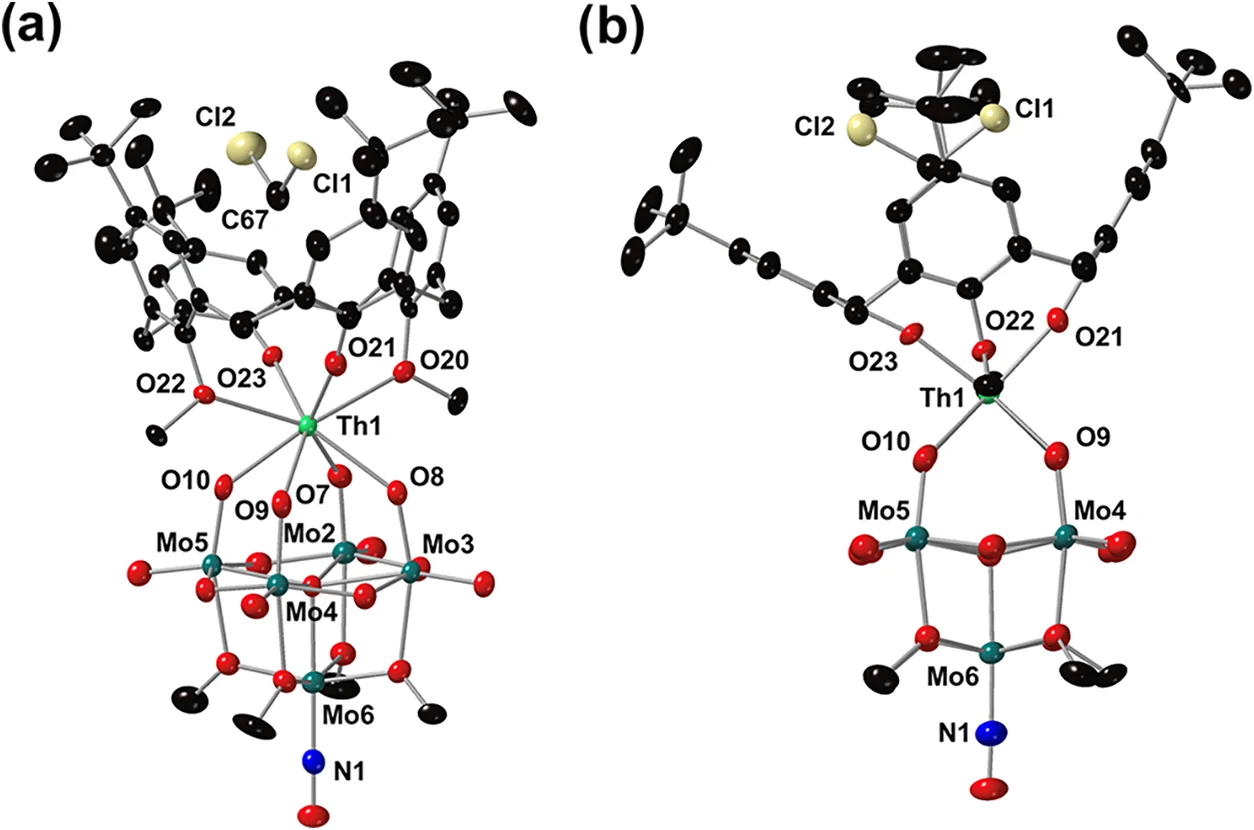
(a) SCXRD structure of **5-** **CalixThMo**_**5**_ with probability ellipsoids set at 50%. (b) A side view of the structure highlighting the “tilted” Calix ligand. The tetrabutylammonium cation and disorder of the -OCH_3_ and ^t^Bu groups have been omitted for clarity.

Much like in the structure of **3-ThCl**_**2**_ **(DMF)**_**2**_ **Mo**_**5**_, the Th coordination environment deviates significantly from SAPR (CShM = 2.231) because of the variable Th–O bond distances caused by the presence of two distinct types of oxygen donor on the {Calix} ligand (Table S6). The Th–O21 (2.187(5) Å) and Th–O23 (2.144(5) Å) bond distances are drastically shorter than the others present in the complex which, when considered in tandem with the more linear Th–O–C angles (167.2(4)° and 168.6(6)°), suggests the presence of additional Th–O π-bonding. Similar distances and bond angles have been previously observed in uranium calix[4]­arene complexes.^73,95^ This contrasts the Th–O20 (2.603(7) Å) and Th–O22 (2.595(5) Å) bond distances which are the longest in the complex. These bonds are even longer than typical Th^IV^-L (L = THF or MeOH) bond distances, suggesting only a weak dative interaction between the Th and the –OCH_3_ groups of Calix. These distortions lead to a Th coordination geometry which is best described as biaugmented trigonal prismatic (Johnson solid J_50_), with a CShM value of 0.9751 (Table S6).^96^

Examining the bonding interactions between the Th and the {Mo_5_} ligand reveals these interactions are slightly weaker than those observed in the other complexes discussed in this work, with an average Th-(O7-10) bond distance of 2.465(16) Å. This slight increase in Th-{Mo_5_} bond distance could be caused by stronger interactions between tetradentate Calix ligand and Th center compared to the combinations of neutral ligands and chloride ions present in **3-ThCl**_**2**_ **(DMF)**_**2**_ **Mo**_**5**_ and **4-ThCl**_**2**_ **(bipy)­Mo**_**5**_, which pull the Th center away from the polyoxoalkoxide ligand. Interestingly, the {Calix} ligand appears slightly tilted with respect to the {Mo_5_} ligand, with Figure 6b highlighting this distortion. This tilting does not lead to significant asymmetry in the Th coordination environment and therefore is likely a consequence of molecule packing within the lattice, with several {Calix} ligands lying close in space in the extended structure.

Utilizing {Calix} to synthesize an asymmetric Th-POM complex proved the utility of **3-ThCl**_**2**_ **(DMF)**_**2**_ **Mo**_**5**_ for producing asymmetric sandwich-type complexes. However, the tetradentate nature of {Calix} in many ways mimics the coordination preferences of typical POM ligands, with a ThO_8_ unit at the center of the sandwich-type complex. We were keen to explore whether ligands with different coordination preferences could also be employed which would expand the range of coordination environments accessible for An-POM systems. Our group has recently reported the synthesis of a series of asymmetric U-POM complexes with the form (TBA)_*x*_[TpU^IV^EW_11_O_39_] (E = P; *x* = 4, E = Si; *x* = 5, Tp = tris­(pyrazolyl)­borate), examples of An-POM complexes which contain a seven coordinate An center.^78^ Inspired by this success, we set out to examine the reactivity of our 1:1 Th-{Mo_5_} system towards Tp.

Given Tp is a monoanionic ligand, we first performed halide abstraction on **3-ThCl**_**2**_ **(DMF)**_**2**_ **Mo**_**5**_ to leave only one chloride available for reaction with Tp, which is commercially available as its potassium salt KTp. Halide abstraction was achieved via treatment of **3-ThCl**_**2**_ **(DMF)**_**2**_ **Mo**_**5**_ with silver triflate (AgOTf) in a mixed DCM/DMF solvent system (Scheme 4, 1^st^ step). This led to the almost immediate precipitation of AgCl from the reaction mixture which was separated by filtration. The reaction mixture was concentrated and then the crude product was precipitated by the addition of an excess of Et_2_O. The product was then extracted into DCM to ensure complete removal of AgCl. Following work-up (see Experimental Section for details), the success of the reaction was assessed using ^1^H and ^17^O NMR spectroscopy (Figures S11 and S12). The ^1^H NMR spectrum contains major peaks at 3.00, 3.09, 4.77, and 8.09 ppm. These peaks are readily assigned to the –OCH_3_ groups of the {Mo_5_} ligand (4.77 ppm) and DMF (3.00, 3.07, 8.09). The absence of any peaks associated with TBA cations indicates that loss of AgCl is accompanied by loss of TBA­(OTf), ruling out the possibility of Th–Cl → Th-OTf exchange. This is further supported by the downfield shift in the position of the –OMe peak (4.73 in **3-ThCl**_**2**_ **(DMF)**_**2**_ **Mo**_**5**_), which is indicative of a reduction in the anionic charge of the system (i.e., moving from 1– to neutral). Integration of the respective {Mo_5_} and DMF peaks indicates the presence of four DMF molecules, supporting the formation of the neutral complex ThCl­(DMF)_4_Mo_5_O_13_(OMe)_4_NO (**6-ThCl­(DMF)**_**4**_ **Mo**_**5**_). The ^17^O NMR spectrum (Figure S12) also shows four resonances in the characteristic 4:4:4:1 pattern. The peaks attributed to Mo=O (919.6 ppm) and Mo–O–Mo (524.3 ppm) nuclei are both shifted downfiled, compared to the corresponding peaks in the ^17^O NMR spectrum of **3-ThCl**_**2**_ **(DMF)**_**2**_ **Mo**_**5**_, consistent with moving from a 1– to neutral complex.

**Scheme 4:**
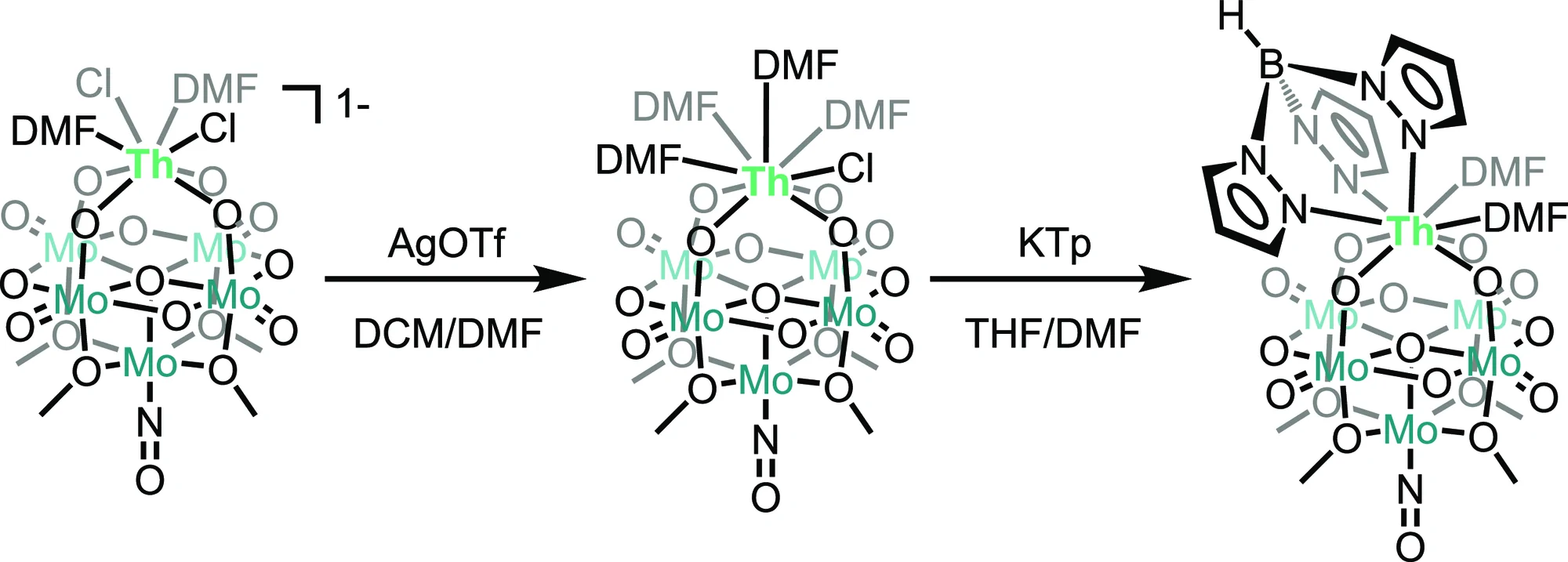
Synthetic Methods used to Synthesize **7-TpTh­(DMF)**_**2**_ **Mo**_**5**_ from **3-ThCl**_**2**_ **(DMF)**_**2**_ **Mo**_**5**_

To verify the formation of **6-ThCl­(DMF)**_**4**_ **Mo**_**5**_, single crystals were obtained by vapour diffusion of Et_2_O into a saturated solution of the complex dissolved in DCM and then were analysed using SCXRD. The structure obtained, after refinement of the data, is given in Figure 7. The structure contains two independent **6-ThCl­(DMF)**_**4**_ **Mo**_**5**_ complexes in the asymmetric unit. Each complex features a 9 coordinate Th center, showing that loss of a Cl anion mediates an increase in coordination number compared to **3-ThCl**_**2**_ **(DMF)**_**2**_ **Mo**_**5**_, expanding the range of accessible coordination geometries for An-POM complexes. The Th coordination geometry is close to ideal spherical capped square antiprismatic (CSAPR), with CShM values of 0.504 and 0.558 obtained for the two independent complexes in the asymmetric unit (Table S8). The Th–O_DMF_ bonds present in the structure vary significantly in length (2.365(4)-2.472(4) Å), though the global average Th–O_DMF_ bond length (2.43(3) Å) is close to that of **3-ThCl**_**2**_ **(DMF)**_**2**_ **Mo**_**5**_ (2.420(18) Å). The longest Th–O_DMF_ bond in each complex is the “vertical” Th1–O20 bond, which may be caused by the steric repulsion the DMF molecule faces when approaching the O18–O19–O21–Cl1 plane. Elongations in the average Th–Cl (2.81(3) Å) and Th-(O14-17) (2.481(11) Å) bond lengths compared to those of **3-ThCl**_**2**_ **(DMF)**_**2**_ **Mo**_**5**_ (2.739(8) Å and 2.437(5) Å respectively) are consistent with the increase in Th coordination number from 8 to 9.

**Figure 7:**
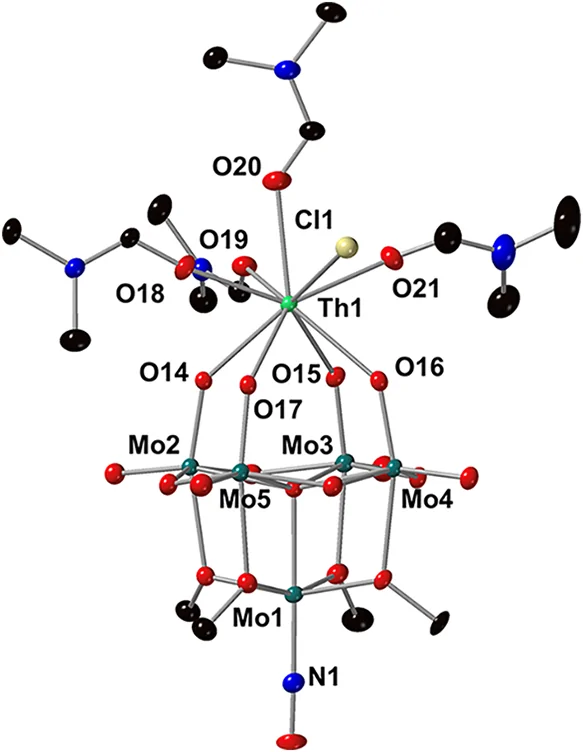
One of the two independent ThCl­(DMF)_4_Mo_5_O_13_(OMe)_4_NO complexes observed in the SCXRD structure of **6-ThCl­(DMF)**_**4**_ **Mo**_**5**_ with probability ellipsoids set at 50%.

After successful halide abstraction and isolation of **6-ThCl­(DMF)**_**4**_ **Mo**_**5**_, we turned our attention to reactivity toward KTp. Treatment of **6-ThCl­(DMF)**_**4**_ **Mo**_**5**_ with one equivalent of KTp in THF/DMF led to the formation of a slightly cloudy blue solution, consistent with salt metathesis and formation of insoluble KCl. The reaction mixture was filtered, concentrated and the crude product was precipitated via the addition of an excess of Et_2_O. The obtained solid was washed with more Et_2_O to ensure removal of free DMF, before extraction in DCM to separate the product from any remaining KCl. After drying and crystallization from DCM/Et_2_O, formation of the target Tp complex was confirmed by ^1^H NMR spectroscopy and ^17^O NMR spectroscopy. The ^1^H NMR spectrum (Figure S13) shows the expected resonances associated with the –OCH_3_ groups of the {Mo_5_} ligand (4.78 ppm) and DMF (2.87, 3.12, and 7.94), with integration supporting the presence of two DMF ligands per {Mo_5_} unit. Alongside these, there are a series of three pairs of signals between 6-8 ppm which are assigned to the aromatic protons of the Tp ligand. The peaks within these three pairs occur in a 2:1 ratio, suggesting that two of the three pyrazole rings of the Tp ligand are chemically equivalent. These results support retention of a 9-coordinate geometry at Th, with the chloride and two DMF ligands of **6-ThCl­(DMF)**_**4**_ **Mo**_**5**_ replaced by a single Tp ligand, giving TpTh­(DMF)_2_Mo_5_O_13_(OMe)_4_NO (**7-TpTh­(DMF)**_**2**_ **Mo**_**5**_).

This is further supported by the ^17^O NMR spectrum of **7-TpTh­(DMF)**_**2**_ **Mo**_**5**_ (Figure 8). The spectrum shows a total of 8 resonances which can be spilt into two resonances for the Mo=O nuclei (blue), three resonances for the Th–O–Mo nuclei (red), two resonances for the Mo–O–Mo nuclei (green), and a single resonance for the central μ_5_-O based on the chemical shifts. The number of resonances for each group of four oxygen nuclei in the structure (i.e., Mo=O, Th–O–Mo, and Mo–O–Mo) can be rationalized by considering the relative orientation of the plane created by the four nuclei in the group with respect to the mixed donor plane created by the ligands bound to the other face of Th (i.e., N^Tp^-N^Tp^-O^DMF^-O^DMF^). The planes created by the Mo=O and Th–O–Mo nuclei are twisted ca. 45° with respect to the ligand plane, leading to the presence of three unique ^17^O environments in a 1:2:1 ratio. These three resonances are resolved for the Th–O–Mo nuclei in **7-TpTh­(DMF)**_**2**_ **Mo**_**5**_, however only two resonances (3:1 ratio) are observed for the Mo=O nuclei. This is likely a result of small chemical shift differences between the unique environments, meaning the individual peaks overlap. The plane created by the Mo–O–Mo nuclei lies in line with the ligand plane (i.e., twist angle is ca. 0°) and thus only two unique environments are expected, one for the ^17^O nuclei lying directly below the N^Tp^ atoms (2 O) and one for the ^17^O nuclei lying below the O^DMF^ atoms (2 O). This is exactly what is observed in the ^17^O NMR spectrum of **7-TpTh­(DMF)**_**2**_ **Mo**_**5**_ (Figure 8). Thus, in this case, ^17^O NMR spectroscopy is not only a valuable tool for confirming the structure of the asymmetric complex but gives valuable insight into symmetry of the complex in solution. Unlike the ^17^O NMR spectrum of **4-ThCl**_**2**_ **(bipy)­Mo**_**5**_ (Figure 4), which suggests C_s_ symmetry is not retained in solution, the ^17^O NMR spectrum of **7-TpTh­(DMF)**_**2**_ **Mo**_**5**_ suggests strict retention of the Th coordination environment in solution as dynamic processes (e.g., rotation of the respective ligands) would lead to a simplified ^17^O NMR spectrum.

**Figure 8:**
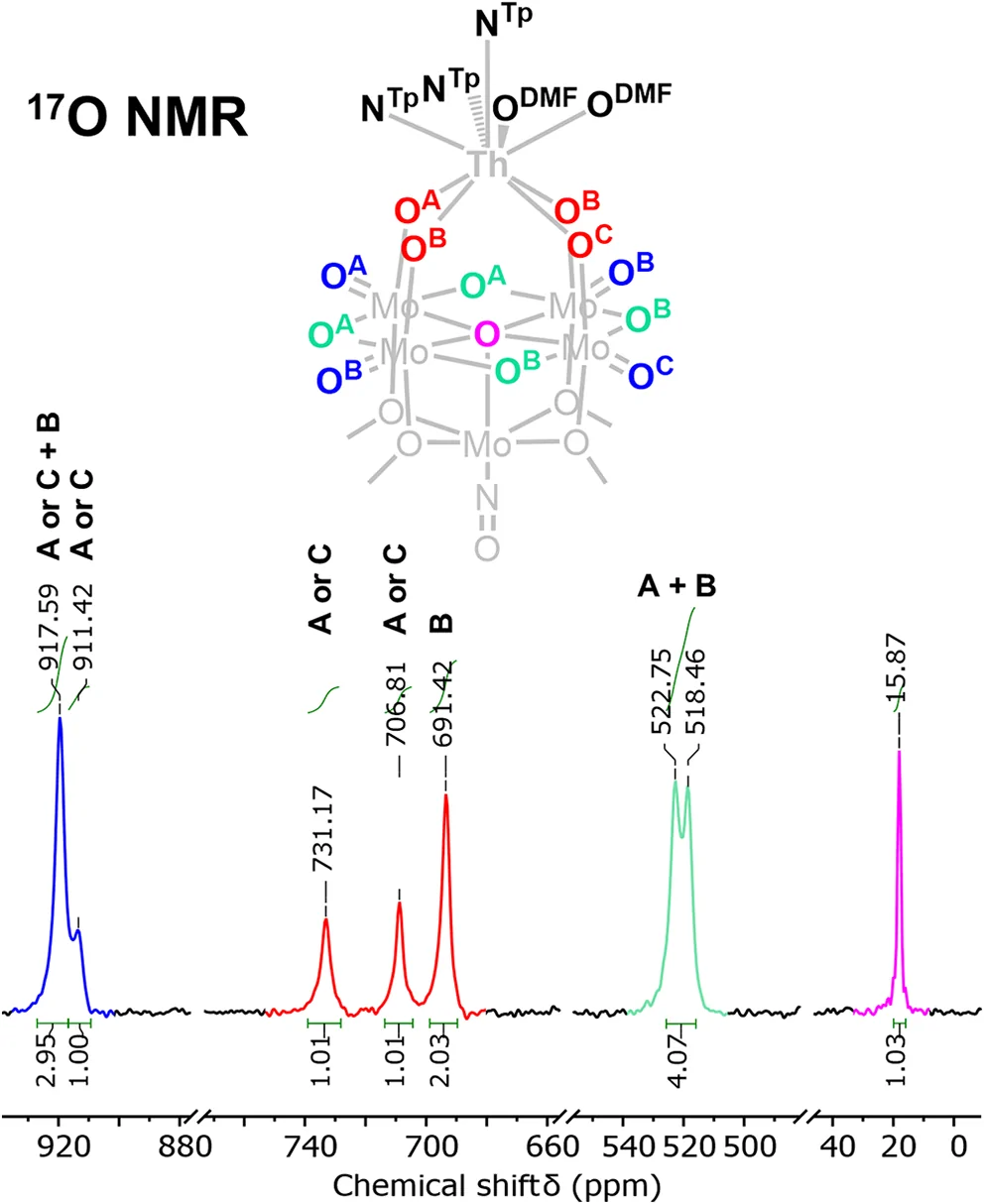
^17^O NMR spectrum of **7-TpTh­(DMF)**_**2**_ **Mo**_**5**_ recorded in CD_2_Cl_2_ at room temperature along with a graphical representation of the structure highlighting the unique ^17^O nuclei present in the complex.

This exact geometry was confirmed via SCXRD experiments. First, single crystals of **7-TpTh­(DMF)**_**2**_ **Mo**_**5**_ were obtained through vapor diffusion of Et_2_O into a saturated solution of the complex dissolved in DCM. Performing SCXRD with the obtained crystals gave, after refinement of the obtained data, the structure shown in Figure 9. The structure confirms the formation of an asymmetric complex featuring a single {Mo_5_} ligand and a single Tp ligand, with the coordination sphere of Th completed via the binding of two DMF molecules. This leads to retention of the 9-cooridnate geometry at Th, with CShM analysis indicating the geometry is well described as spherical capped square antiprismatic (CShM value = 0.402, Table S10). Tp binds in a such a manner that two of the three N-donor atoms form part of a N2–N4–O20-O21 plane (RMSD = 0.051 Å) which is completed by the O-donor atoms of the two DMF molecules. This places the third pyrazole ring on Tp directly above Th and therefore the N-donor atom of this ring occupies the “vertical” coordination site. This leads to a slightly longer Th–N6 bond (2.654(4) Å) length than the average Th1–N2/N4 bond distances (2.618(7) Å). This clearly illustrates the inequivalence between the respective pyrazole rings of Tp, and thus is consistent with the observed ^1^H NMR spectrum of **7-TpTh­(DMF)**_**2**_ **Mo**_**5**_, where pairs of peaks in a 2:1 ratio are observed for the pyrazole protons of Tp. The average Th–O_DMF_ (2.4283(16) Å) and Th–O­{Mo_5_} (2.440(16) Å) bond distances are marginally shorter than those in **6-ThCl­(DMF)**_**4**_ **Mo**_**5**_ suggesting a slightly stronger association between the Th and the O-donor ligands in the system after incorporation of Tp into the assembly.

**Figure 9:**
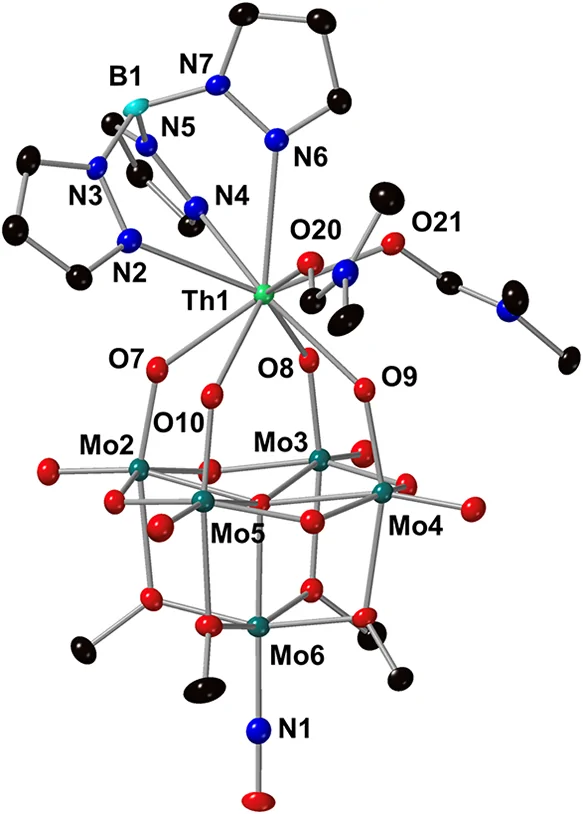
SCXRD structure of **7-TpTh­(DMF)**_**2**_ **Mo**_**5**_ with probability ellipsoids set at 50%. Hydrogen atoms, some disorder and solvent molecules have been removed for clarity.

### Electronic and Redox Properties of Asymmetric Complexes

With a series of asymmetric 1:1 Th-POM complexes in hand, we set out to verify how changes to the coordination environment of Th influence the electronic and redox properties of the overall assembly. To do this, we first performed UV-Vis spectroscopy on solutions of the complexes dissolved in DCM. DCM was chosen due to its ability to readily dissolve both the anionic and neutral complexes produced in this work. The obtained spectra are shown in Figure 10.

**Figure 10:**
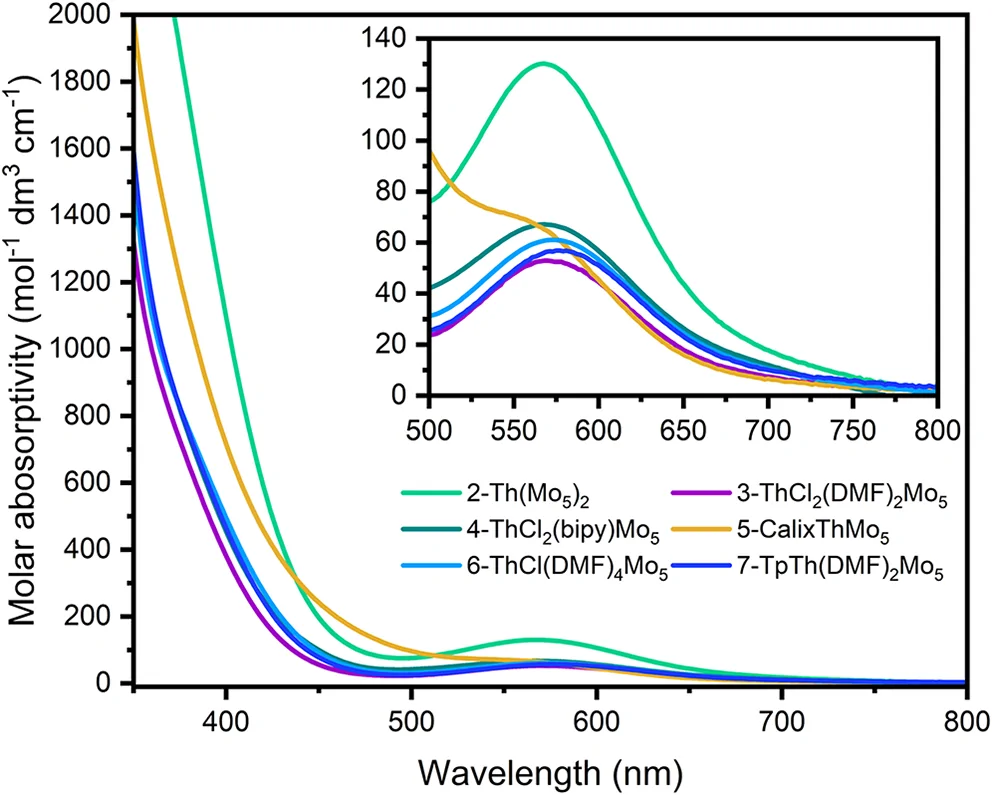
UV-Vis spectra of the asymmetric complexes prepared in this work compared to that of **2-Th­(Mo**_**5**_ **)**_**2**_. Spectra were recorded in DCM at 21 °C.

Qualitative examination of the spectra reveals that the majority of the asymmetric complexes possess a similar UV-Vis signature, with the primary feature being a low intensity transition at 555–575 nm. This indicates that changes to the Th coordination environment do not drive major changes in the optical properties of the assembly. Our previous work has shown that the absorption present at 568 nm (ε = 130 mol^–1^ dm^3^ cm^–1^) in the spectrum of **2-Th­(Mo**_**5**_ **)**_**2**_ is caused by transitions between filled (HOMO–3 through HOMO) and empty (LUMO+6 and LUMO+7) orbitals localized on the molybdenum nitrosyl unit (i.e., {Mo-NO}^4^).^55^ Thus, it is reasonable to assume that the broad absorption at 555-575 nm (ε = 53–70 mol^–1^ dm^3^ cm^–1^) in the spectra of the asymmetric complexes are caused by the same transitions. This is further supported by the fact that the intensity of this transition is approximately half that of the absorption observed at the same position in the spectrum of **2-Th­(Mo**_**5**_ **)**_**2**_ (i.e., the effective concentration of {Mo_5_} in solutions of the asymmetric complexes studied in this work is half of that in solutions of **2-Th­(Mo**_**5**_ **)**_**2**_ at the same molarity). Given these transitions are associated with orbitals which are localized at the periphery of the {Mo_5_} unit, it is unsurprising that this absorption feature appears insensitive to changes in the Th coordination environment. Interestingly, the spectrum of **2-Th­(Mo**_**5**_ **)**_**2**_ deviates significantly from those of the asymmetric complexes produced here at higher energies. A more intense absorption onsets at ca. 500 nm in the spectrum of **2-Th­(Mo**_**5**_ **)**_**2**_. This absorption is assigned to O­(2p) → Mo­(4d) ligand-to-metal charge transfer, with similar intense absorptions observed in the higher energy regions of POM systems.^97–100^ In the asymmetric complexes this absorption is less intense and onsets at slightly higher energy (ca. 450 nm). This behavior more closely resembles that of **1-NaMo**_**5**_ and thus could be caused by a lower effective concentration of {Mo_5_} in samples of asymmetric complexes compared to that of **2-Th­(Mo**_**5**_ **)**_**2**_ and/or a change in the energy of this process when moving from a sandwich-type complex to complexes containing a single {Mo_5_} ligand.

The spectrum of **5-** **CalixThMo**_**5**_ shows large deviations from the other asymmetric complexes studied. The same low intensity transition is observed at ca. 558 nm but it now appears as a shoulder on the side of another more intense, higher energy transition. Given the absence of this transition from the spectra of the other complexes studied, and that π → π* transitions for calix[4]­arenes are reported at higher energies (250–300 nm),^89,91^ it is likely that this transition is caused by charge transfer between low lying filled orbitals on the calix[4]­arene unit and empty Mo­(4d) orbitals that form the basis of the LUMO in {Mo_5_} containing systems.^55^ This process clearly occurs at lower energies than the O­(2p) → Mo­(4d) LMCT which causes the intense absorption that onsets ca. 400–450 nm in the other asymmetric {Mo_5_} containing systems. This presence of low-lying filled orbitals on the calix[4]­arene unit is supported by the fact calix[4]­arenes are known to oxidize are mild potentials to form phenoxy radicals (see reference and CV of **5-** **CalixThMo**_**5**_ discussed below).^91^

Finally, we employed CV to examine the redox properties of the series. In previous work, we showed that the CV of **2-Th­(Mo**_**5**_ **)**_**2**_ in MeCN (100 mM TBA­(PF_6_), 200 mv/s scan rate) displays four reversible reduction events, consistent with reversible two electron reduction of each {Mo_5_} unit. Similar behavior is observed in DCM (Figure S14), though the fourth reduction event appears slightly distorted which is likely due to its proximity to the edge of the electrochemical window available to DCM/TBA­(PF_6_).

The redox properties of the complexes studied in this work appear to be largely influenced by the presence of chloride anions within the structure. The CV of **3-ThCl**_**2**_ **(DMF)**_**2**_ **Mo**_**5**_ (Figure 11a) was recorded in DMF to attempt to suppress any irreversible behavior associated with DMF dissociation. The CV displays two irreversible reduction events at ca. −1.09 V and −1.47 V vs Fc^+/0^. Observation of fewer reduction events in the CV of **3-ThCl**_**2**_ **(DMF)**_**2**_ **Mo**_**5**_ when compared to that of **2-Th­(Mo**_**5**_ **)**_**2**_ is consistent with the fact that only one {Mo_5_} unit is available for reduction in **3-ThCl**_**2**_ **(DMF)**_**2**_ **Mo**_**5**_. The irreversibility of the redox events is indicative of a chemical process following electron transfer. In this case, it is likely that reduction is coupled with chloride dissociation, which could then lead to further DMF coordination or decomposition. Four low intensity features (i.e., oxidation events) are observed in the return scan. The first two events (ca. −1.36 V and −0.99 V vs Fc^+/0^) are attributed to the re-oxidation of small amounts of one and two electron reduced **3-ThCl**_**2**_ **(DMF)**_**2**_ **Mo**_**5**_ that persist in solution long enough to be oxidized without loss of chloride ions (or decomposition). The two additional oxidation events (−0.74 V and −0.43 V vs Fc^+/0^) were not observed in the first scan (Figure S15) suggesting they are associated with species produced when sweeping cathodically (i.e., the products of reduction and chloride dissociation). Similar behavior was observed when performing the CV of **3-ThCl**_**2**_ **(DMF)**_**2**_ **Mo**_**5**_ in DCM, though additional irreversible features are observed (Figure S16). In an attempt to improve the reversibility of the reduction events, we added increasing amounts of TBA­(Cl) to the system, hypothesizing that the presence of excess Cl^–^ would facilitate reversible chloride dissociation/binding to complex upon electron transfer (Figure 11a). However, no real improvement in reversibility was observed, suggesting either that the stability of the complexes produced after reduction is low or that the kinetics of Cl^–^ binding during oxidation is slow with respect to the time scale of a CV. We can also not rule out the possibility that the irreversibility is not caused by chloride dissociation.

**Figure 11:**
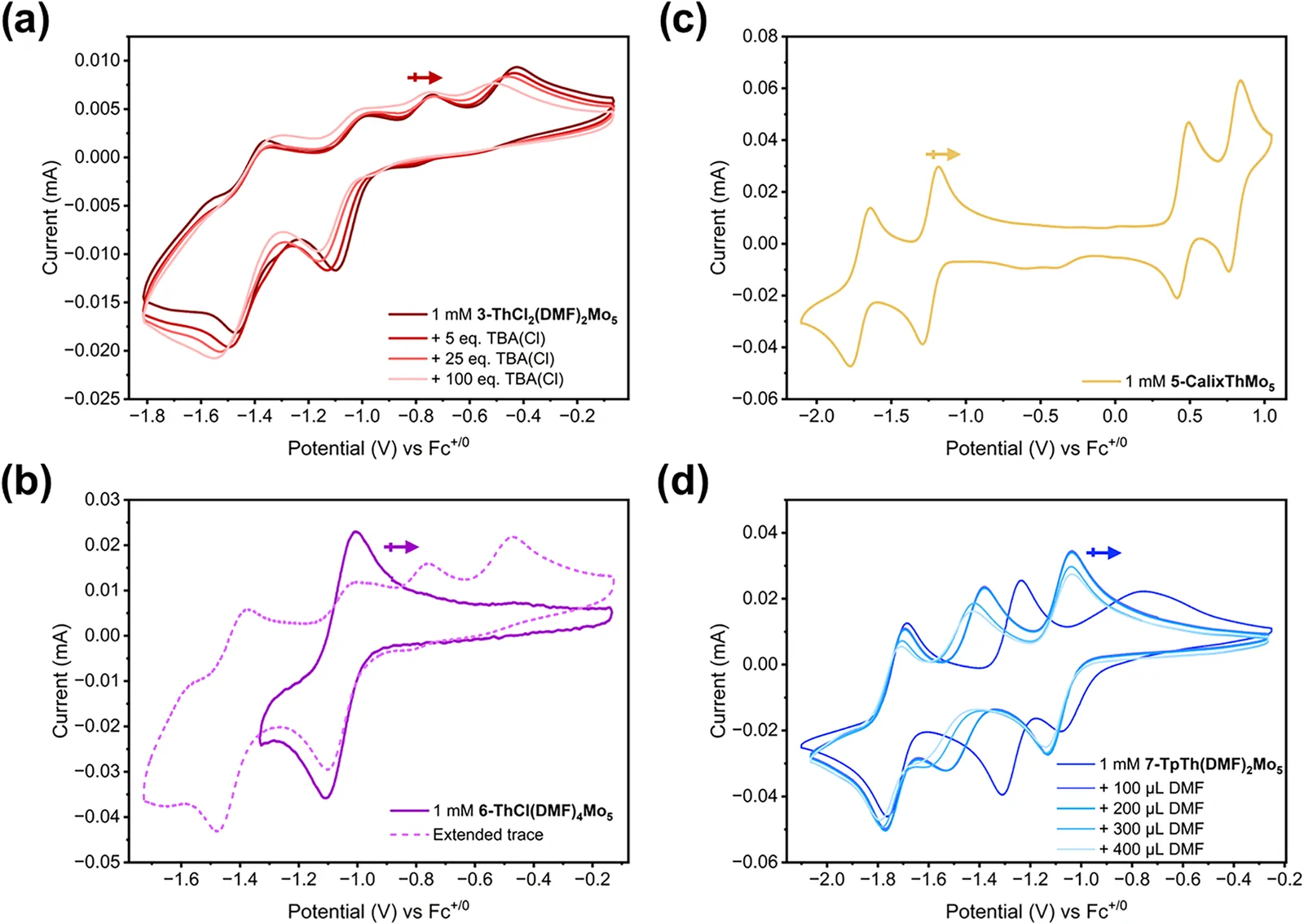
Cyclic voltammograms of 1 mM solutions of (a) **3-ThCl**_**2**_ **(DMF)**_**2**_ **Mo**_**5**_, (b) **6-ThCl­(DMF)**_**4**_ **Mo**_**5**_, (c) **5-CalixThMo**_**5**_, and (d) **7-TpTh­(DMF)**_**2**_ **Mo**_**5**_. CVs were recorded in the presence of 100 mM of TBA­(PF_6_) supporting electrolyte in either DMF (a and b) or DCM (c and d). CVs were all recorded at 21 °C.

The CV of **4-ThCl**_**2**_ **(bipy)­Mo**_**5**_ (Figure S17) is very similar to that of **3-ThCl**_**2**_ **(DMF)**_**2**_ **Mo**_**5**_, supporting the idea that chloride dissociation is contributing to the observed irreversible reduction events. However, the CV of **6-ThCl­(DMF)**_**4**_ **Mo**_**5**_ (Figure 11b) is slightly different. When limiting the scan range to ca. −1.3 V vs Fc^+/0^, the first reduction event is much more reversible. This behavior is not replicated in the CVs of **3-ThCl**_**2**_ **(DMF)**_**2**_ **Mo**_**5**_ or **4-ThCl**_**2**_ **(bipy)­Mo**_**5**_ (Figures S17 and S18) when isolating only the first redox event. This change in behavior can be rationalized by considering that the halide abstraction step used to produce **6-ThCl­(DMF)**_**4**_ **Mo**_**5**_ from **3-ThCl**_**2**_ **(DMF)**_**2**_ **Mo**_**5**_ could decouple the first reduction event from concomitant chloride dissociation. This means that rather than electron transfer being accompanied by a chemical process, purely electron transfer behavior is observed, as halide loss and reorganization was achieved during the synthesis of the complex. Scanning to more negative potentials (ca. −1.6 V vs Fc^+/0^) leads to an almost identical CV to that of **3-ThCl**_**2**_ **(DMF)**_**2**_ **Mo**_**5**_. This suggests that the second reduction event of **6-ThCl­(DMF)**_**4**_ **Mo**_**5**_ is coupled with a chemical process. Furthermore, the fact that the same four reoxidation events are observed in both CVs indicates that the same species (or mixture of species) is generated at the most reducing potentials, whether starting with **3-ThCl**_**2**_ **(DMF)**_**2**_ **Mo**_**5**_ or **6-ThCl­(DMF)**_**4**_ **Mo**_**5**_. This further supports halide dissociation being an important factor in the redox chemistry of these species.

If halide dissociation is a key factor in determining the reversibility of the reduction events, the CV of **5-CalixThMo**_**5**_, which possesses neither labile DMF or Cl^–^ ligands, should display reversible redox chemistry. This is exactly what is shown in Figure 11c, with two reversible reduction events observed at −1.24 V and −1.71 V vs Fc^+/0^, consistent with reversible reduction of the {Mo_5_} ligand. It is therefore clear that the presence of the multidentate {Calix} ligand stabilizes the reduced analogues of this complex with respect to reorganization and/or decomposition. The CV of **5-CalixThMo**_**5**_ also shows the presence of two pseudo reversible oxidation events (*E*_1/2_ = 0.45 and 0.80 V vs Fc^+/0^). These oxidation peaks have been previously observed in the CVs of (TBA)_2_[CalixLnMo_5_O_13_(OMe)_4_NO] (Ln = Nd and Er) and are attributed to oxidation of the {Calix} ligand.^91^ The small features observed between ca. −0.5 V and −0.75 V vs Fc^+/0^ are only observed when scanning past the oxidation events of **5-CalixThMo**_**5**_ (Figure S19), suggesting they are caused by minor decomposition which occurs when scanning past these pseudo reversible oxidation events.

The CV of **7-TpTh­(DMF)**_**2**_ **Mo**_**5**_ (Figure 11d) also shows the presence of two reversible reduction events at −1.27 V and −1.72 V vs Fc^+/0^, mirroring the behavior of **5-CalixThMo**_**5**_, again reinforcing the hypothesis that the presence of labile Cl^–^ is the determining factor in dictating the reversibility of reduction events in the asymmetric Th-POM complexes studied in this work. In addition to these two events, a third pseudo reversible reduction event (*E*_pc_ = −1.07 V; E_pa_ = −0.76 V vs Fc^+/0^) was consistently observed in all CVs. The shape of this peak is preserved even when this redox event is isolated (Figure S20). To attempt to ascertain whether this feature is caused by an impurity or a dynamic process, we explored addition of DMF to the solution. Adding small amounts (4 eq., Figure S21) led to no change. However, addition of excess DMF (100-400 μL) led to major shifts in the positions of the first two redox events (Figure 11d). After addition of 100 μL of DMF, a reversible reduction event is observed at −1.08 V vs Fc^+/0^ and pseudo reversible reduction event is present at −1.46 V vs Fc^+/0^. This large change would not be expected if the pseudo reversible redox event present in the initial CV was caused by an impurity. Instead, it is likely that reduction of **7-TpTh­(DMF)**_**2**_ **Mo**_**5**_ drives dynamic behavior involving the DMF ligands bound to the complex. The fact that both the position and reversibility of the first two events change as a function of the quantity of DMF present suggests both reduction events can involve either reversible electron transfer or electron transfer coupled to a chemical process depending on DMF concentration. Addition of more DMF (200-400 μL) only leads to a reduction in the reversibility of the second reduction process. Furthermore, recording CV **7-TpTh­(DMF)**_**2**_ **Mo**_**5**_ in DMF (Figure S22) only shows the presence of a pseudo reversible reduction event at −1.00 V vs Fc^+/0^ and an irreversible reduction event at −1.92 V vs Fc^+/0^, indicating a large excess of DMF is detrimental to the reversibility of the reduction chemistry. Interestingly, when scanning to highly negative potentials in DMF, the CV of **7-TpTh­(DMF)**_**2**_ **Mo**_**5**_ (Figure S22) starts to resemble that of **3-ThCl**_**2**_ **(DMF)**_**2**_ **Mo**_**5**_ (Figure 11a) and **6-ThCl­(DMF)**_**4**_ **Mo**_**5**_ (Figure 11b). This could indicate loss of the {Tp} ligand under these conditions, with [Th­(DMF)_*x*_Mo_5_O_13_(OMe)_4_NO]^1–^ being a common species at highly negative potentials in all CVs obtained in DMF.

## Conclusion

In summary, we have demonstrated that the use of strongly coordinating non-aqueous solvents provides a viable strategy for stabilizing and isolating a rare 1:1 An-POM complex. Treatment of either the lacunary precursor **1-NaMo**_**5**_ or the sandwich-type complex **2-Th­(Mo**_**5**_ **)**_**2**_ with ThCl_4_·2DME in DMF affords **3-ThCl**_**2**_ **(DMF)**_**2**_ **Mo**_**5**_, a thorium complex in which the coordination sphere is not saturated by POM ligands. Unlike previously reported aqueous systems that exist as dynamic equilibria of 1:1 and 1:2 species, **3-ThCl**_**2**_ **(DMF)**_**2**_ **Mo**_**5**_ is stable with respect to formation of the corresponding sandwich-type complex and therefore provides an isolable platform for further coordination chemistry studies. The presence of labile chloride and DMF ligands within the coordination sphere of thorium enables a range of subsequent transformations. Neutral ligand exchange with 2,2′-bipyridine, salt metathesis with a calix[4]­arene ligand, and halide abstraction followed by installation of tris­(pyrazolyl)­borate all proceed cleanly, providing access to a family of asymmetric Th-POM complexes. Structural characterization reveals that these transformations permit systematic modification of the thorium coordination environment, generating geometries that range from distorted square antiprismatic to biaugmented trigonal prismatic. These results establish **3-ThCl**_**2**_ **(DMF)**_**2**_ **Mo**_**5**_ as a versatile synthon for An-POM chemistry and demonstrate that non-aqueous approaches can provide access to coordination environments and reactivity patterns that are inaccessible in traditional aqueous An-POM systems. More broadly, this work opens new opportunities to investigate the coordination chemistry and reactivity of actinide centers supported by molecular metal-oxide ligands.

## Supplementary Material



## Notes

Crystallographic details, ^1^H and ^17^O NMR spectra of prepared compounds, and electrochemical data (PDF)

## References

[1] Grenthe, I. ; Drożdżynński, J. ; Fujino, T. ; Buck, E. C. ; Albrecht-Schmitt, T. E. ; Wolf, S. F. Uranium. In The Chemistry of the Actinide and Transactinide Elements; Morss, L. R. ; Edelstein, N. M. ; Fuger, J. , Eds.; Springer Netherlands, 2006; pp 253–698.

[2] Katz, J. J. ; Morss, L. R. ; Edelstein, N. M. ; Fuger, J. Introduction. In The Chemistry of the Actinide and Transactinide Elements; Morss, L. R. ; Edelstein, N. M. ; Fuger, J. , Eds.; Springer Netherlands, 2006; pp 1–17.

[3] Liddle S. T. (2015). The Renaissance
of Non-Aqueous Uranium Chemistry. Angew. Chem.,
Int. Ed..

[4] Nash, K. L. Chapter 121 Separation chemistry for lanthanides and trivalent actinides. In Handbook on the Physics and Chemistry of Rare Earths; Elsevier, 1994; Vol. 18, pp 197–238.

[5] Dam H. H., Reinhoudt D. N., Verboom W. (2007). Multicoordinate ligands
for actinide/lanthanide
separations. Chem. Soc. Rev..

[6] Veliscek-Carolan J. (2016). Separation
of actinides from spent nuclear fuel: A review. J. Hazard. Mater..

[7] Agarwal R., Sharma M. K. (2018). Selective Electrochemical Separation
and Recovery of
Uranium from Mixture of Uranium­(VI) and Lanthanide­(III) Ions in Aqueous
Medium. Inorg. Chem..

[8] Zhang H., Li A., Li K., Wang Z., Xu X., Wang Y., Sheridan M. V., Hu H.-S., Xu C., Alekseev E. V., Zhang Z., Yan P., Cao K., Chai Z., Albrecht-Schönzart T. E., Wang S. (2023). Ultrafiltration separation
of Am­(VI)-polyoxometalate from lanthanides. Nature.

[9] Wang J., Li Y., Yao R., Yang B., Qu W., Lu J., Wu Z., Li Y., Lu Z., Geng Z., Wang Z. (2025). Selective
Crystallization Separation of Uranium­(VI) Complexes from Lanthanides. Inorg. Chem..

[10] Gardner B. M., Liddle S. T. (2013). Small-Molecule Activation at Uranium­(III). Eur. J. Inorg. Chem..

[11] Jori N., Keener M., Rajeshkumar T., Scopelliti R., Maron L., Mazzanti M. (2023). Dinitrogen cleavage
by a dinuclear
uranium­(iii) complex. Chem. Sci..

[12] Zhou E., Ren W., Hou G., Zi G., Fang D.-C., Walter M. D. (2015). Small Molecule
Activation Mediated by a Thorium Terminal Imido Metallocene. Organometallics.

[13] Johnson K. R. D., Hayes P. G. (2013). Cyclometalative
C–H bond activation in rare
earth and actinide metal complexes. Chem. Soc.
Rev..

[14] La
Pierre H. S., Meyer K. (2014). Activation of Small Molecules by
Molecular Uranium Complexes. Prog. Inorg. Chem..

[15] Hsueh F.-C., Chen D., Rajeshkumar T., Scopelliti R., Maron L., Mazzanti M. (2024). Two-Electron Redox
Reactivity of
Thorium Supported by Redox-Active Tripodal Frameworks. Angew. Chem..

[16] Batrice R. J., Karmel I. S. R., Yardeni G., Eisen M. S. (2012). Actinide Elements
in Catalysis. Encycl. Inorg. Bioinorg. Chem..

[17] Liu H., Ghatak T., Eisen M. S. (2017). Organoactinides
in catalytic transformations:
scope, mechanisms and Quo Vadis. Chem. Commun..

[18] Leduc J., Frank M., Jürgensen L., Graf D., Raauf A., Mathur S. (2019). Chemistry of Actinide
Centers in Heterogeneous Catalytic
Transformations of Small Molecules. ACS Catal..

[19] Batov M. S., Partlow H. T., Chatelain L., Seed J. A., Scopelliti R., Zivkovic I., Adams R. W., Liddle S. T., Mazzanti M. (2025). Catalytic
and stoichiometric stepwise conversion of side-on bound dinitrogen
to ammonia mediated by a uranium complex. Nat.
Chem..

[20] Riedhammer J., Halter D. P., Meyer K. (2023). Nonaqueous Electrochemistry
of Uranium
Complexes: A Guide to Structure–Reactivity Tuning. Chem. Rev..

[21] Keener M., Shivaraam R. A. K., Rajeshkumar T., Tricoire M., Scopelliti R., Zivkovic I., Chauvin A.-S., Maron L., Mazzanti M. (2023). Multielectron
Redox Chemistry of Uranium by Accessing the +II Oxidation State and
Enabling Reduction to a U­(I) Synthon. J. Am.
Chem. Soc..

[22] Pang Y., Rajeshkumar T., Scopelliti R., Maron L., Mazzanti M. (2025). C–C
bond cleavage and carbonylation enabled by an NNN-pincer uranium scaffold
via metal–arene interaction. Chem. Sci..

[23] Halter D.
P., Heinemann F. W., Bachmann J., Meyer K. (2016). Uranium-mediated electrocatalytic
dihydrogen production from water. Nature.

[24] Kestel B., Pividori D., Chowdhury S. R., Heinemann F. W., Vlaisavljevich B., Meyer K. (2025). Inverse Trans Influence and Uranium-Arene
σ-Bonding Drive Molecular Geometry: Ligand Modification from
Hard to Soft Flips the Oxide from Axial to Equatorial. J. Am. Chem. Soc..

[25] Gardner B. M., Liddle S. T. (2015). Uranium triamidoamine
chemistry. Chem. Commun..

[26] Hastings A. M., Colliard I., Kaseman D. C., Colla C. A., Deblonde G. J. P. (2025). Breaking
the Neptunyl Barrier: Direct Access to Neptunium­(IV) in Aqueous Solution
via Polyoxometalate-Mediated Reduction and Stabilization. Inorg. Chem..

[27] Colliard I., Deblonde G. J. P. (2024). From +I to +IV,
Alkalis to Actinides: Capturing Cations
across the Periodic Table with Keggin Polyoxometalate Ligands. Inorg. Chem..

[28] Colliard I., Stavila V., Deblonde G. J. P. (2025). Isolation
of a new polyoxometalate
complex of plutonium. Chem. Commun..

[29] Colliard I., Deblonde G. J. P. (2026). Actinide complexes
with Wells–Dawson polyoxometalates
(part 2): californium. Chem. Commun..

[30] Colliard I., Deblonde G. J. P. (2025). Isolation and characterization of a new polyoxometalate
ligand, H_3_SbW_14_O_50_
^10–^, and its interactions with f-elements. Chem.
Commun..

[31] Colliard I., Deblonde G. J. P. (2024). Characterization of the first Peacock–Weakley
polyoxometalate containing a transplutonium element: curium bis-pentatungstate
[Cm­(W_5_O_18_)_2_]^9–^. Chem. Commun..

[32] Colla C. A., Colliard I., Sawvel A. M., Nyman M., Mason H. E., Deblonde G. J. P. (2023). Contrasting Trivalent
Lanthanide and Actinide Complexation
by Polyoxometalates via Solution-State NMR. Inorg. Chem..

[33] Colliard I., Lee J. R. I., Colla C. A., Mason H. E., Sawvel A. M., Zavarin M., Nyman M., Deblonde G. J. P. (2022). Polyoxometalates
as ligands to synthesize, isolate and characterize compounds of rare
isotopes on the microgram scale. Nat. Chem..

[34] Colliard I., Deblonde G. J. P. (2026). Actinide complexes with Wells–Dawson polyoxometalates
(part 1): americium and curium. Chem. Commun..

[35] Colliard I., Deblonde G. J. P. (2024). Polyoxometalate Ligands Reveal Different Coordination
Chemistries Among Lanthanides and Heavy Actinides. JACS Au.

[36] Bustos J., Colliard I., Stavila V., Kaseman D. C., Colla C. A., Nyman M., Deblonde G. J. P. (2026). Structural
and Spectroscopic Characterization
of Plutonium and Other Tetravalent Metals Complexed to a Keggin Ion. Inorg. Chem..

[37] Subintoro P. J., Carter K. P. (2025). Delineating the
Effects of Counterions on the Structural
and Vibrational Properties of U­(IV) Lindqvist Polyoxometalate Complexes. Inorg. Chem..

[38] Dufaye M., Duval S., Loiseau T. (2020). Trends and
new directions in the
crystal chemistry of actinide oxo-clusters incorporated in polyoxometalates. CrystEngComm.

[39] Pope, M. T. Chapter 9 - Structural chemistry of actinide polyoxometalates. In Structural Chemistry of Inorganic Actinide Compounds; Krivovichev, S. V. ; Burns, P. C. ; Tananaev, I. G. , Eds.; Elsevier, 2007; pp 341–361.

[40] Auvray T., Matson E. M. (2020). Polyoxometalate-based complexes as ligands for the
study of actinide chemistry. Dalton Trans..

[41] Williams C.
W., Antonio M. R., Soderholm L. (2000). The formation and stability of [EuP_5_W_30_O_110_]^12–^ and [AmP_5_W_30_O_110_]^12–^. J. Alloys Compd..

[42] Antonio M. R., Chiang M.-H. (2008). Stabilization of Plutonium­(III) in
the Preyssler Polyoxometalate. Inorg. Chem..

[43] Antonio M.
R., Williams C. W., Soderholm L. (1998). Synthesis and characterization of
actinide-exchanged Preyssler heteropolyanions [AnP_5_W_30_O_110_]^n–^ (An = Th, Am, Cm). J. Alloys Compd..

[44] Molchanov V. N., Tatjanina I. V., Torchenkova E. A., Kazansky L. P. (1981). A novel type of
heteropolynuclear complex anion: X-ray crystal structure of the polymeric
complex anion [Th­(H_2_O)_3_UMo_12_O_42_]^4–^. J. Chem. Soc.,
Chem. Commun..

[45] Yang G.-P., Li K., Hu C.-W. (2022). Recent advances in uranium-containing polyoxometalates. Inorg. Chem. Front..

[46] Copping R., Gaunt A. J., May I., Sharrad C. A., Collison D., Helliwell M., Fox O. D., Jones C. J. (2006). Oxoneptunium­(v)
as part of the framework of a polyoxometalate. Chem. Commun..

[47] Berg J. M., Gaunt A. J., May I., Pugmire A. L., Reilly S. D., Scott B. L., Wilkerson M. P. (2015). Unexpected
Actinyl Cation-Directed
Structural Variation in Neptunyl­(VI) A-Type Tri-lacunary Heteropolyoxotungstate
Complexes. Inorg. Chem..

[48] Talbot-Eeckelaers C., Pope S. J. A., Hynes A. J., Copping R., Jones C. J., Taylor R. J., Faulkner S., Sykes D., Livens F. R., May I. (2007). Luminescence from Neptunyl­(VI)
Species in Solution. J. Am. Chem. Soc..

[49] Xiong M., Wang X., Ji S., Liu Y., Zhao H., Gong Z., Wang H., Niu J., Zhang D. (2026). A Tetranuclear
Thorium-Containing Hexameric Antimonotungstate: Synthesis, Structure,
and Catalysis of the Knoevenagel Condensation. Inorg. Chem..

[50] Termes S. C., Pope M. T. (1978). Stabilization of uranium­(IV) in heteropoly
anions*. Transition Met. Chem..

[51] Boyd, H. J. ; Walters, A. D. ; Deblonde, G. J. P. ; Nyman, M. When Counterions Fall into Place: Alkali Effects on f-Element Phosphomolybdate Crystallization and Speciation ChemRxiv 2026 10.26434/chemrxiv.15002689/v2.

[52] Zhang C., Howell R. C., Scotland K. B., Perez F. G., Todaro L., Francesconi L. C. (2004). Aqueous Speciation Studies of Europium­(III) Phosphotungstate. Inorg. Chem..

[53] Zhang C., Bensaid L., McGregor D., Fang X., Howell R. C., Burton-Pye B., Luo Q., Todaro L., Francesconi L. C. (2006). Influence
of the Lanthanide Ion and Solution Conditions on Formation of Lanthanide
Wells–Dawson Polyoxotungstates. J. Cluster
Sci..

[54] Colliard I., Deblonde G. J. P. (2025). Similar but Different: Structural
and Spectroscopic
Characterization of Series of Neodymium, Europium, Americium, and
Curium Coordination Complexes. J. Am. Chem.
Soc..

[55] Shiels D., Berlfein A. C., Peluzo B. M. T. C., Lopez L. M., Mitchell A. W., Brennessel W. W., Zeller M., Crawley M. R., Bart S. C., Ruggiero M. T., Matson E. M. (2025). Probing the Framework Metal Dependent
Properties of Actinide-Centered Polyoxoalkoxide Sandwich-Type Complexes. Inorg. Chem..

[56] Shiels D., Brennessel W. W., Crawley M. R., Matson E. M. (2024). Leveraging a reduced
polyoxomolybdate-alkoxide cluster for the formation of a stable U­(v)
sandwich complex. Chem. Sci..

[57] Valerio L. R., Shiels D., Lopez L. M., Mitchell A. W., Zeller M., Bart S. C., Matson E. M. (2024). Venturing
Past Uranium: Synthesis
of a Np­(IV) Polyoxomolybdate–Alkoxide Sandwich Complex. Inorg. Chem..

[58] Klemperer W. G. (1990). Tetrabutylammonium
Isopolyoxometalates. Inorg. Synth..

[59] Proust A., Gouzerh P., Robert F. (1993). Molybdenum
oxo nitrosyl complexes.
1. Defect Lindqvist compounds of the type [Mo_5_O_13_(OR)_4_(NO)]^3‑^ (R = CH_3_, C_2_H_5_). Solid-state interactions with alkali-metal
cations. Inorg. Chem..

[60] Cantat T., Scott B. L., Kiplinger J. L. (2010). Convenient
access to the anhydrous
thorium tetrachloride complexes ThCl_4_(DME)_2_,
ThCl_4_(1,4-dioxane)_2_ and ThCl_4_(THF)_3.5_ using commercially available and inexpensive starting materials. Chem. Commun..

[61] Guillemot G., Solari E., Rizzoli C., Floriani C. (2002). Alkali and Alkaline-Earth-Metalated
Forms of Calix[4]­arenes: Synthons in the Synthesis of Transition Metal
Complexes. Chem. - Eur. J..

[62] CrysAlisPro, Rigaku Corporation 2024.

[63] Sheldrick G. (2015). SHELXT-Integrated
space-group and crystal-structure determination. Acta Crystallogr., Sect. A:Found. Adv..

[64] Sheldrick G. M. (2015). Crystal
structure refinement with SHELXL. Acta Crystallogr.,
Sect. C:Struct. Chem..

[65] Kepert D. L., Patrick J. M., White A. H. (1983). Structure
and stereochemistry in
f-block complexes of high co-ordination number. Part 1. The [M­(unidentate
ligand X)­(unidentate ligand Y)­7] system: crystal structure of bis­[chloroheptakis­(dimethylformamide)­uranium­(IV)]
tris­[tetrachlorodioxouranate­(VI)]. J. Chem.
Soc., Dalton Trans..

[66] Barton R. J., Dabeka R. W., Shengzhi H., Mihichuk L. M., Pizzey M., Robertson B. E., Wallace W. J. (1983). (N,N-Dimethylformamide)­tetrakis­(8-quinolinolato)­thorium­(IV),
[Th­(C_3_H_7_NO)­(C_9_H_6_NO)_4_]. Acta Crystallogr., Sect. C: Cryst.
Struct. Commun..

[67] Xu J., Raymond K. N. (2006). Structurally Characterized Quadruple-Stranded Bisbidentate
Helicates. Angew. Chem., Int. Ed..

[68] Travadi M., Jadeja D. R. N., Butcher R. J. (2024). Covalency parameters for nine-coordinated
thorium acylpyrazolone complexes along with their synthesis and crystal
features. J. Mol. Struct..

[69] Casellato U., Sitran S., Tamburini S., Vigato P. A., Graziani R. (1984). Lanthanide
and actinide complexes with bidentate ligands. Crystal structure of
dimethylformamidetetrakis­(1-oxo-2-thiopyridinato) thorium­(IV). Inorg. Chim. Acta.

[70] Berthet J.-C., Thuéry P., Ephritikhine M. (2005). New Efficient Synthesis of [UI_4_(MeCN)_4_]. X-ray Crystal Structures of [UI_2_(MeCN)_7_]­[UI_6_], [UI_4_(py)_3_], and [U­(dmf)_9_]­I_4_. Inorg.
Chem..

[71] Martin N. P., Volkringer C., Henry N., Trivelli X., Stoclet G., Ikeda-Ohno A., Loiseau T. (2018). Formation of a new type of uranium­(iv)
poly-oxo cluster {U38} based on a controlled release of water via
esterification reaction. Chem. Sci..

[72] Salmon L., Thuéry P., Ephritikhine M. (2006). Uranium­(iv) complexes of calix­[n]­arenes
(n = 4, 6 and 8). Chem. Commun..

[73] Salmon L., Thuéry P., Asfari Z., Ephritikhine M. (2006). Synthesis,
crystal structure and reactivity of uranium­(iv) complexes with p-tert-butylcalix[4]­arene
ligands. Dalton Trans..

[74] Baudry D., Ephritikhine M., Klaeui W., Lance M., Nierlich M., Vigner J. (1991). Uranium­(IV)
complexes with the oxygen tripod ligand
L- = CpCo­[P­(O)­(OEt)_2_]_3_
^‑^. X-ray
crystal structure of L_2_UCl_2_. Inorg. Chem..

[75] Carpenter S. H., Klamm B. E., Fetrow T. V., Scott B. L., Gaunt A. J., Anderson N. H., Tondreau A. M. (2023). Chlorination
of Pu and U Metal Using
GaCl_3_. Inorg. Chem..

[76] Pinsky M., Avnir D. (1998). Continuous Symmetry Measures. 5. The Classical Polyhedra. Inorg. Chem..

[77] Cirera J., Ruiz E., Alvarez S. (2005). Continuous
Shape Measures as a Stereochemical
Tool in Organometallic Chemistry. Organometallics.

[78] Shiels D., Brennessel W. W., Matson E. M. (2026). Tuning Uranium Redox Chemistry in
Asymmetric Polyoxometalate Complexes: Access to U­(IV), U­(V), and Transient
U­(VI). Inorg. Chem..

[79] Shiels D., Gunarathna N., Brennessel W. W., Matson E. M. (2026). Lanthanide-Centered
Polyoxoalkoxide Complexes Provide a Platform for Systematic Room-
and Variable-Temperature Nuclear Magnetic Resonance Studies. Inorg. Chem..

[80] Shiels D., Pittalis M., Gunarathna N., Berlfein A. C., Brennessel W. W., Ruggiero M. T., Matson E. M. (2025). Isolation
of Ce­(IV) centered polyoxoalkoxide
sandwich-type complexes allows comparison of metal–oxygen bond
covalency. Chem. Sci..

[81] Llunell, M. ; Casanova, D. ; Cirera, J. ; Alemany, P. ; Alvarez, S. SHAPE, version 2.1, Universitat de Barcelona 2013.

[82] Jeannin Y. (2005). Synthèse
et étude cristallographique d’un nouveau composé
de coordination asymétrique de l’uranium­(IV) lié
à deux ligands du type polytungstate [(H_3_Sb^III^W_17_O_59_)­U^IV^(HW_5_O_18_)]^11–^. C. R.
Chim..

[83] Sokolova M. N., Andreev G. B., Yusov A. B. (2011). First transuranium
mixed-ligand polyoxometalate
complex. Inorg. Chem. Commun..

[84] Subintoro P. J., Carter K. P. (2024). Structural and vibrational properties of lanthanide
Lindqvist polyoxometalate complexes. Dalton
Trans..

[85] Shiddiq M., Komijani D., Duan Y., Gaita-Ariño A., Coronado E., Hill S. (2016). Enhancing coherence in molecular
spin qubits via atomic clock transitions. Nature.

[86] Rupasinghe D. M. R. Y. P., Baxter M. R., Gupta H., Poore A. T., Higgins R. F., Zeller M., Tian S., Schelter E. J., Bart S. C. (2022). Actinide–Oxygen
Multiple Bonds from Air: Synthesis and Characterization of a Thorium
Oxo Supported by Redox-Active Ligands. J. Am.
Chem. Soc..

[87] Ringgold M., Wu W., Stuber M., Kornienko A. Y., Emge T. J., Brennan J. G. (2018). Monomeric
thorium chalcogenolates with bipyridine and terpyridine ligands. Dalton Trans..

[88] Mora E., Maria L., Biswas B., Camp C., Santos I. C., Pécaut J., Cruz A., Carretas J. M., Marçalo J., Mazzanti M. (2013). Diamine Bis­(phenolate) as Supporting Ligands in Organoactinide­(IV)
Chemistry. Synthesis, Structural Characterization, and Reactivity
of Stable Dialkyl Derivatives. Organometallics.

[89] Jiao Y., Sanz S., Izarova N. V., van Leusen J., Sarwar S., Dalgarno S. J., Brechin E. K., Kögerler P. (2022). Hybrid lanthanide
double-deckers based on calixarene and polyoxometalate units. Dalton Trans..

[90] Jiao Y., Sarwar S., Sanz S., van Leusen J., Izarova N. V., Campbell C. L., Brechin E. K., Dalgarno S. J., Kögerler P. (2021). Exploiting complementary ligands
for the construction
of square antiprismatic monometallic lanthanide SMMs. Dalton Trans..

[91] Jiao Y., Sanz S., Koláčná L., van Leusen J., Izarova N. V., Sarwar S., Ludvík J., Kögerler P. (2024). Coordination-triggered redox activity of early and
late lanthanide calix[4]­arene complexes. Dalton
Trans..

[92] Khalil K., Fiser B., Małecka M. (2025). Computational insights into solvent
encapsulation and host–guest recognition by calix[4]­arene. Sci. Rep..

[93] Shimizu K. D., Rebek J. (1995). Synthesis and assembly of self-complementary calix[4]­arenes. Proc. Natl. Acad. Sci. U.S.A..

[94] Gassoumi B., Ghalla H., Chaabane R. B. (2020). Host-guest
complexation studies of
NO_3_, NO_2_, CO_2_, and N_2_ gas
with the calix[4]­arene molecule. J. Mol. Model..

[95] Old J., Danopoulos A. A., Winston S. (2003). Uranium complexes with dianionic
O-methylated calix[4]­arene ligands. New J. Chem..

[96] Jantz S. G., Erdmann R., Hariyani S., Brgoch J., Höppe H. A. (2020). Sr_6_(BO_3_)_3_BN_2_: An Oxido–Nitrido–Borate
Phosphor Featuring BN2 Dumbbells. Chem. Mater..

[97] Kyle J. H. (1983). Kinetics
of the base decomposition of dodecatungstophosphate(3−) in
weakly alkaline solutions. J. Chem. Soc., Dalton
Trans..

[98] Nomiya K., Sugie Y., Amimoto K., Miwa M. (1987). Charge-transfer absorption
spectra of some tungsten­(VI) and molybdenum­(VI) polyoxoanions. Polyhedron.

[99] Ravelli D., Dondi D., Fagnoni M., Albini A., Bagno A. (2011). Predicting
the UV spectrum of polyoxometalates by TD-DFT. J. Comput. Chem..

[100] Ravelli D., Dondi D., Fagnoni M., Albini A., Bagno A. (2013). Electronic and EPR spectra of the
species involved in [W_10_O_32_]^4–^ photocatalysis. A relativistic
DFT investigation. Phys. Chem. Chem. Phys..

