# Supplementary material for: Coordination Chemistry of Thorium Supported by a Single Polyoxoalkoxide Ligand
Source: Inorg Chem. 2026 Sep 12;65(38):22505–20. doi: 10.1021/acs.inorgchem.6c03105 (PMC13625971; doi:10.1021/acs.inorgchem.6c03105)
Supplement: Supplementary file 1 [file ic6c03105_si_001.pdf]

Electronic Supporting Information

Coordination Chemistry of Thorium Supported by a Single Polyoxoalkoxide Ligand

Dominic Shiels[1]*, Adriana C. Berlfein[1], William W. Brennessel[1], and Ellen M. Matson[1]*

[1]Department of Chemistry, University of Rochester, Rochester NY 14627 USA

Corresponding Author Contact Information:

Dominic Shiels: dshiels@ur.rochester.edu

Ellen M. Matson: matson@chem.rochester.edu

Contents

Figure S1. ^{17}O NMR spectrum (67.8 MHz) of 2-Th(Mo_5)$_2$ in DMF. Spectrum acquired at 21 °C. S4

Figure S2. ^{17}O NMR spectrum (67.8 MHz) obtained during the reaction of 1-NaMo_5 with $ThCl_4$•2DME in DMF. Spectrum acquired at 21 °C. S4

Figure S3. ^{1}H NMR spectrum (500 MHz) of 3-$ThCl_2$(DMF)$_2$$Mo_5$ in CD_3CN. Spectrum acquired at 21 °C. S5

Figure S4. ^{17}O NMR spectrum (67.8 MHz) of 3-$ThCl_2$(DMF)$_2$$Mo_5$ in CD_3CN. Spectrum acquired at 21 °C. S5

Figure S5. ^{17}O NMR spectrum (67.8 MHz) obtained during the reaction of 2-Th(Mo_5)$_2$ with $ThCl_4$•2DME in DMF. Spectrum acquired at 21 °C. S6

Figure S6. ^{1}H NMR spectrum (500 MHz) of 3-$ThCl_2$(DMF)$_2$$Mo_5$ obtained from the reaction of 2-Th(Mo_5)$_2$ with $ThCl_4$•2DME in DMF. Spectrum acquired in CD_2Cl_2 at 21 °C. S6

Figure S7. ^{17}O NMR spectrum (67.8 MHz) of 3-$ThCl_2$(DMF)$_2$$Mo_5$ obtained from the reaction of 2-Th(Mo_5)$_2$ with $ThCl_4$•2DME in DMF. Spectrum acquired in CD_2Cl_2 at 21 °C S7

Figure S8. ^{1}H NMR spectrum (500 MHz) of 3-$ThCl_2$(DMF)$_2$$Mo_5$ inTHF-d_8. Spectrum acquired at 21 °C. S7

Figure S9. ^{1}H NMR spectrum (500 MHz) of 5-CalixThMo_5 in CD_2Cl_2. Spectrum acquired at 21 °C. S8

Figure S10. ^{17}O NMR spectrum (67.8 MHz) of 5-CalixThMo_5 in CD_2Cl_2. Spectrum acquired at 21 °C..S8

Figure S11. ^{1}H NMR spectrum (500 MHz) of 6-$ThCl$(DMF)$_4$$Mo_5$ in CD_2Cl_2. Spectrum acquired at 21 °C. S9

Figure S12. ^{17}O NMR spectrum (67.8 MHz) of 6-$ThCl$(DMF)$_4$$Mo_5$ in CD_2Cl_2. Spectrum acquired at 21 °C. Minor peaks are 536.46 and 717.76 ppm are attributed to 3-$ThCl_2$(DMF)$_2$$Mo_5$. S9

Figure S13. ^{1}H NMR spectrum (500 MHz) of 7-TpTh(DMF)$_2$$Mo_5$ in CD_2Cl_2. Spectrum acquired at 21 °C. S10

Figure S14. CV of 2-Th(Mo_5)$_2$ (1 mM) recorded in DCM with 100 mM of TBA(PF_6) supporting electrolyte. The CV was acquired at a scan rate of 200 mv/s at 21 °C. S10

Figure S15. 1st scan of the CV of 3-$ThCl_2$(DMF)$_2$$Mo_5$ (1 mM) recorded in DMF with 100 mM of TBA(PF_6) supporting electrolyte. The CV was acquired at a scan rate of 200 mv/s at 21 °C. S11

Figure S16. CV of 3-$ThCl_2$(DMF)$_2$$Mo_5$ (1 mM) recorded in DCM with 100 mM of TBA(PF_6) supporting electrolyte. The CV was acquired at a scan rate of 200 mv/s at 21 °C. S11

Figure S17. CV of 4-$ThCl_2$(bipy)Mo_5 (1 mM) recorded in DCM with 100 mM of TBA(PF_6) supporting electrolyte. The CV was acquired at a scan rate of 200 mv/s at 21 °C. S12

Figure S18. CV of 3-$ThCl_2$(DMF)$_2$$Mo_5$ (1 mM) with the first wave isolated. The CV was recorded in DMF with 100 mM of TBA(PF_6) supporting electrolyte, a scan rate of 200 mv/s and at 21 °C. S12

Figure S19. CV highlighting the reductive events of 5-CalixThMo_5 (1 mM) recorded in DCM with 100 mM of TBA(PF_6) supporting electrolyte. The CV was acquired at a scan rate of 200 mv/s at 21 °C. ..S13

Figure S20. CV of 7-TpTh(DMF)$_2$$Mo_5$ (1 mM) with the first wave isolated. The CV was recorded in DCM with 100 mM of TBA(PF_6) supporting electrolyte, a scan rate of 200 mv/s and at 21 °C. S13

Figure S21. CV of 7-TpTh(DMF)$_2$$Mo_5$ (1 mM) with 4 eq. of DMF. The CV was recorded in DCM with 100 mM of TBA(PF_6) supporting electrolyte, a scan rate of 200 mv/s and at 21 °C. S14

Figure S22. CV of 7-TpTh(DMF)$_2$$Mo_5$ (1 mM) recorded in DMF with 100 mM of TBA(PF_6) supporting electrolyte. The CV was acquired at a scan rate of 200 mv/s at 21 °C. S14

Table S1: Crystal data and structure refinement for 3-$ThCl_2$(DMF)$_2$$Mo_5$....................S15

Table S2: Continuous Shape Measures (CShM) analysis for 3-$ThCl_2(DMF)_2Mo_5$. Positions in Cartesian coordinates (x,y,z). S16

Table S3: Crystal data and structure refinement for 4-$ThCl_2(bipy)Mo_5$ S19

Table S4: Continuous Shape Measures (CShM) analysis for 4-$ThCl_2(bipy)Mo_5$. Positions in Cartesian coordinates (x,y,z). S20

Table S5: Crystal data and structure refinement for 5-$CalixThMo_5$ S23

Table S6: Continuous Shape Measures (CShM) analysis for 5-$CalixThMo_5$. Positions in Cartesian coordinates (x,y,z). S24

Table S7: Crystal data and structure refinement for 6-$ThCl(DMF)_4Mo_5$ S27

Table S8: Continuous Shape Measures (CShM) analysis for the two unique complexes in the structure of 6-$ThCl(DMF)_4Mo_5$. Positions in Cartesian coordinates (x,y,z). S28

Table S9: Crystal data and structure refinement for 7-$TpTh(DMF)_2Mo_5$ S35

Table S10: Continuous Shape Measures (CShM) analysis for 7-$TpTh(DMF)_2Mo_5$. Positions in Cartesian coordinates (x,y,z). S36

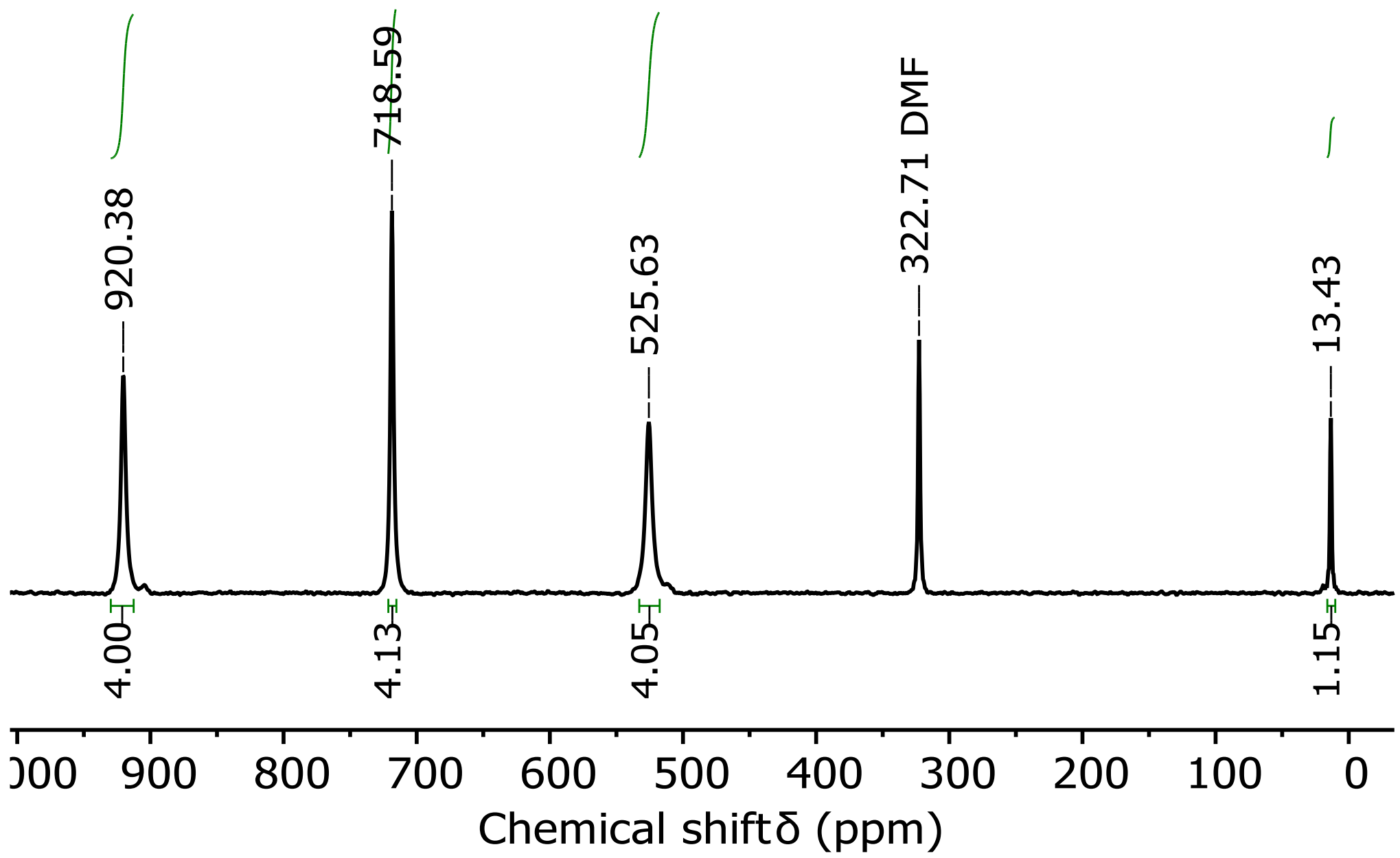

Figure S1. ^{17}O NMR spectrum (67.8 MHz) of **2-Th(Mo$_5$)$_2$** in DMF. Spectrum acquired at 21 °C.

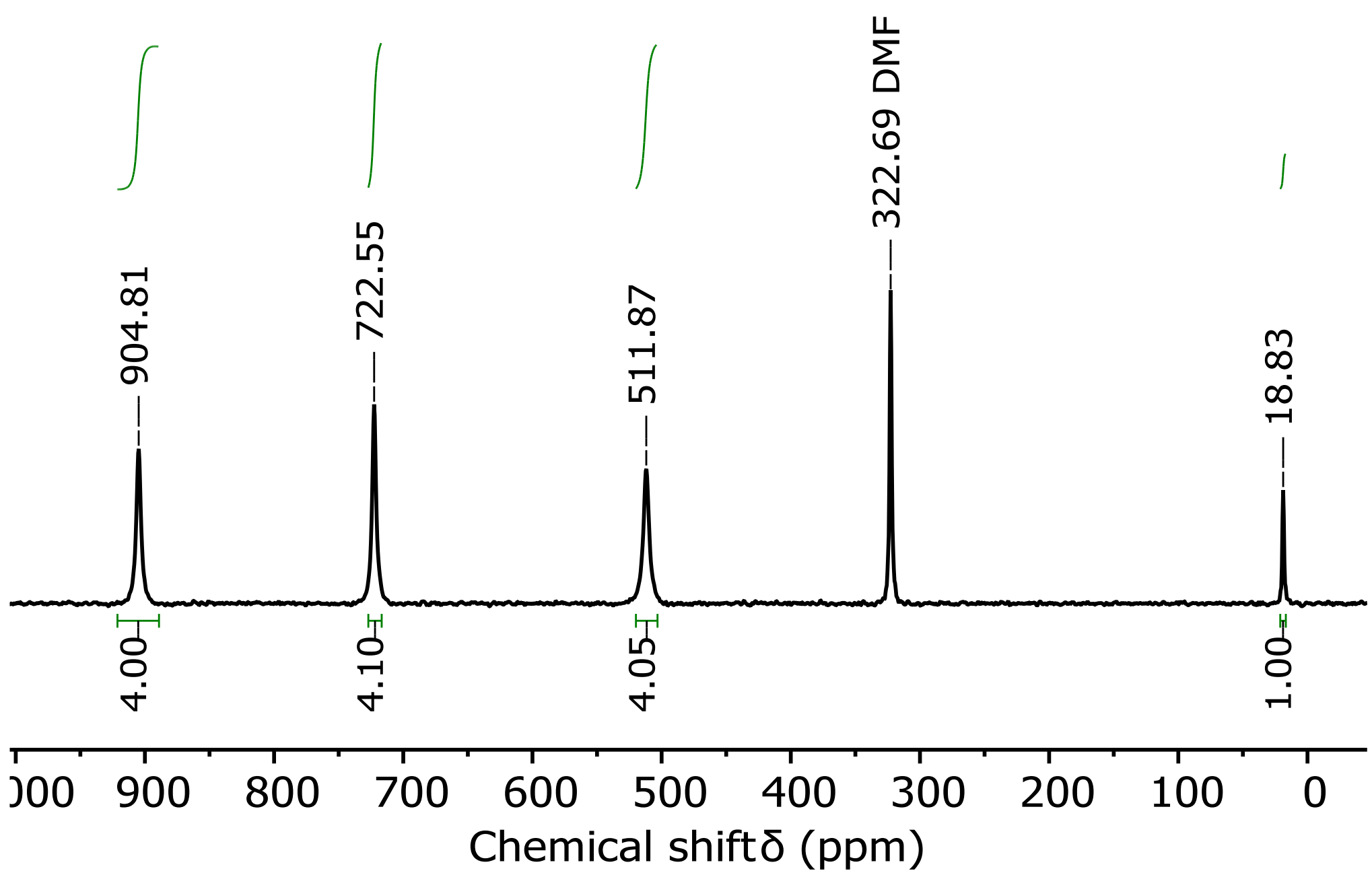

Figure S2. ^{17}O NMR spectrum (67.8 MHz) obtained during the reaction of **1-NaMo$_5$** with $ThCl_4$•2DME in DMF**.** Spectrum acquired at 21 °C.

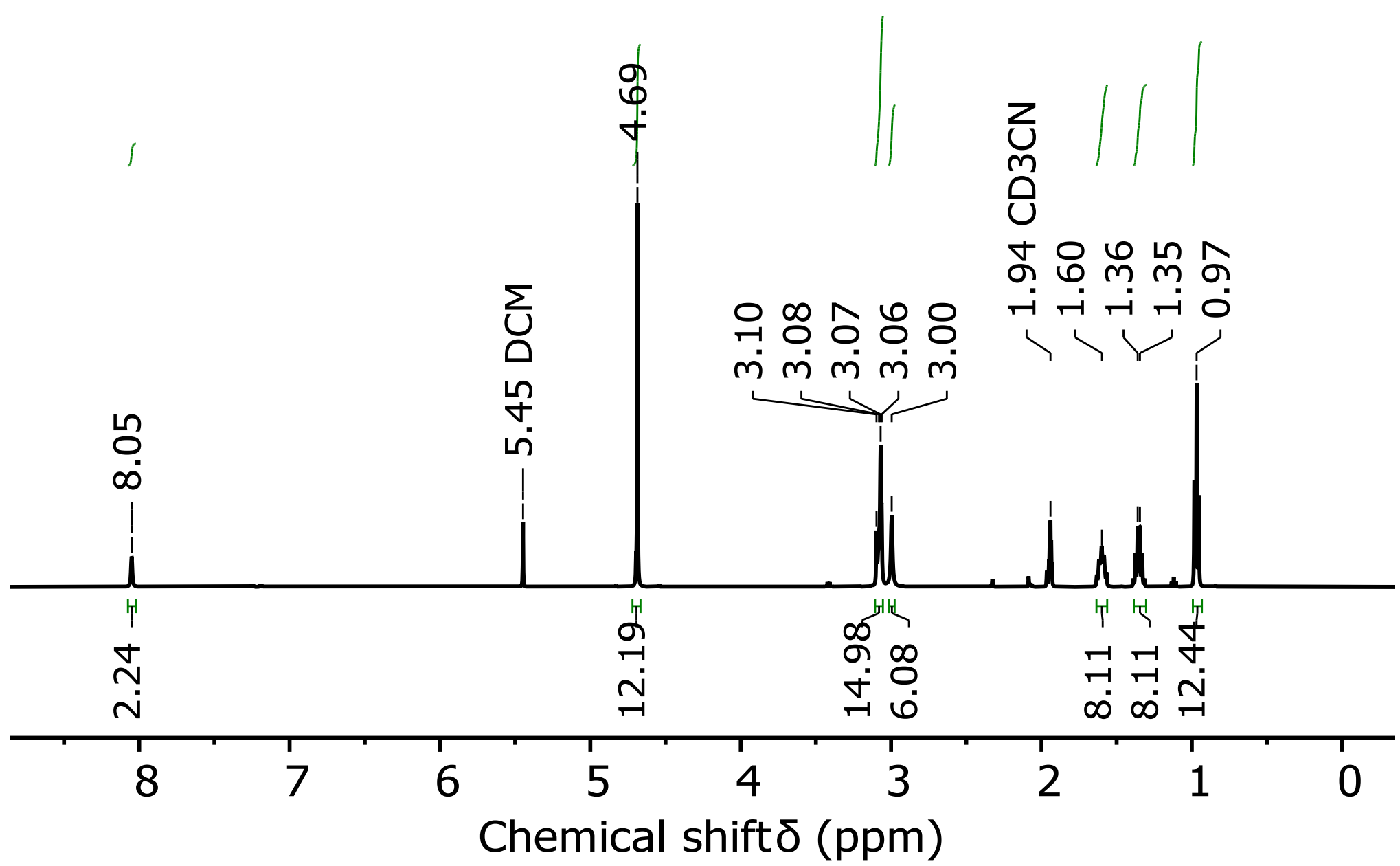

Figure S3. ^{1}H NMR spectrum (500 MHz) of **3-ThCl$_2$(DMF)$_2$Mo$_5$** in CD_3CN. Spectrum acquired at 21 °C.

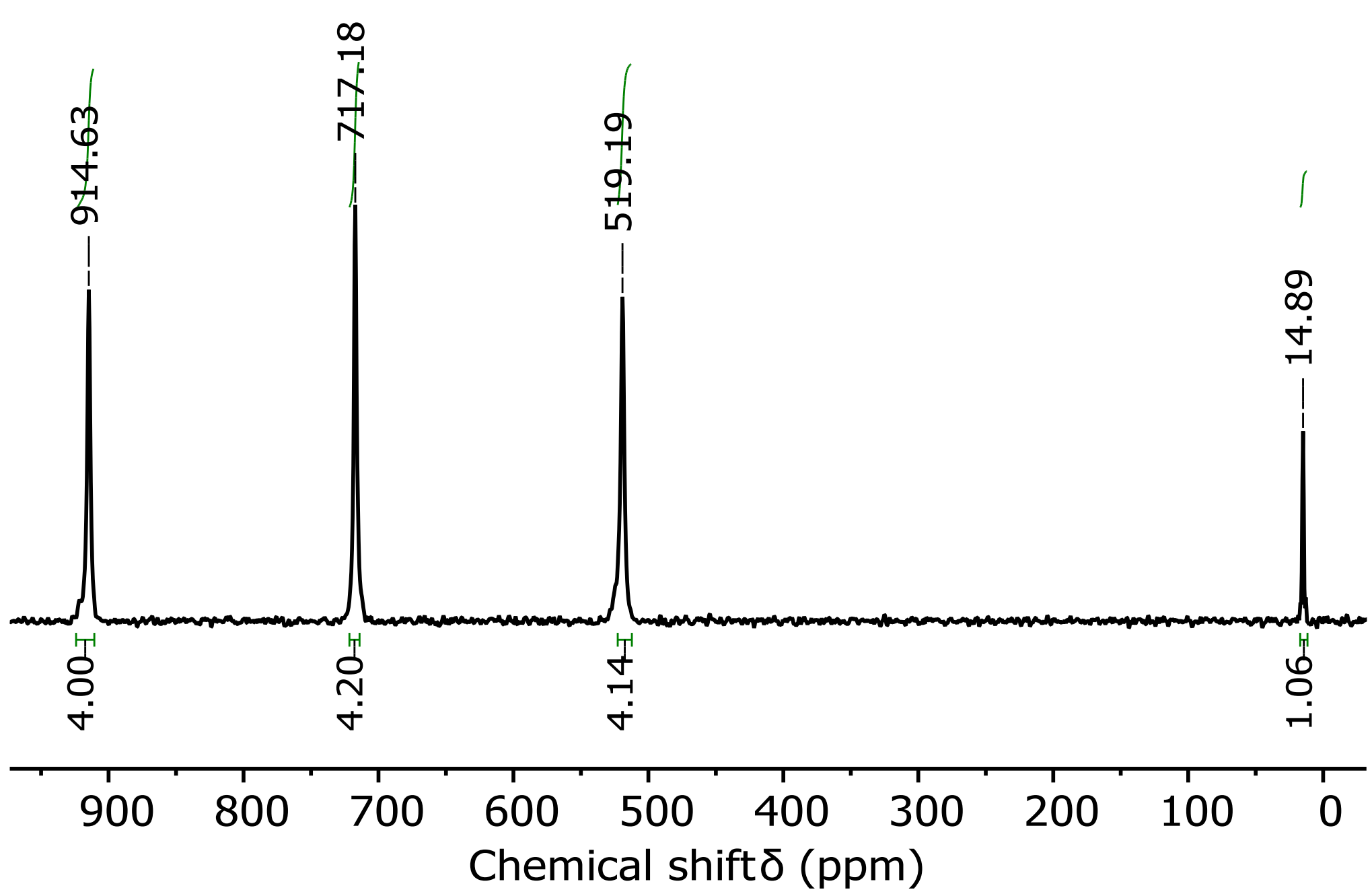

Figure S4. ^{17}O NMR spectrum (67.8 MHz) of **3-ThCl$_2$(DMF)$_2$Mo$_5$** in CD_3CN. Spectrum acquired at 21 °C.

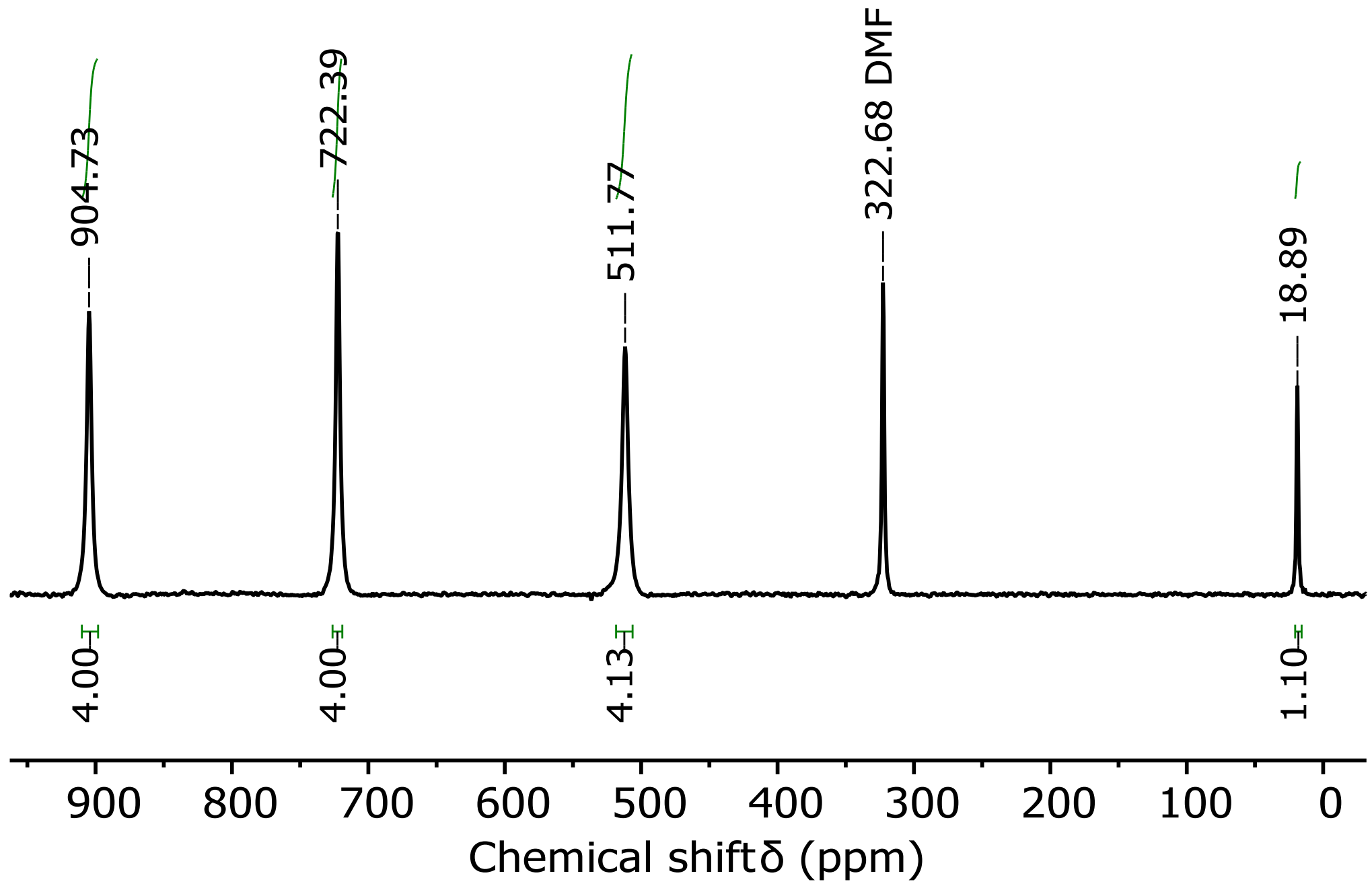

Figure S5. ^{17}O NMR spectrum (67.8 MHz) obtained during the reaction of **2-Th(Mo$_5$)$_2$** with $ThCl_4$•2DME in DMF**.** Spectrum acquired at 21 °C.

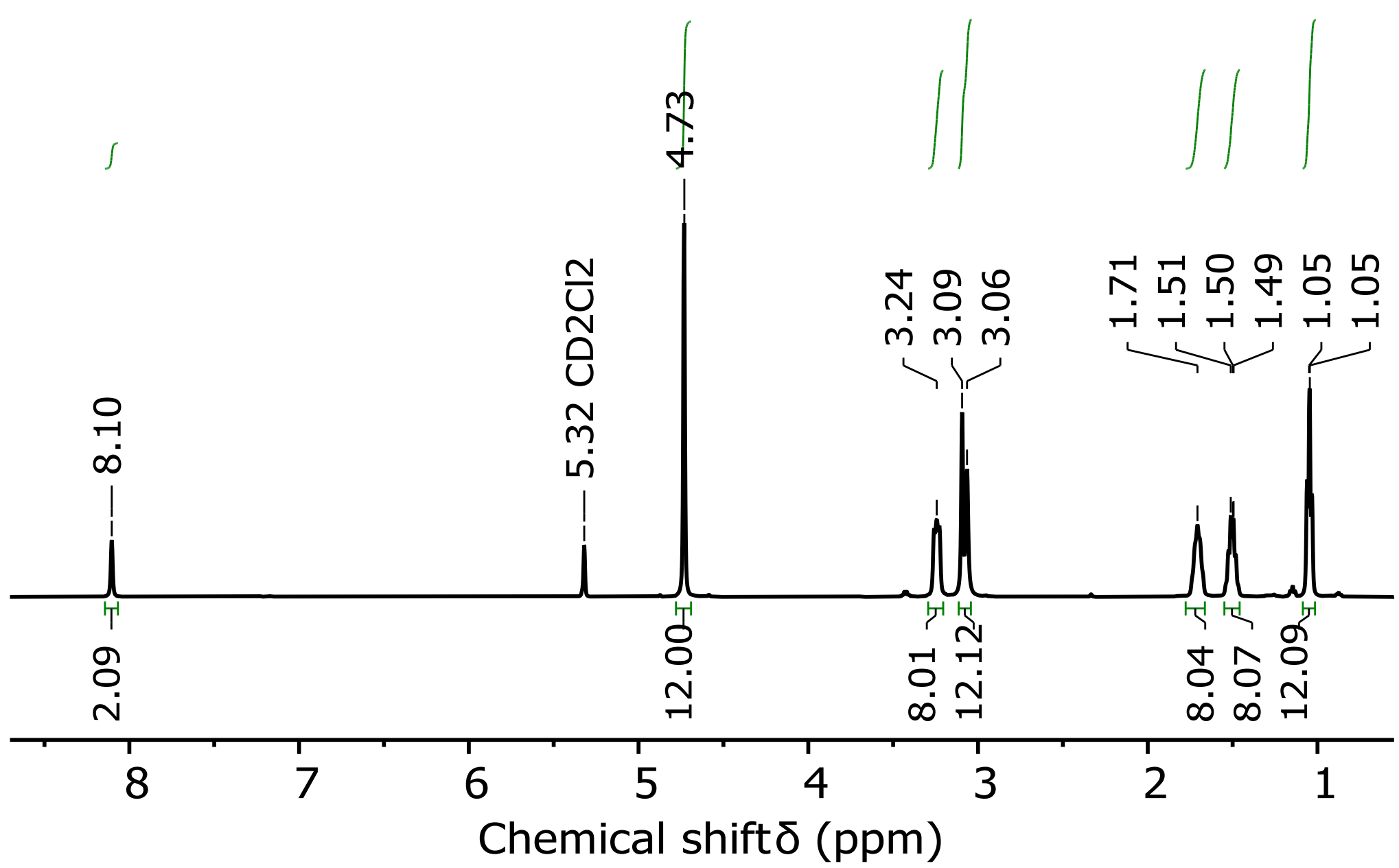

Figure S6. ^{1}H NMR spectrum (500 MHz) of **3-ThCl$_2$(DMF)$_2$Mo$_5$** obtained from the reaction of **2-Th(Mo$_5$)$_2$** with $ThCl_4$•2DME in DMF. Spectrum acquired in CD_2Cl_2 at 21 °C.

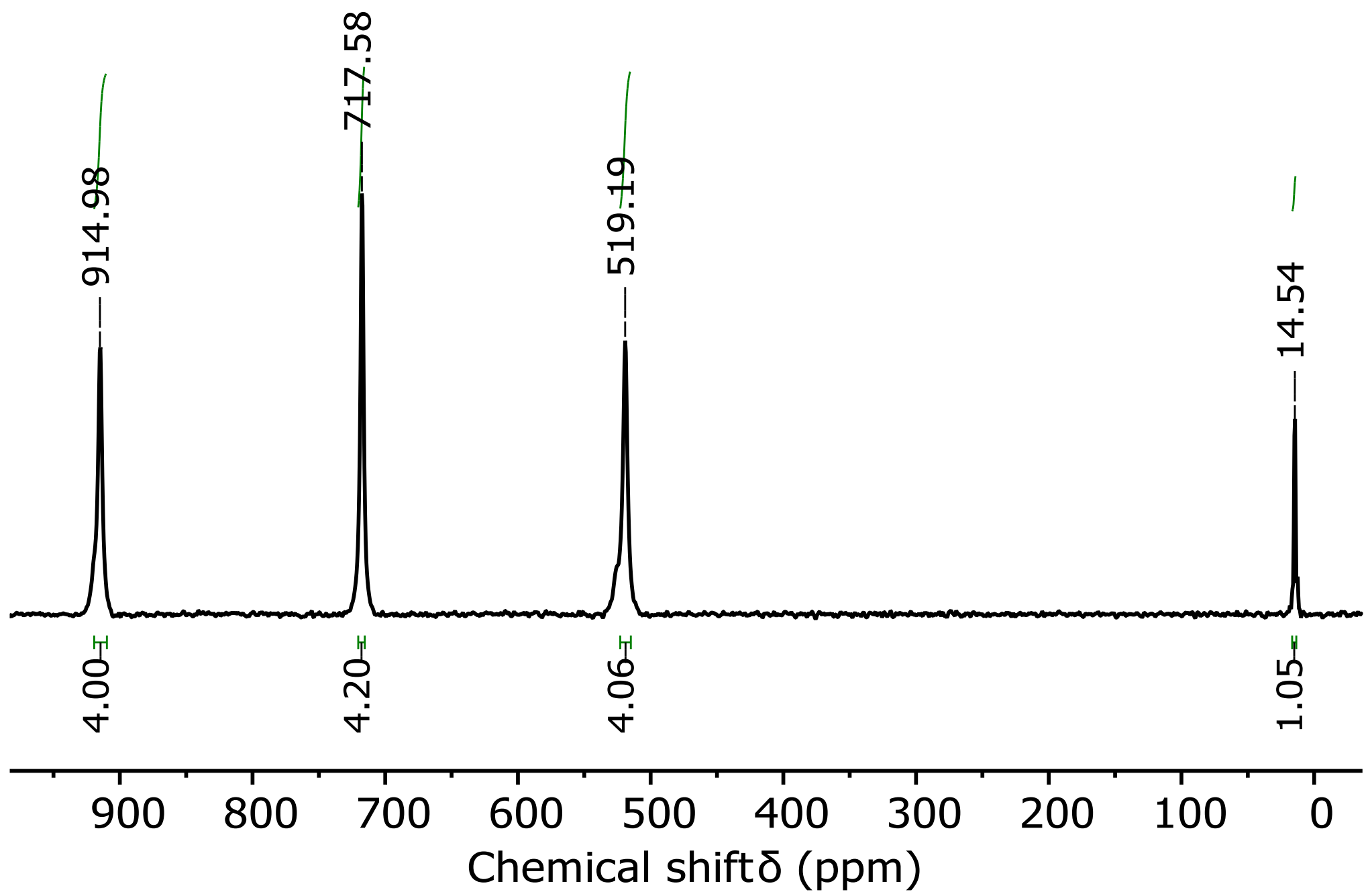

Figure S7. ^{17}O NMR spectrum (67.8 MHz) of **3-ThCl$_2$(DMF)$_2$Mo$_5$** obtained from the reaction of **2-Th(Mo$_5$)$_2$** with $ThCl_4$•2DME in DMF. Spectrum acquired in CD_2Cl_2 at 21 °C

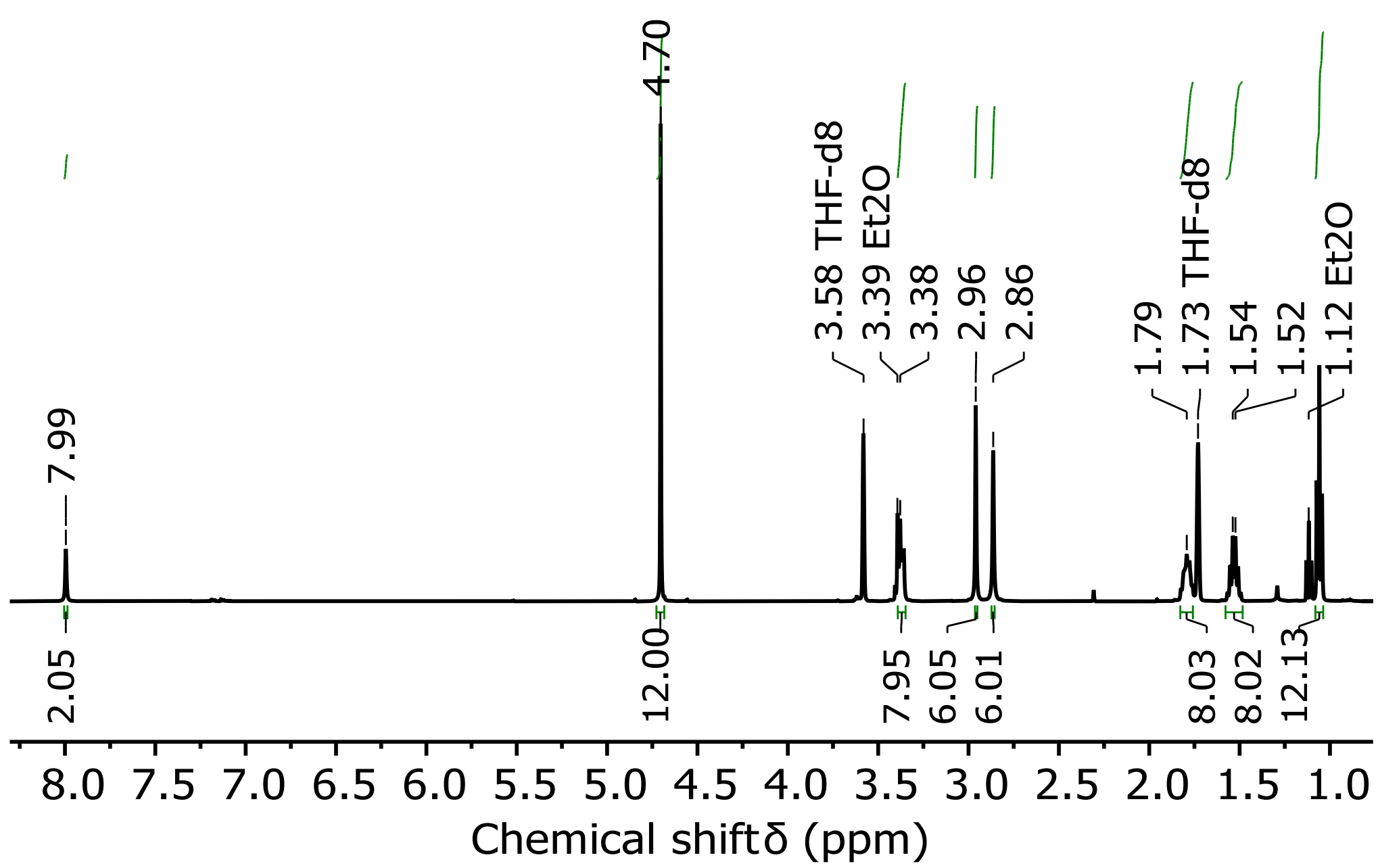

Figure S8. ^{1}H NMR spectrum (500 MHz) of **3-ThCl$_2$(DMF)$_2$Mo$_5$** inTHF-d_8. Spectrum acquired at 21 °C.

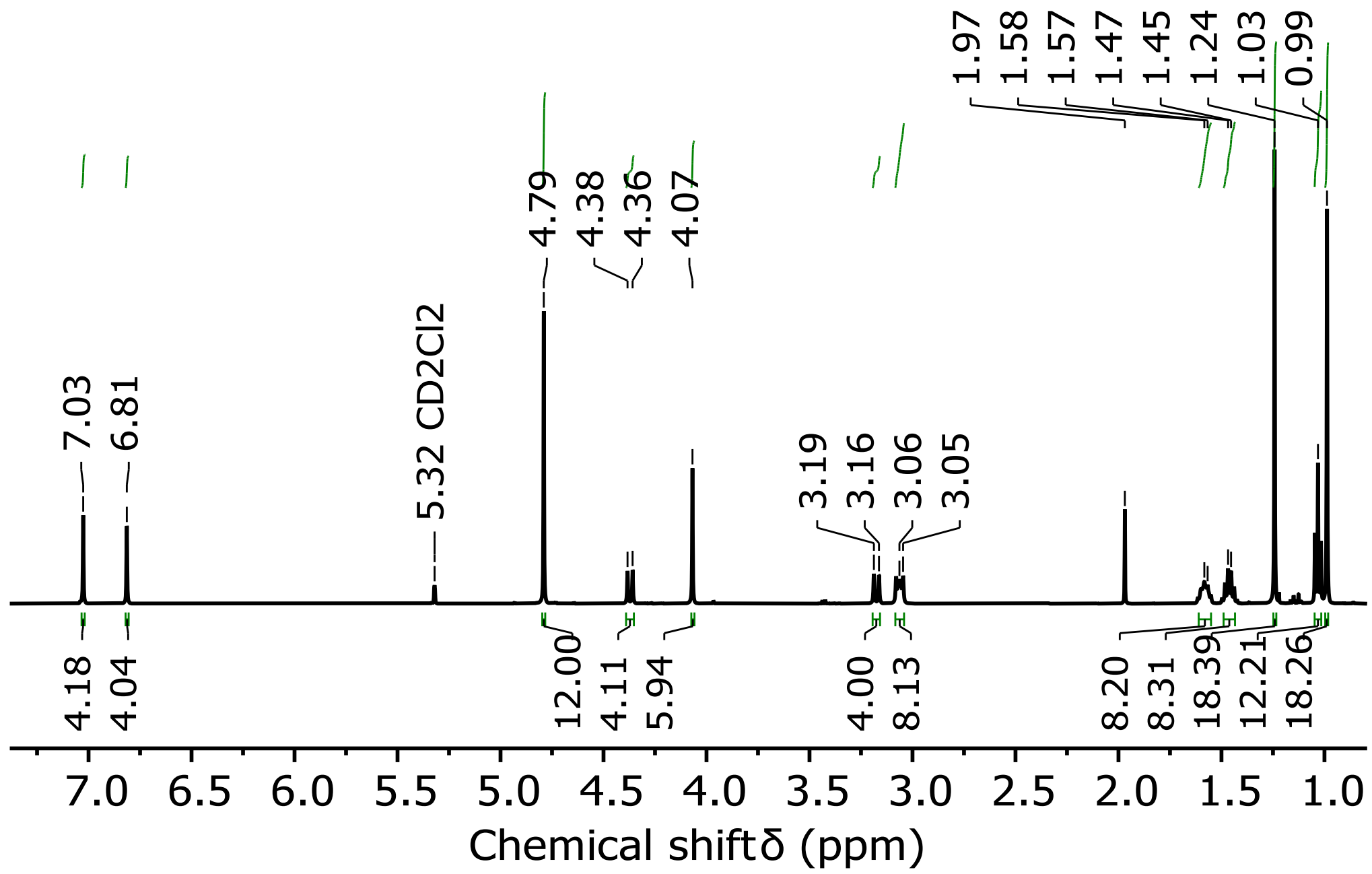

Figure S9. ^{1}H NMR spectrum (500 MHz) of **5-CalixThMo$_5$** in CD_2Cl_2. Spectrum acquired at 21 °C.

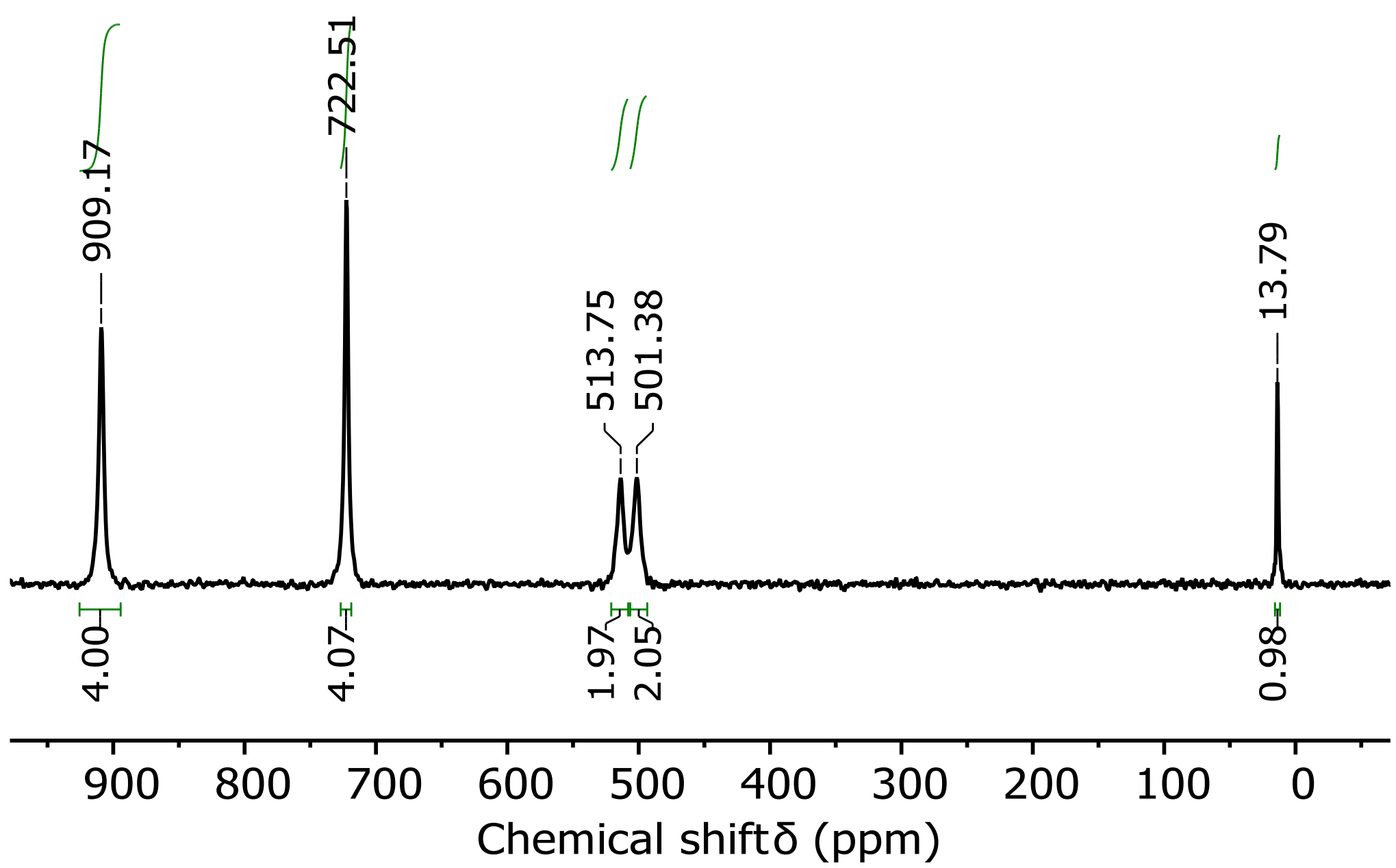

Figure S10. ^{17}O NMR spectrum (67.8 MHz) of **5-CalixThMo$_5$** in CD_2Cl_2. Spectrum acquired at 21 °C.

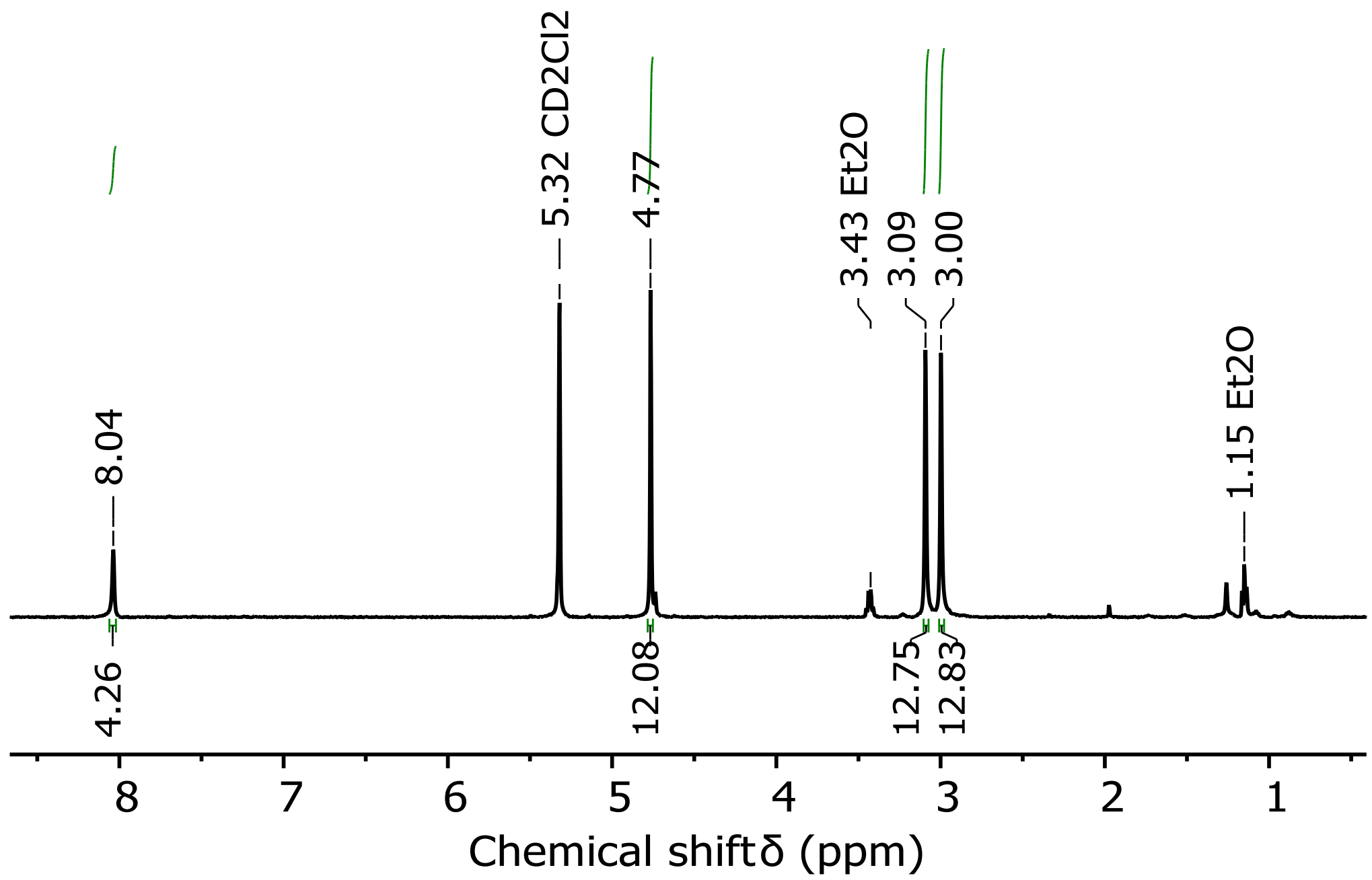

Figure S11. ^{1}H NMR spectrum (500 MHz) of **6-ThCl(DMF)$_4$Mo$_5$** in CD_2Cl_2. Spectrum acquired at 21 °C.

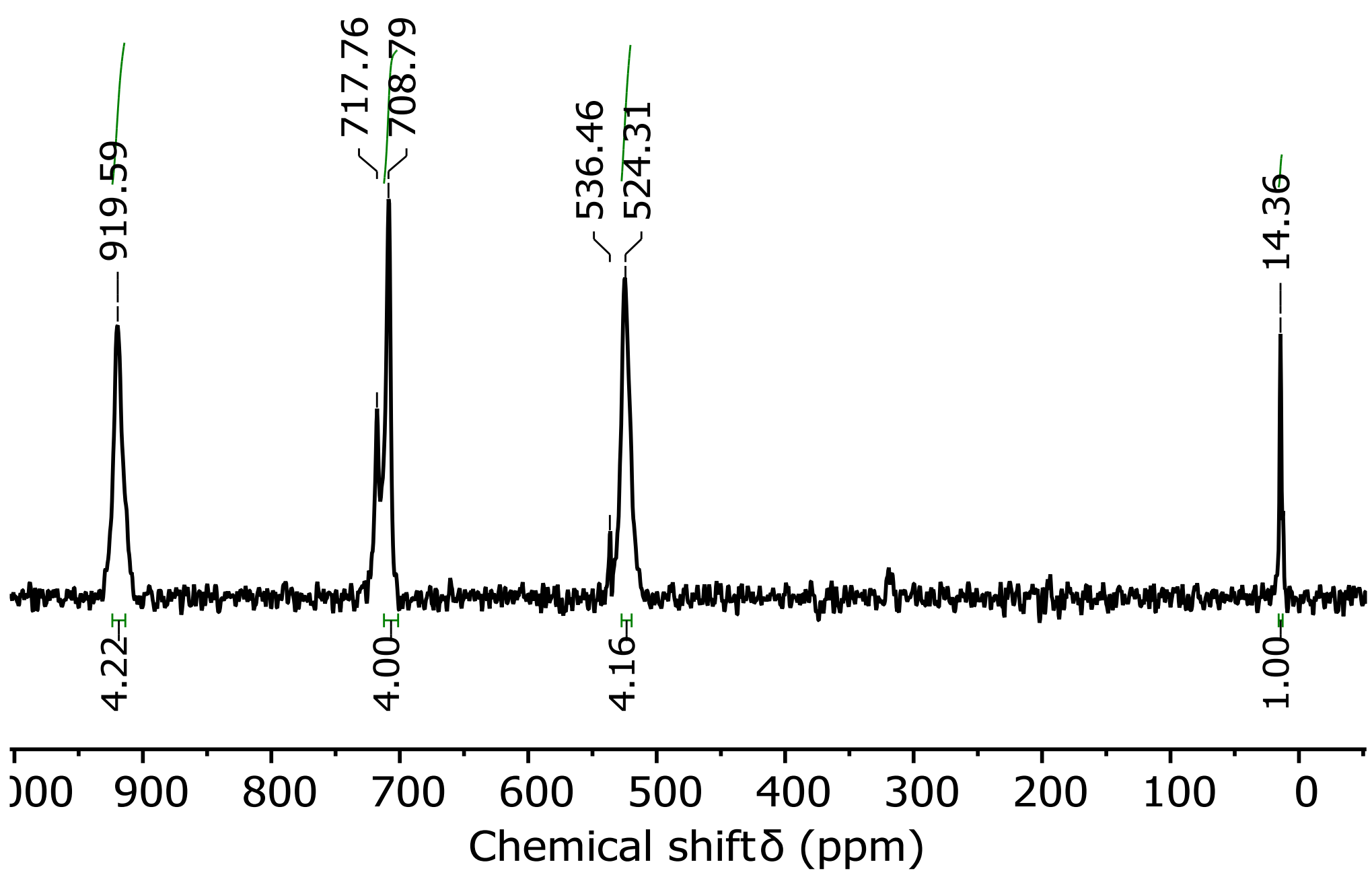

Figure S12. ^{17}O NMR spectrum (67.8 MHz) of **6-ThCl(DMF)$_4$Mo$_5$** in CD_2Cl_2. Spectrum acquired at 21 °C. Minor peaks are 536.46 and 717.76 ppm are attributed to **3-ThCl$_2$(DMF)$_2$Mo$_5$**.

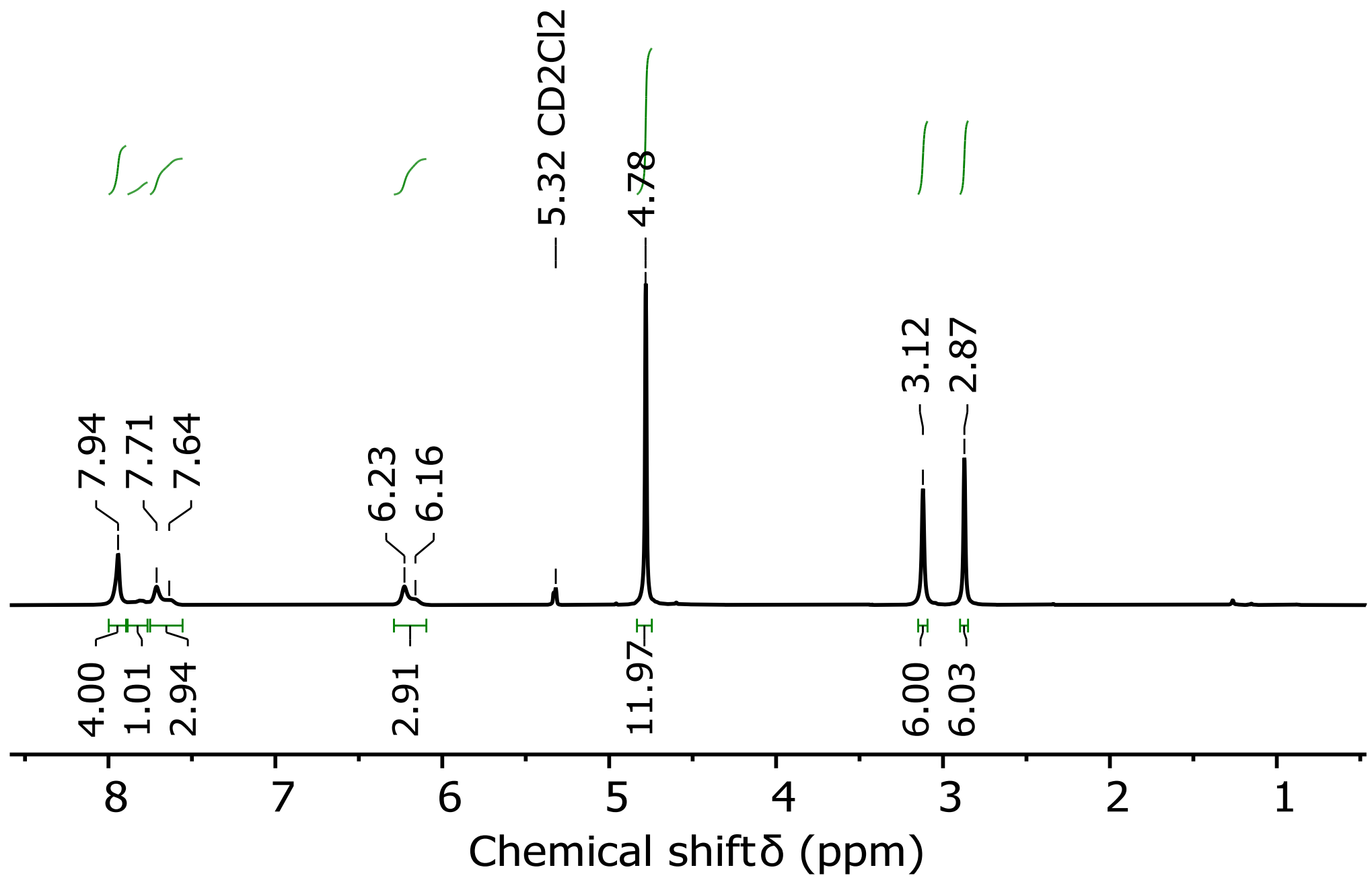

Figure S13. ^{1}H NMR spectrum (500 MHz) of **7-TpTh(DMF)$_2$Mo$_5$** in CD_2Cl_2. Spectrum acquired at 21 °C.

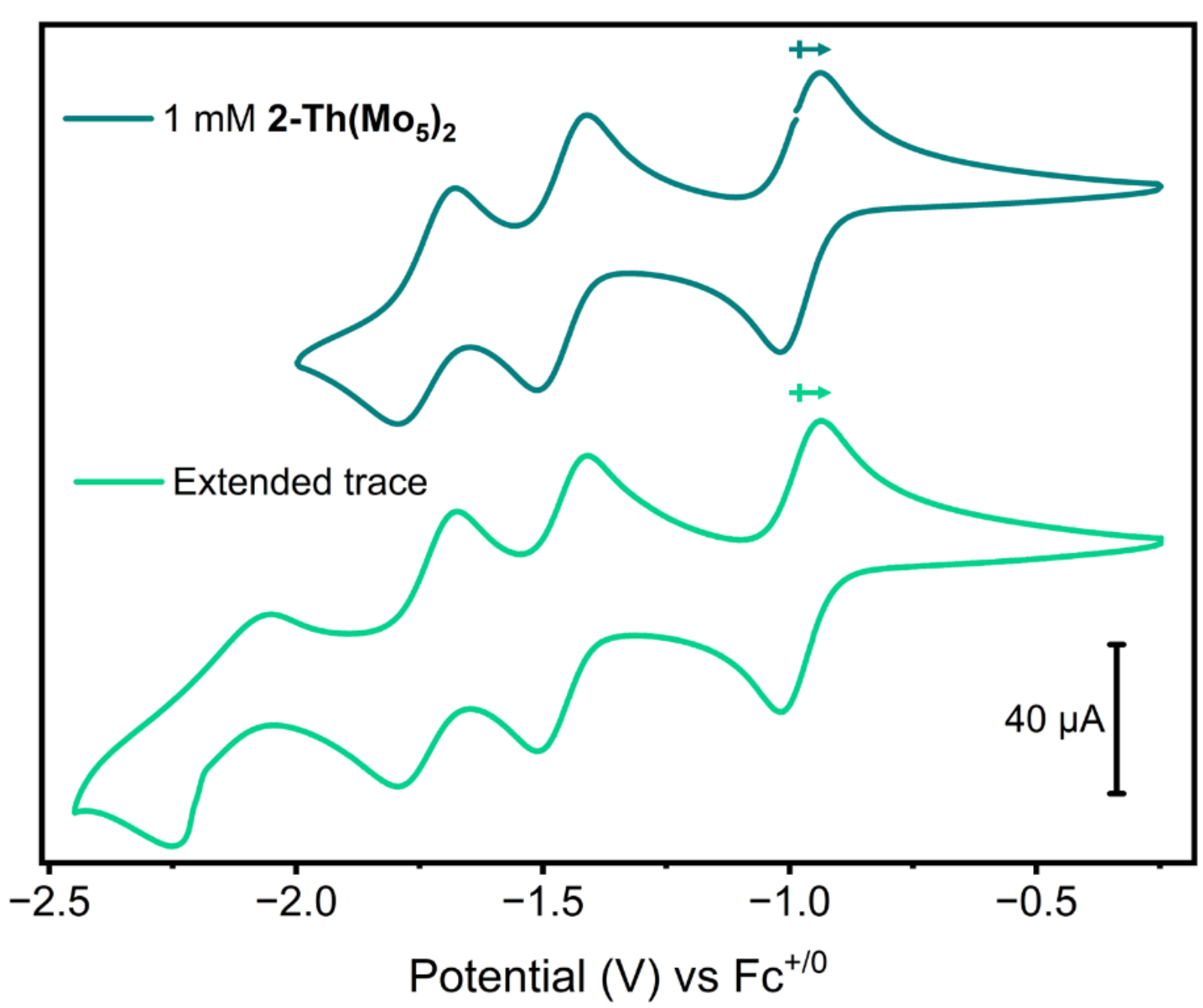

Figure S14. CV of **2-Th(Mo$_5$)$_2$** (1 mM) recorded in DCM with 100 mM of TBA(PF_6) supporting electrolyte. The CV was acquired at a scan rate of 200 mv/s at 21 °C.

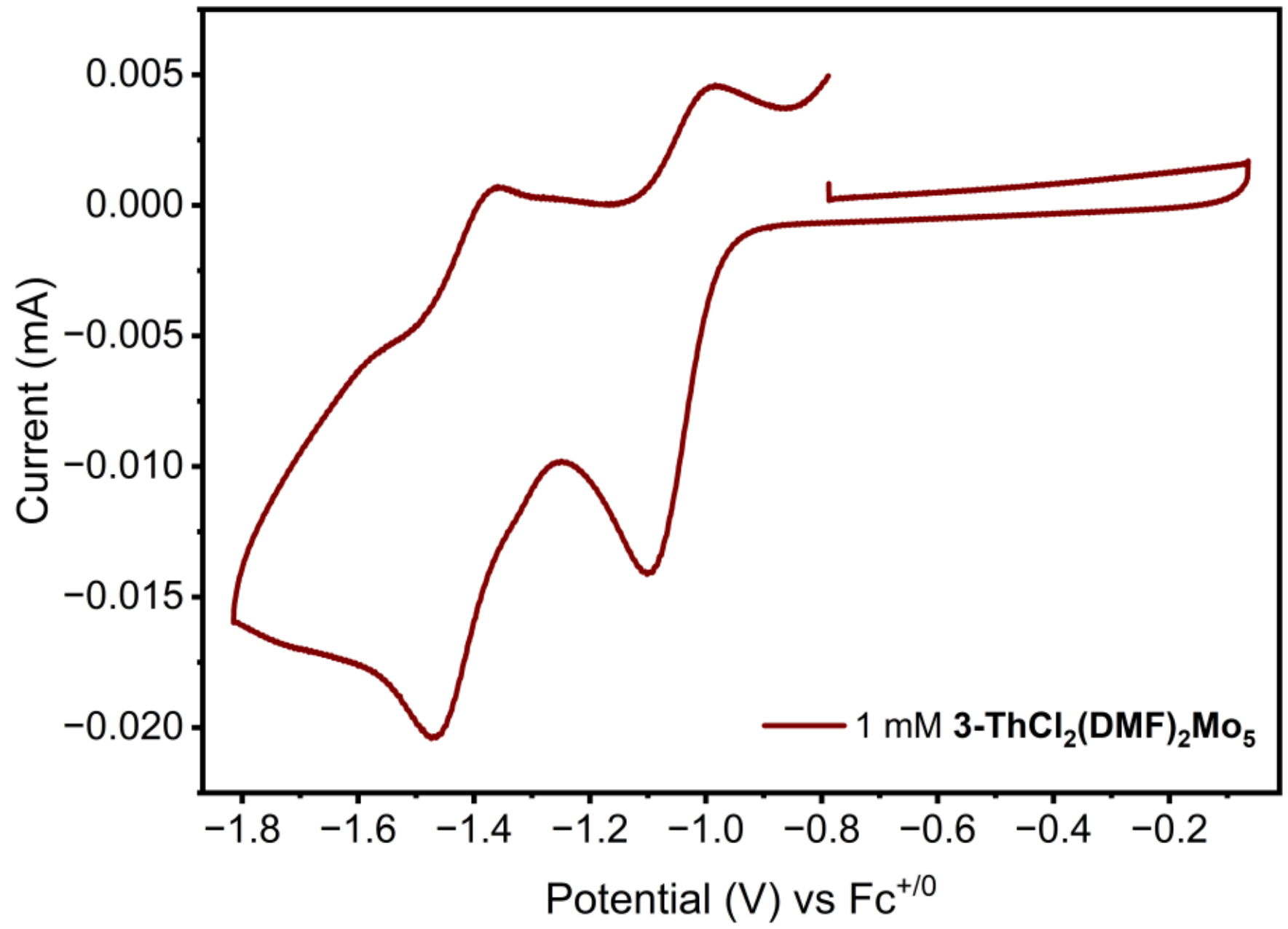

Figure S15. 1st scan of the CV of **3-ThCl$_2$(DMF)$_2$Mo$_5$** (1 mM) recorded in DMF with 100 mM of TBA(PF$_6$) supporting electrolyte. The CV was acquired at a scan rate of 200 mv/s at 21 °C.

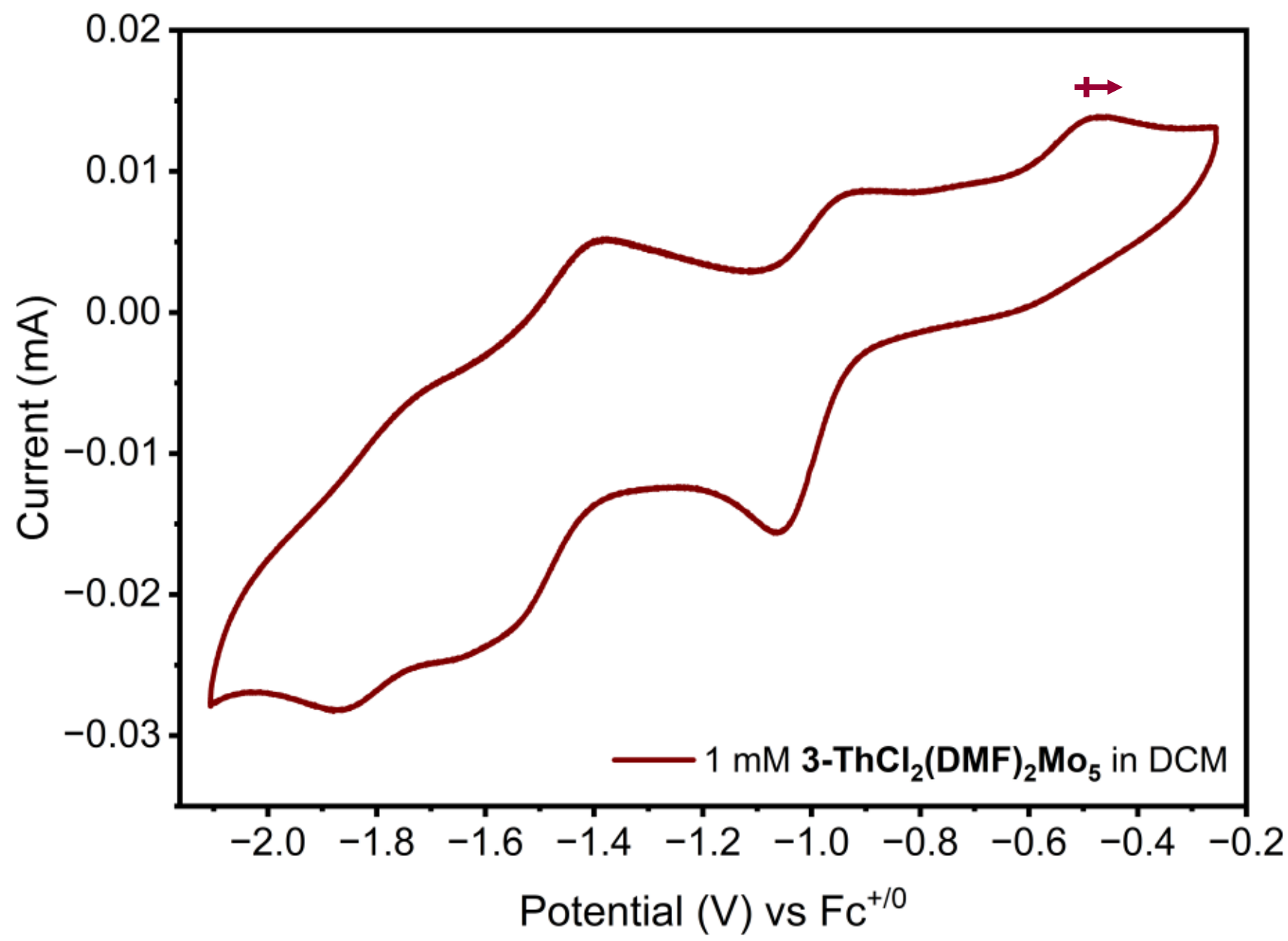

Figure S16. CV of **3-ThCl$_2$(DMF)$_2$Mo$_5$** (1 mM) recorded in DCM with 100 mM of TBA(PF$_6$) supporting electrolyte. The CV was acquired at a scan rate of 200 mv/s at 21 °C.

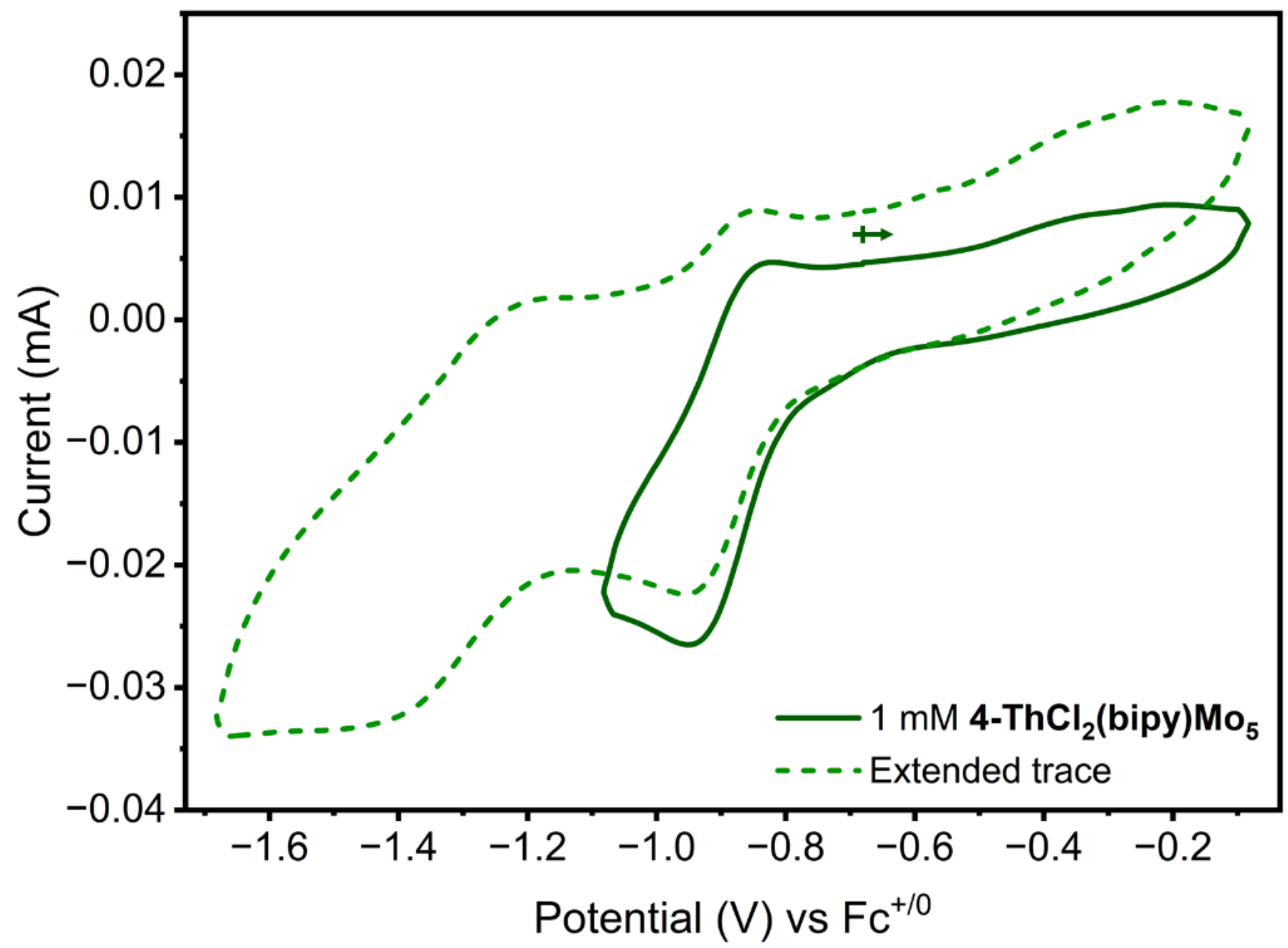

Figure S17. CV of **4-ThCl$_2$(bipy)Mo$_5$** (1 mM) recorded in DCM with 100 mM of TBA(PF$_6$) supporting electrolyte. The CV was acquired at a scan rate of 200 mv/s at 21 °C.

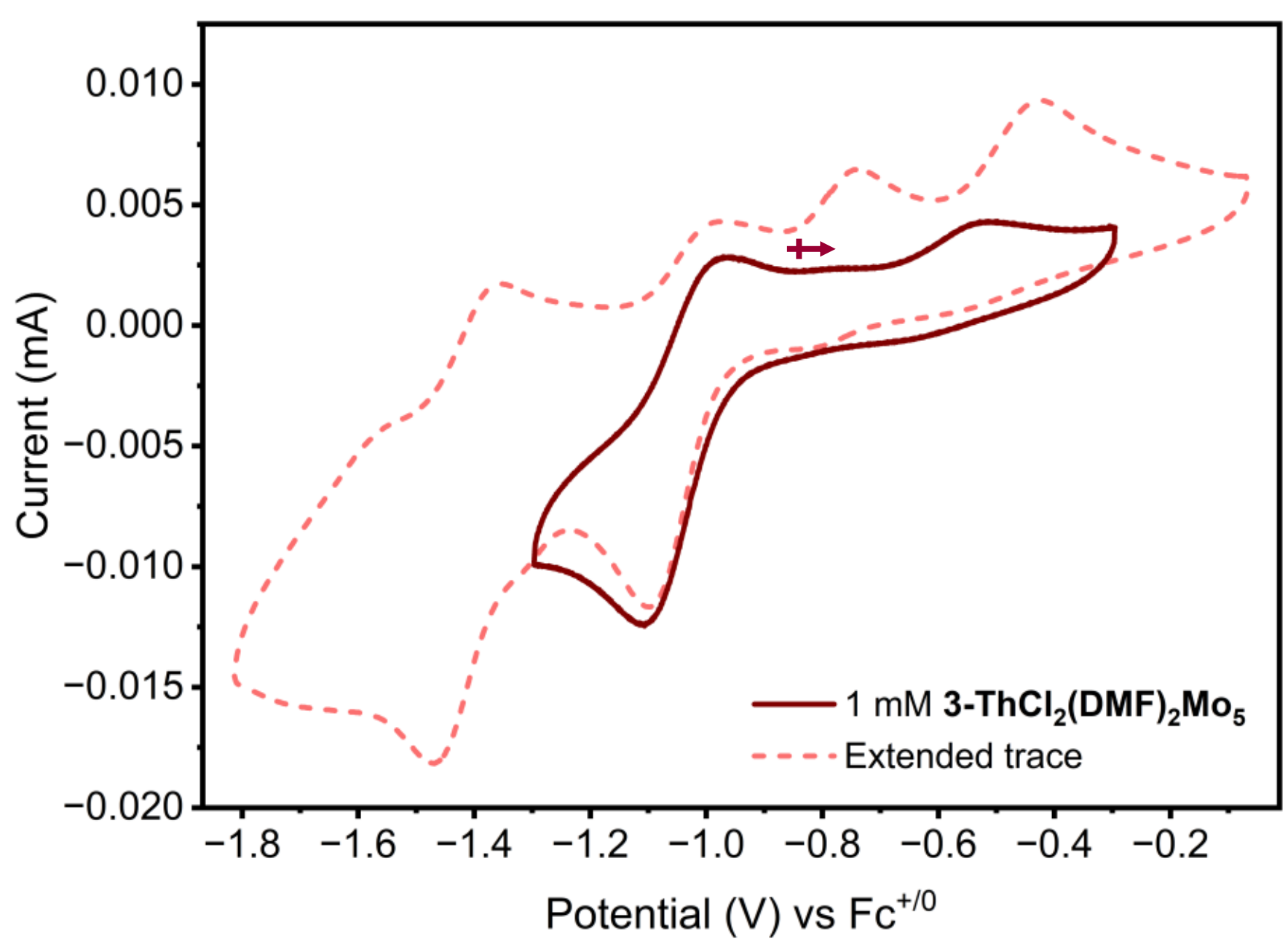

Figure S18. CV of **3-ThCl$_2$(DMF)$_2$Mo$_5$** (1 mM) with the first wave isolated. The CV was recorded in DMF with 100 mM of TBA(PF$_6$) supporting electrolyte, a scan rate of 200 mv/s and at 21 °C.

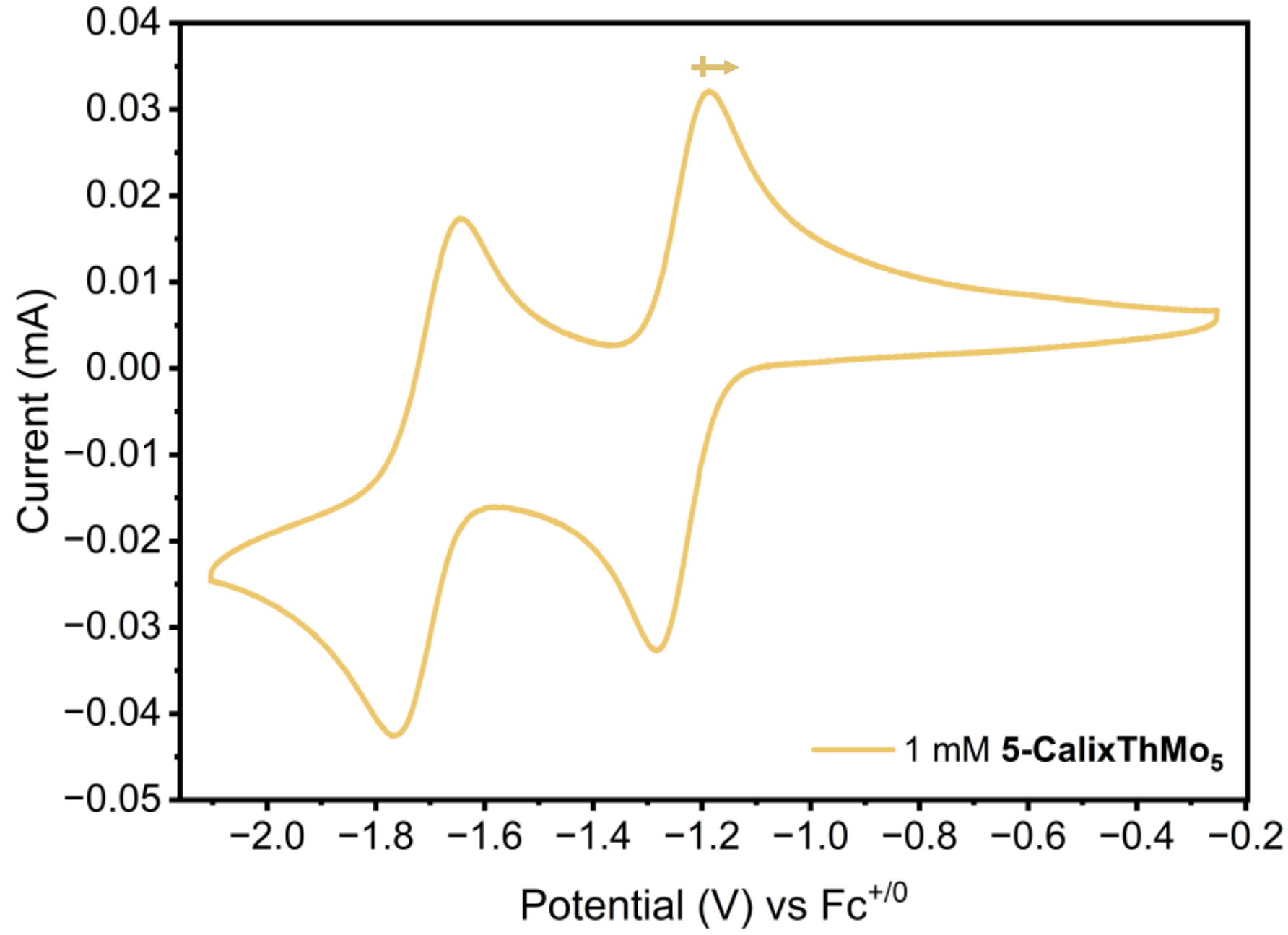

Figure S19. CV highlighting the reductive events of **5-CalixThMo$_5$** (1 mM) recorded in DCM with 100 mM of TBA(PF_6) supporting electrolyte. The CV was acquired at a scan rate of 200 mv/s at 21 °C.

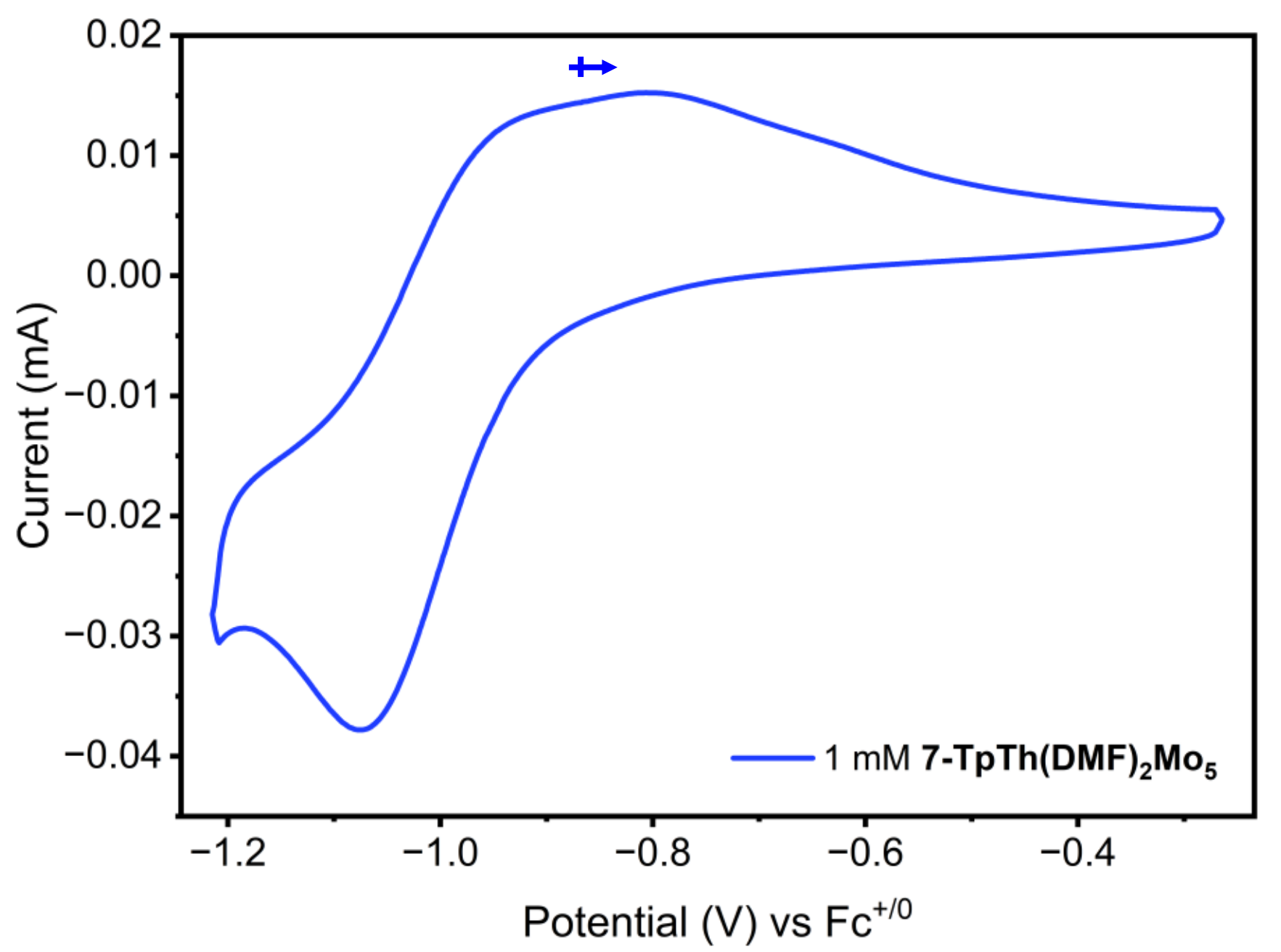

Figure S20. CV of **7-TpTh(DMF)$_2$Mo$_5$** (1 mM) with the first wave isolated. The CV was recorded in DCM with 100 mM of TBA(PF_6) supporting electrolyte, a scan rate of 200 mv/s and at 21 °C.

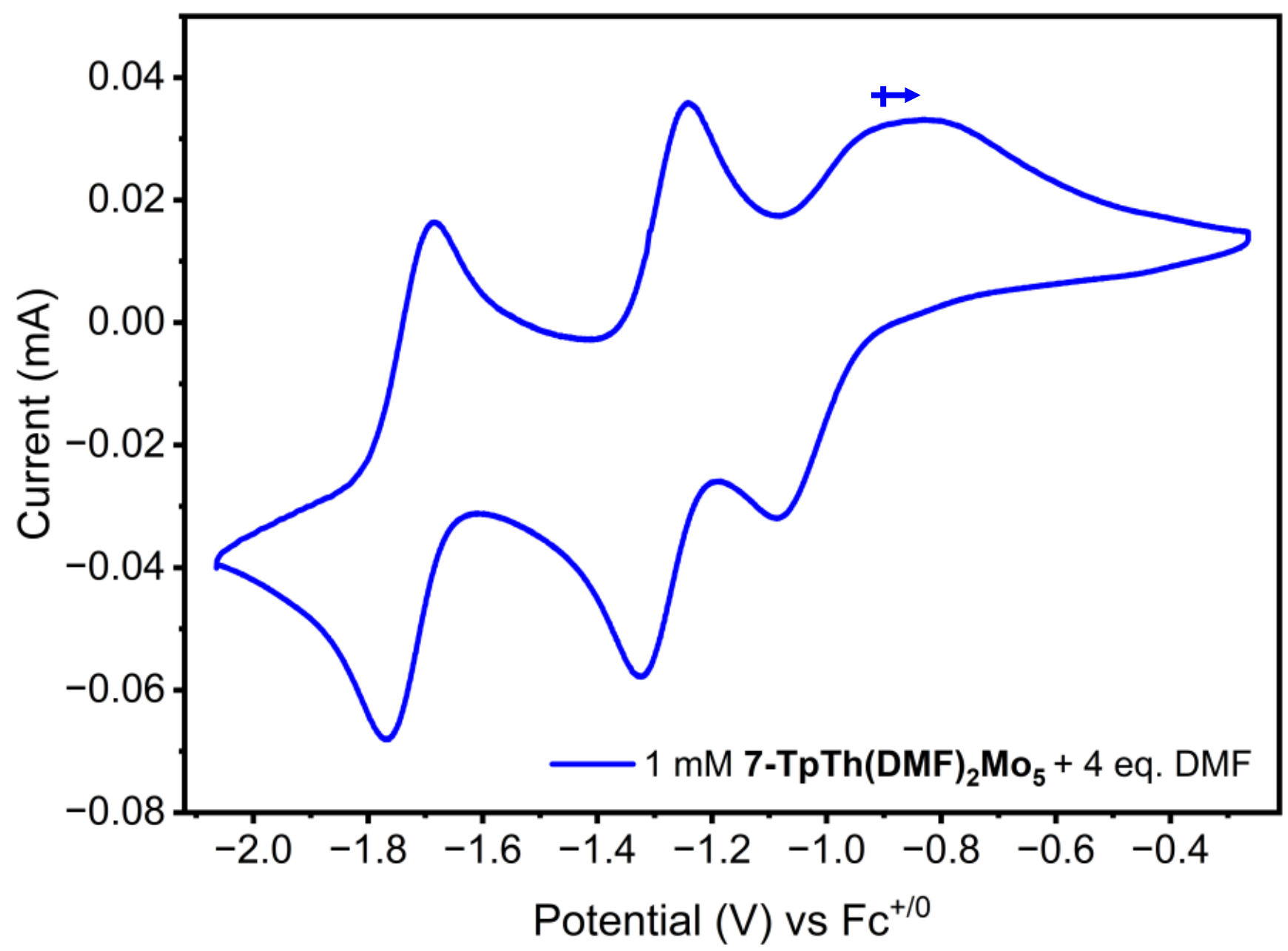

Figure S21. CV of **7-TpTh(DMF)$_2$Mo$_5$** (1 mM) with 4 eq. of DMF. The CV was recorded in DCM with 100 mM of TBA(PF$_6$) supporting electrolyte, a scan rate of 200 mv/s and at 21 °C.

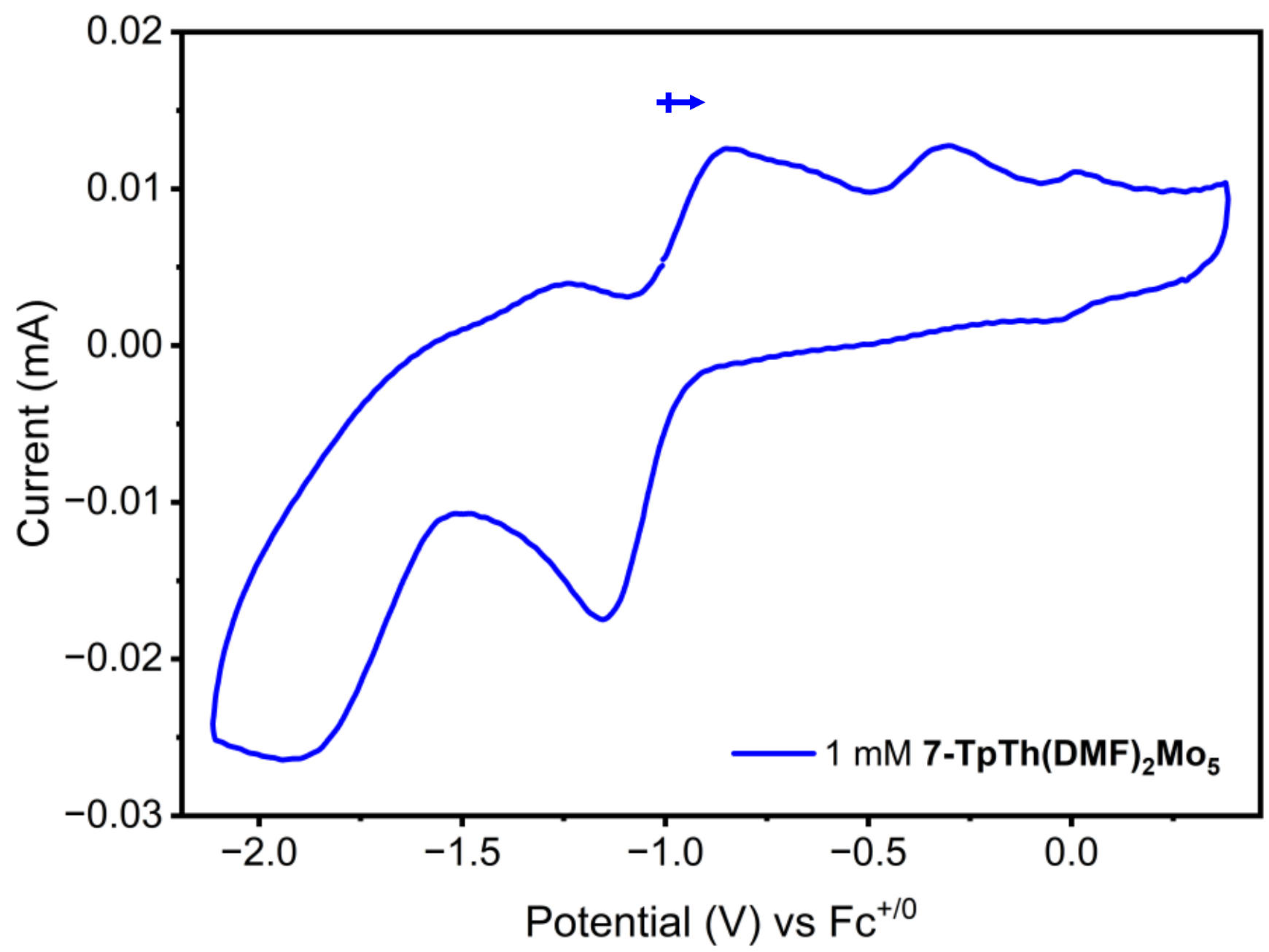

Figure S22. CV of **7-TpTh(DMF)$_2$Mo$_5$** (1 mM) recorded in DMF with 100 mM of TBA(PF$_6$) supporting electrolyte. The CV was acquired at a scan rate of 200 mv/s at 21 °C.

Table S1: Crystal data and structure refinement for **3-ThCl$_2$(DMF)$_2$Mo$_5$**

Empirical formula	C27 H64 Cl4 Mo5 N4 O20 Th	
Formula weight	1618.36	
Temperature	100.00(10) K	
Wavelength	1.54184 Å	
Crystal system	monoclinic	
Space group	$P2_1/n$	
Unit cell dimensions	*a* = 13.71737(5) Å	α = 90°
	b = 22.34591(8) Å	β = 97.4976(3)°
	c = 16.90355(6) Å	γ = 90°
Volume	5137.09(3) Å^3	
Z	4	
Density (calculated)	2.093 Mg/m^3	
Absorption coefficient	21.429 mm^{-1}	
F(000)	3128	
Crystal color, morphology	light violet, block	
Crystal size	0.181 x 0.164 x 0.056 mm^3	
Theta range for data collection	3.296 to 80.333°	
Index ranges	$-17 \le h \le 17$, $-28 \le k \le 25$, $-21 \le l \le 21$	
Reflections collected	88087	
Independent reflections	11080 [*R*(int) = 0.0473]	
Observed reflections	10980	
Completeness to theta = 74.504°	100.0%	
Absorption correction	Multi-scan	
Max. and min. transmission	1.00000 and 0.30966	
Refinement method	Full-matrix least-squares on F^2	
Data / restraints / parameters	11080 / 0 / 563	
Goodness-of-fit on F^2	1.128	
Final *R* indices [I>2sigma(I)]	*R*1 = 0.0289, *wR*2 = 0.0699	
R indices (all data)	*R*1 = 0.0293, *wR*2 = 0.0701	
Extinction coefficient	0.000098(6)	
Largest diff. peak and hole	1.326 and -1.012 e.Å^{-3}	

Table S2: Continuous Shape Measures (CShM) analysis for **3-ThCl$_2$(DMF)$_2$Mo$_5$**. Positions in Cartesian coordinates (x,y,z).

Structure	**3-ThCl$_2$(DMF)$_2$Mo$_5$**		
Th	5.7619	4.7530	7.8615
Cl	6.5213	3.6942	5.4435
Cl	3.5225	3.3197	8.4881
O	6.6650	6.8052	6.9022
O	4.6533	6.6517	8.8984
O	6.1583	4.7807	10.2621
O	8.1638	4.9331	8.2813
O	4.1796	5.6184	6.2751
O	6.5903	2.5423	8.4700

OP-8	Ideal structure	CShM =	31.04476	
Th	M	5.8018	4.7887	7.8758
Cl	L1	7.2796	4.9258	6.4110
Cl	L5	4.3240	4.6516	9.3406
O	L2	6.0415	5.8906	6.1218
O	L4	3.9516	5.6966	8.1933
O	L6	5.5620	3.6868	9.6298
O	L8	7.6519	3.8808	7.5583
O	L3	4.6630	6.2098	6.8600
O	L7	6.9405	3.3676	8.8916

HPY-8	Ideal structure	CShM =	23.39961	
Th	M	6.0073	4.9063	7.8095
Cl	L2	6.1196	3.6755	5.9736
Cl	L1	4.1573	3.8480	8.4059
O	L4	4.8031	6.6469	7.1627
O	L5	5.3814	6.6680	8.9940
O	L6	6.4310	5.3625	9.9334
O	L7	7.1616	3.7135	9.2735
O	L3	5.1316	5.3151	5.8185
O	L8	7.0230	2.9627	7.5112

HBPY-8	Ideal structure	CShM =	16.37553	
Th	M	5.8018	4.7887	7.8758
Cl	L2	5.6178	3.8723	5.7783
Cl	L1	4.3351	3.2203	8.6896
O	L8	7.2685	6.3571	7.0620
O	L4	4.3718	6.4636	8.5264
O	L5	5.9857	5.7051	9.9733
O	L6	7.4156	4.0303	9.3227
O	L3	4.1879	5.5471	6.4289
O	L7	7.2317	3.1138	7.2252

CU-8	Ideal structure	CShM =	11.16225	
Th	M	5.8018	4.7887	7.8758
Cl	L1	6.0854	4.3885	5.5603
Cl	L3	4.2991	3.1956	8.7735
O	L5	7.3045	6.3818	6.9781
O	L6	4.9301	6.9653	8.1993
O	L7	5.5182	5.1889	10.1913
O	L8	7.8925	4.6054	8.9700
O	L2	3.7111	4.9720	6.7816

O	L4	6.6734	2.6121	7.5523

SAPR-8 Ideal structure CShM = 1.65654

Th	M	5.8018	4.7887	7.8758
Cl	L1	6.7542	4.0023	5.7134
Cl	L3	3.7177	3.7164	8.7175
O	L5	6.6132	6.8483	6.7351
O	L8	4.4660	6.6461	8.8593
O	L7	6.1220	4.5878	10.3372
O	L6	8.2691	4.7899	8.2130
O	L4	4.0651	5.3148	6.1704
O	L2	6.4069	2.4039	8.2605

TDD-8 Ideal structure CShM = 1.97559

Th	M	5.8018	4.7887	7.8758
Cl	L2	6.2948	3.8219	5.6390
Cl	L5	3.8428	3.3522	8.4051
O	L3	6.7295	6.6425	6.5032
O	L4	4.9102	6.8941	8.8523
O	L8	5.8129	4.4741	10.3420
O	L7	8.1593	5.0866	8.6069
O	L1	3.9650	5.5318	6.3739
O	L6	6.6997	2.5065	8.2841

JGBF-8 Ideal structure CShM = 16.27373

Th	M	5.8018	4.7887	7.8758
Cl	L3	5.5625	3.9463	6.2166
Cl	L4	4.6416	3.5484	8.6728
O	L6	6.9620	6.0290	7.0788
O	L8	3.5595	5.9709	8.6596
O	L5	6.0410	5.6311	9.5350
O	L2	8.2833	4.4489	8.7512
O	L7	4.4805	6.3689	6.2034
O	L1	6.8838	2.3662	7.8890

JETBPY-8 Ideal structure CShM = 29.57985

Th	M	5.8018	4.7887	7.8758
Cl	L1	6.6074	4.3248	6.4230
Cl	L8	4.1386	3.2103	9.7681
O	L7	7.4649	6.3671	5.9835
O	L4	4.1595	4.9807	8.3664
O	L6	6.0068	4.4612	9.5567
O	L5	7.2701	5.6601	8.1193
O	L3	5.4228	6.1796	6.9290
O	L2	5.3441	3.1259	7.8604

JBTPR-8 Ideal structure CShM = 3.49107

Th	M	5.9500	5.0318	8.0486
Cl	L1	6.2456	3.8611	6.0921
Cl	L2	4.1073	3.6656	8.2017
O	L3	6.5854	6.7880	6.7078
O	L4	4.4471	6.5925	8.8174
O	L6	6.0882	4.5440	10.2911
O	L5	8.2266	4.7395	8.1814
O	L7	3.8676	5.7044	6.0002
O	L8	6.6982	2.1714	8.5419

BTPR-8	Ideal structure	CShM =	2.43903		
Th	M	5.9253	4.9913	8.0197	
Cl	L1	6.2459	3.7191	5.8943	
Cl	L2	3.9240	3.5063	8.1874	
O	L3	6.6140	6.8992	6.5621	
O	L4	4.2921	6.6864	8.8553	
O	L6	6.0769	4.4622	10.4560	
O	L5	8.3988	4.6750	8.1629	
O	L7	4.1890	5.5506	6.3135	
O	L8	6.5500	2.6082	8.4310	

JSD-8	Ideal structure	CShM =	5.52475		
Th	M	5.8018	4.7887	7.8758	
Cl	L3	6.2348	3.9150	6.0943	
Cl	L7	4.1498	3.6646	8.2393	
O	L6	6.6531	6.8840	6.2401	
O	L4	5.0856	6.4677	8.7661	
O	L1	5.8959	4.2431	10.6114	
O	L8	7.7369	5.1075	8.4036	
O	L5	3.8707	5.7647	6.1127	
O	L2	6.7874	2.2630	8.5389	

TT-8	Ideal structure	CShM =	11.29299		
Th	M	5.8018	4.7888	7.8758	
Cl	L6	6.1098	4.3538	5.3466	
Cl	L7	4.1644	3.0452	8.8555	
O	L3	7.1465	6.2208	7.0712	
O	L5	4.8477	7.1643	8.2329	
O	L2	5.5488	5.1460	9.9531	
O	L8	8.0865	4.5884	9.0678	
O	L4	3.9253	4.9534	6.8969	
O	L1	6.5853	2.8377	7.5826	

ETBPY-8	Ideal structure	CShM =	25.63769		
Th	M	5.8018	4.7887	7.8758	
Cl	L1	6.6792	4.0451	6.0410	
Cl	L8	4.6708	3.7746	9.4190	
O	L7	6.9328	5.8028	6.3326	
O	L4	3.7898	5.1169	8.6061	
O	L6	6.1959	4.5401	9.9905	
O	L5	7.6768	5.8679	7.9700	
O	L3	5.2706	6.4447	6.5856	
O	L2	5.1984	2.7173	8.0616	

Table S3: Crystal data and structure refinement for **4-ThCl$_2$(bipy)Mo$_5$**

Empirical formula	C31 H58 Cl4 Mo5 N4 O18 Th	
Formula weight	1628.35	
Temperature	100.00(10) K	
Wavelength	1.54184 Å	
Crystal system	triclinic	
Space group	*P*-1	
Unit cell dimensions	*a* = 12.0750(3) Å	α = 77.123(2)°
	b = 13.0320(4) Å	β = 73.305(2)°
	c = 18.5649(5) Å	γ = 63.453(3)°
Volume	2487.97(14) Å^3	
Z	2	
Density (calculated)	2.174 Mg/m^3	
Absorption coefficient	22.102 mm^{-1}	
F(000)	1568	
Crystal color, morphology	light violet, plate	
Crystal size	0.123 x 0.061 x 0.015 mm^3	
Theta range for data collection	2.500 to 80.218°	
Index ranges	-15 ≤ *h* ≤ 15, -16 ≤ *k* ≤ 15, -23 ≤ *l* ≤ 23	
Reflections collected	51921	
Independent reflections	10491 [*R*(int) = 0.0523]	
Observed reflections	9633	
Completeness to theta = 74.504°	98.9%	
Absorption correction	Multi-scan	
Max. and min. transmission	1.00000 and 0.34823	
Refinement method	Full-matrix least-squares on F^2	
Data / restraints / parameters	10491 / 0 / 576	
Goodness-of-fit on F^2	1.023	
Final *R* indices [*I*>2sigma(*I*)]	*R*1 = 0.0335, *wR*2 = 0.0803	
R indices (all data)	*R*1 = 0.0371, *wR*2 = 0.0818	
Largest diff. peak and hole	1.906 and -1.414 e.Å^{-3}	

Table S4: Continuous Shape Measures (CShM) analysis for **4-ThCl$_2$(bipy)Mo$_5$**. Positions in Cartesian coordinates (x,y,z).

Structure	**4-ThCl$_2$(bipy)Mo$_5$**		
Th	14.3039	7.9950	6.3872
Cl	13.2521	8.8556	8.7477
Cl	16.0513	10.0383	6.9784
O	14.3975	6.0216	4.9310
O	12.1126	7.0741	6.1382
O	12.9450	9.6945	5.4524
O	15.2716	8.5732	4.2453
N	14.5012	5.9310	8.0452
N	16.6225	6.8357	6.6896

OP-8	Ideal structure	CShM =	31.20890	
Th	M	14.3842	7.8910	6.4017
Cl	L1	12.7109	8.7615	7.3531
Cl	L3	15.0899	9.8136	5.8836
O	L6	15.0684	5.9159	6.0952
O	L8	12.7020	7.1470	7.4408
O	L2	13.7000	9.8661	6.7082
O	L4	16.0664	8.6350	5.3626
N	L7	13.6785	5.9684	6.9197
N	L5	16.0575	7.0205	5.4502

HPY-8	Ideal structure	CShM =	22.29486	
Th	M	14.2395	7.6963	6.3361
Cl	L2	13.1977	7.7959	8.3404
Cl	L1	15.5415	9.4483	6.9259
O	L5	15.8407	6.9880	4.9055
O	L8	12.3961	8.8731	6.9098
O	L7	12.9826	9.0642	5.0472
O	L6	14.5156	8.2253	4.1552
N	L3	14.7839	6.6437	8.2617
N	L4	15.9601	6.2841	6.7331

HBPY-8	Ideal structure	CShM =	15.64385	
Th	M	14.3842	7.8910	6.4017
Cl	L2	13.2306	8.9060	8.1657
Cl	L1	16.2295	9.2679	6.8161
O	L5	15.5378	6.8760	4.6376
O	L8	12.5389	6.5141	5.9872
O	L3	13.0639	9.7805	6.0023
O	L4	14.2176	8.7655	4.2383
N	L7	14.5508	7.0165	8.5650
N	L6	15.7044	6.0015	6.8010

CU-8	Ideal structure	CShM =	9.47164	
Th	M	14.3842	7.8910	6.4017
Cl	L1	13.8706	9.3432	8.2726
Cl	L2	16.2865	9.3205	6.8606
O	L7	14.8978	6.4388	4.5307
O	L8	12.4818	6.4615	5.9427
O	L4	12.4586	9.2585	5.8581
O	L3	14.8746	9.2358	4.4461
N	L5	13.8938	6.5462	8.3572

N	L6	16.3097	6.5235	6.9452

SAPR-8 Ideal structure CShM = 0.99066

Th	M	14.3842	7.8910	6.4017
Cl	L1	13.3192	8.8356	8.4986
Cl	L2	15.7801	9.9211	6.9962
O	L8	14.4233	5.8070	4.9599
O	L5	11.9847	7.0766	6.3501
O	L6	13.0263	9.8814	5.6156
O	L7	15.4649	8.6118	4.2254
N	L4	14.3071	5.9545	8.0349
N	L3	16.7679	7.0401	6.5326

TDD-8 Ideal structure CShM = 2.97021

Th	M	14.3842	7.8910	6.4017
Cl	L2	13.7810	8.8688	8.6321
Cl	L1	15.8474	9.9063	6.7063
O	L8	14.7635	5.8221	5.0338
O	L7	12.1104	6.9969	5.8313
O	L3	12.9403	9.8876	5.9284
O	L4	14.9959	8.6723	4.0973
N	L6	13.9855	5.9479	7.9382
N	L5	16.6495	7.0260	7.0460

JGBF-8 Ideal structure CShM = 16.79341

Th	M	14.3842	7.8910	6.4017
Cl	L3	13.8792	9.0800	7.7909
Cl	L1	16.2480	9.7891	6.7499
O	L5	14.8892	6.7020	5.0124
O	L7	12.0154	7.1819	7.4427
O	L6	12.9595	8.5386	5.3295
O	L2	15.3282	9.2477	4.2885
N	L8	13.9451	5.3453	7.1256
N	L4	15.8089	7.2434	7.4738

JETBPY-8 Ideal structure CShM = 29.37180

Th	M	14.3842	7.8910	6.4017
Cl	L1	13.4884	8.5209	7.7693
Cl	L3	14.4207	9.5873	5.9649
O	L6	14.4314	6.3727	5.5287
O	L5	12.8072	7.9885	5.6445
O	L7	12.2460	10.0182	6.5542
O	L4	16.0448	7.9715	5.8491
N	L2	15.1126	6.9051	7.6534
N	L8	16.5224	5.7638	6.2492

JBTPR-8 Ideal structure CShM = 3.68814

Th	M	14.2138	7.8792	6.1106
Cl	L1	13.8909	8.5643	8.3136
Cl	L7	15.8666	10.3312	6.8043
O	L6	14.4676	5.8341	5.0242
O	L5	12.0435	7.0810	6.3926
O	L3	13.0709	9.8626	5.6782
O	L4	15.4950	8.6157	4.3098
N	L8	14.0945	5.5336	8.0365
N	L2	16.3150	7.3174	6.9452

BTPR-8 Ideal structure CShM = 2.46081

Th	M	14.5278	7.9005	6.6436
Cl	L3	13.1645	8.9053	8.5275
Cl	L5	15.8162	10.0655	6.9078
O	L2	14.0767	6.2833	4.7466
O	L7	12.1277	7.0904	6.6263
O	L1	13.0250	9.3885	5.2491
O	L8	15.6355	8.6252	4.4837
N	L4	14.2163	5.8001	8.0251
N	L6	16.8680	6.9603	6.4054

JSD-8 Ideal structure CShM = 6.00434

Th	M	14.3842	7.8910	6.4017
Cl	L3	13.8846	8.6045	8.2627
Cl	L5	15.8618	10.2812	6.6806
O	L1	14.7638	5.4431	5.0460
O	L8	12.4895	7.2519	5.9286
O	L6	12.9264	10.2573	5.9019
O	L4	14.8889	8.4248	4.4827
N	L2	13.9848	5.5824	7.9781
N	L7	16.2737	7.2828	6.9326

TT-8 Ideal structure CShM = 10.30780

Th	M	14.3842	7.8910	6.4017
Cl	L2	13.9263	9.1886	8.0734
Cl	L7	16.4511	9.4519	6.8927
O	L6	14.9416	6.3109	4.3664
O	L3	12.6866	6.6089	5.9985
O	L8	12.2853	9.3734	5.8126
O	L1	14.8152	9.0912	4.6519
N	L5	13.8594	6.4296	8.5322
N	L4	16.1080	6.6734	6.8855

Table S5: Crystal data and structure refinement for **5-CalixThMo$_5$**

Empirical formula	C67 H108 Cl2 Mo5 N2 O22 Th	
Formula weight	2076.19	
Temperature	100.00(10) K	
Wavelength	1.54184 Å	
Crystal system	monoclinic	
Space group	*C*2	
Unit cell dimensions	*a* = 31.5317(3) Å	α = 90°
	b = 13.43370(10) Å	β = 128.550(2)°
	c = 27.1437(3) Å	γ = 90°
Volume	8992.0(2) Å^3	
Z	4	
Density (calculated)	1.534 Mg/m^3	
Absorption coefficient	11.860 mm^{-1}	
F(000)	4136	
Crystal color, morphology	colourless, needle	
Crystal size	0.559 x 0.061 x 0.042 mm^3	
Theta range for data collection	2.807 to 80.352°	
Index ranges	$-40 \leq h \leq 40$, $-16 \leq k \leq 12$, $-34 \leq l \leq 33$	
Reflections collected	78790	
Independent reflections	15622 [*R*(int) = 0.0531]	
Observed reflections	15270	
Completeness to theta = 74.504°	99.7%	
Absorption correction	Multi-scan	
Max. and min. transmission	1.000 and 0.479	
Refinement method	Full-matrix least-squares on F^2	
Data / restraints / parameters	15622 / 709 / 1168	
Goodness-of-fit on F^2	1.068	
Final *R* indices [*I*>2sigma(*I*)]	*R*1 = 0.0306, *wR*2 = 0.0814	
R indices (all data)	*R*1 = 0.0314, *wR*2 = 0.0819	
Absolute structure parameter	0.176(4)	
Largest diff. peak and hole	1.702 and -1.475 e.Å^{-3}	

Table S6: Continuous Shape Measures (CShM) analysis for **5-CalixThMo$_5$**. Positions in Cartesian coordinates (x,y,z).

Structure	**5-CalixThMo$_5$**		
Th	22.9633	-6.1976	-5.6063
O	23.4289	-3.9226	-4.7296
O	23.6344	-6.3582	-3.2267
O	24.9664	-7.5968	-5.3771
O	24.7616	-5.1532	-6.8928
O	20.9643	-5.3533	-4.1692
O	21.6951	-7.9675	-5.4068
O	23.3227	-7.4275	-7.8629
O	21.6824	-5.1693	-6.9841

OP-8	Ideal structure	CShM =	31.08812	
Th	M	23.0466	-6.1273	-5.5839
O	L1	22.3043	-4.2903	-5.2243
O	L3	22.4215	-6.7353	-3.7688
O	L5	23.7888	-7.9644	-5.9436
O	L7	23.6717	-5.5194	-7.3991
O	L2	22.0797	-5.2582	-4.0461
O	L4	23.1294	-7.8562	-4.5548
O	L6	24.0134	-6.9965	-7.1218
O	L8	22.9637	-4.3984	-6.6131

HPY-8	Ideal structure	CShM =	23.74116	
Th	M	23.1579	-5.9231	-5.6294
O	L2	22.5072	-5.1503	-3.7506
O	L3	24.1339	-6.0337	-3.7357
O	L4	25.0256	-6.8338	-5.1469
O	L5	24.5110	-6.9482	-6.9213
O	L8	21.3705	-4.8488	-5.1802
O	L1	22.1559	-7.7613	-5.2203
O	L6	22.9774	-6.2906	-7.7230
O	L7	21.5798	-5.3563	-6.9481

HBPY-8	Ideal structure	CShM =	16.80656	
Th	M	23.0466	-6.1273	-5.5839
O	L1	23.8032	-4.0558	-5.4052
O	L2	23.1614	-6.3589	-3.3865
O	L3	24.9019	-6.8858	-4.6469
O	L4	24.7870	-6.6543	-6.8443
O	L7	21.3061	-5.6004	-4.3235
O	L8	22.2900	-8.1988	-5.7627
O	L5	22.9317	-5.8958	-7.7814
O	L6	21.1912	-5.3689	-6.5210

CU-8	Ideal structure	CShM =	12.05689	
Th	M	23.0466	-6.1273	-5.5839
O	L1	23.7969	-3.9879	-5.3976
O	L2	23.4087	-5.6389	-3.3918
O	L3	24.6032	-7.5534	-4.7364
O	L4	24.9915	-5.9023	-6.7422
O	L6	21.1016	-6.3523	-4.4257
O	L7	22.2962	-8.2668	-5.7703
O	L8	22.6845	-6.6157	-7.7761

O	L5	21.4899	-4.7013	-6.4315

SAPR-8 Ideal structure CShM = 2.23094

Th	M	23.0466	-6.1273	-5.5839
O	L1	23.3582	-3.9081	-4.7290
O	L2	23.4734	-6.4128	-3.2410
O	L3	24.8802	-7.6690	-5.4644
O	L4	24.7650	-5.1643	-6.9524
O	L6	20.9792	-5.5779	-4.4990
O	L7	22.0554	-8.2372	-5.0190
O	L8	22.9687	-7.3544	-7.6434
O	L5	21.8925	-4.6951	-7.1234

TDD-8 Ideal structure CShM = 2.08185

Th	M	23.0466	-6.1273	-5.5839
O	L1	23.2030	-3.8087	-4.9824
O	L2	23.8151	-6.0713	-3.3106
O	L6	24.6720	-7.8817	-5.3784
O	L5	25.0603	-5.3025	-6.5971
O	L3	21.1206	-5.6503	-4.2330
O	L7	21.8087	-8.1779	-5.4269
O	L8	23.1907	-7.1685	-7.7420
O	L4	21.5022	-4.9577	-7.0012

JGBF-8 Ideal structure CShM = 13.60412

Th	M	23.0466	-6.1273	-5.5839
O	L3	24.2165	-4.7751	-5.1458
O	L1	23.3020	-5.9322	-3.0003
O	L4	24.2480	-7.3713	-4.9529
O	L7	23.9611	-4.9702	-7.7295
O	L2	20.9306	-6.0404	-4.0695
O	L5	21.8766	-7.4796	-6.0221
O	L8	23.9926	-7.5665	-7.5365
O	L6	21.8451	-4.8833	-6.2150

JETBPY-8 Ideal structure CShM = 26.69281

Th	M	23.0466	-6.1273	-5.5839
O	L1	23.0852	-4.4603	-5.2535
O	L3	23.0704	-6.2167	-3.8865
O	L4	24.1361	-7.4224	-5.4242
O	L2	24.1509	-5.6660	-6.7912
O	L7	21.6436	-4.5400	-3.5596
O	L6	22.4513	-7.1021	-6.8430
O	L8	24.4495	-7.7146	-7.6083
O	L5	21.3856	-5.8964	-5.3053

JBTPR-8 Ideal structure CShM = 0.97508

Th	M	23.3317	-6.0364	-5.4549
O	L3	23.3027	-3.9007	-4.7484
O	L4	23.4267	-6.4287	-3.2417
O	L6	24.8483	-7.6977	-5.4877
O	L5	24.7244	-5.1697	-6.9944
O	L7	20.8227	-5.3512	-4.0988
O	L2	21.9059	-7.7748	-5.3751
O	L8	23.2747	-7.5399	-7.9727
O	L1	21.7820	-5.2469	-6.8818

BTPR-8 Ideal structure CShM = 1.44207

Th	M	23.2824	-6.0520	-5.4775
O	L3	23.2511	-3.7493	-4.7173
O	L4	23.3869	-6.4733	-3.0915
O	L6	24.9178	-7.8424	-5.5131
O	L5	24.7820	-5.1183	-7.1390
O	L7	21.2087	-5.4854	-4.3549
O	L2	21.7461	-7.9264	-5.3890
O	L8	23.2339	-7.2965	-7.5584
O	L1	21.6103	-5.2024	-7.0149

JSD-8 Ideal structure CShM = 3.12750

Th	M	23.0466	-6.1273	-5.5839
O	L1	23.0416	-3.5124	-4.7987
O	L7	23.7386	-6.1538	-3.7219
O	L5	24.8660	-8.1626	-5.5429
O	L4	24.6937	-5.3536	-6.3808
O	L2	20.9198	-5.3939	-4.0369
O	L3	21.9584	-7.7791	-5.3987
O	L6	23.3588	-7.4404	-7.9572
O	L8	21.7956	-5.2228	-6.8344

TT-8 Ideal structure CShM = 12.25497

Th	M	23.0466	-6.1273	-5.5840
O	L6	23.8666	-3.7916	-5.3871
O	L1	23.3667	-5.6815	-3.6197
O	L7	24.7426	-7.6807	-4.6472
O	L4	24.7924	-5.9312	-6.6200
O	L8	20.9208	-6.3662	-4.3226
O	L2	22.3730	-8.0457	-5.7458
O	L5	22.6567	-6.6702	-7.9757
O	L3	21.6536	-4.8516	-6.3534

Table S7: Crystal data and structure refinement for **6-ThCl(DMF)$_4$Mo$_5$**

Empirical formula	C16 H40 Cl Mo5 N5 O22 Th	
Formula weight	1401.72	
Temperature	100.00(10) K	
Wavelength	1.54184 Å	
Crystal system	monoclinic	
Space group	$P2_1/c$	
Unit cell dimensions	*a* = 18.82790(10) Å	α = 90°
	b = 21.73200(10) Å	β = 108.3680(10)°
	c = 19.71710(10) Å	γ = 90°
Volume	7656.58(8) Å^3	
Z	8	
Density (calculated)	2.432 Mg/m^3	
Absorption coefficient	26.775 mm^{-1}	
F(000)	5312	
Crystal color, morphology	violet, needle	
Crystal size	0.216 x 0.066 x 0.064 mm^3	
Theta range for data collection	3.116 to 80.241°	
Index ranges	$-23 \le h \le 24$, $-27 \le k \le 25$, $-25 \le l \le 23$	
Reflections collected	128098	
Independent reflections	16526 [*R*(int) = 0.0552]	
Observed reflections	16122	
Completeness to theta = 74.504°	100.0%	
Absorption correction	Multi-scan	
Max. and min. transmission	1.00000 and 0.08574	
Refinement method	Full-matrix least-squares on F^2	
Data / restraints / parameters	16526 / 0 / 925	
Goodness-of-fit on F^2	1.114	
Final *R* indices [*I*>2sigma(*I*)]	*R*1 = 0.0325, *wR*2 = 0.0846	
R indices (all data)	*R*1 = 0.0335, *wR*2 = 0.0852	
Largest diff. peak and hole	2.561 and -1.815 e.Å^{-3}	

Table S8: Continuous Shape Measures (CShM) analysis for the two unique complexes in the structure of **6-ThCl(DMF)$_4$Mo$_5$**. Positions in Cartesian coordinates (x,y,z).

Structure	**6-ThCl(DMF)$_4$Mo$_5$** (1)		
Th	3.3356	8.3025	7.7032
Cl	3.4840	10.7032	6.3035
O	1.4110	6.8143	8.2709
O	3.6827	7.1183	9.8417
O	3.4532	9.8600	9.6269
O	1.1958	9.5327	8.0610
O	4.0282	5.9691	7.3971
O	5.5946	8.6689	8.2990
O	4.8437	7.9513	5.7747
O	1.9887	7.8174	5.7485

EP-9	Ideal structure	CShM =	37.22247	
Th	M	3.3017	8.2738	7.7027
Cl	L1	3.4208	9.0662	5.8956
O	L4	1.5974	8.3424	8.7017
O	L6	3.8395	7.3455	9.3630
O	L5	2.5403	7.7127	9.4384
O	L3	1.4520	8.9400	7.4975
O	L7	4.8871	7.4127	8.5107
O	L8	5.1928	7.8828	7.2803
O	L9	4.6138	8.5358	6.2475
O	L2	2.1721	9.2259	6.3893

OPY-9	Ideal structure	CShM =	22.58852	
Th	M	3.1714	8.2927	7.8798
Cl	L2	2.9891	10.4619	7.5140
O	L5	2.0471	6.5026	7.2441
O	L7	4.5537	7.0150	9.0329
O	L9	4.2958	10.0828	8.5155
O	L3	1.7892	9.5704	6.7268
O	L6	3.3538	6.1235	8.2456
O	L8	4.9439	8.6550	9.1447
O	L1	4.4745	8.1035	6.1081
O	L4	1.3990	7.9304	6.6150

HBPY-9	Ideal structure	CShM =	18.70239	
Th	M	3.3017	8.2738	7.7027
Cl	L1	3.1110	9.8178	6.0778
O	L2	1.0672	8.0154	7.7195
O	L9	3.4925	6.7298	9.3275
O	L4	3.6283	9.9062	9.2155
O	L3	1.7717	9.3757	8.9294
O	L7	3.9696	6.7563	6.1823
O	L5	5.2391	9.2075	8.3624
O	L6	5.3910	7.8056	7.0125
O	L8	2.0454	6.8497	6.4969

JTC-9	Ideal structure	CShM =	16.34975	
Th	M	3.5928	8.7430	7.8754
Cl	L1	2.7870	9.9330	6.0010
O	L9	1.5398	7.5845	8.0226
O	L4	4.3987	7.5530	9.7497

O	L5	2.3716	8.7563	9.8971
O	L6	1.5658	9.9463	8.0227
O	L8	3.5669	6.3812	7.8753
O	L3	5.6199	7.5396	7.7281
O	L2	4.8140	8.7297	5.8537
O	L7	2.7610	7.5712	6.0009

JCCU-9 Ideal structure CShM = 9.44534

Th	M	3.1148	8.2939	7.9591
Cl	L1	3.2011	9.8231	6.2685
O	L4	1.0001	7.4532	8.1166
O	L6	3.0285	6.7647	9.6496
O	L8	3.6809	9.3018	9.9257
O	L2	1.6525	9.9902	8.3927
O	L5	4.5771	6.5977	7.5254
O	L7	5.2295	9.1347	7.8015
O	L9	4.9842	8.0923	5.3949
O	L3	2.5487	7.2861	5.9924

CCU-9 Ideal structure CShM = 7.89358

Th	M	3.2894	8.2751	7.7195
Cl	L1	3.1264	10.1253	6.2083
O	L6	1.0444	7.5179	8.0661
O	L8	3.0101	6.7674	9.5586
O	L7	3.7105	9.2300	9.8746
O	L5	1.7448	9.9806	8.3821
O	L4	4.6340	6.2968	7.6102
O	L3	5.4685	9.2310	7.9866
O	L9	4.6973	8.1224	5.7886
O	L2	2.2919	7.1911	5.8318

JCSAPR-9 Ideal structure CShM = 1.83200

Th	M	3.1118	8.2960	7.9673
Cl	L2	3.6276	10.2284	6.6732
O	L5	1.3699	6.7171	8.3517
O	L7	3.7095	7.1905	9.9910
O	L1	3.4373	10.0404	9.5566
O	L3	1.0977	9.5671	7.9173
O	L6	4.0125	6.1979	7.2877
O	L8	5.4744	8.5479	8.1396
O	L9	5.0111	8.0739	5.3208
O	L4	2.1657	7.8785	5.8213

CSAPR-9 Ideal structure CShM = 0.55840

Th	M	3.3004	8.2739	7.7046
Cl	L2	3.5985	10.4938	6.6216
O	L7	1.4276	6.7723	8.3584
O	L8	3.6814	7.2384	9.9344
O	L9	3.4107	9.9821	9.5101
O	L6	1.1569	9.5159	7.9340
O	L4	4.0486	5.9314	7.3273
O	L5	5.6975	8.6002	8.2849
O	L1	4.7462	8.1045	5.6870
O	L3	1.9496	7.8251	5.6641

JTCTPR-9 Ideal structure CShM = 3.53770

Th	M	3.3017	8.2738	7.7026
Cl	L1	3.5980	10.2270	6.7200
O	L8	1.2061	6.3497	8.1294
O	L4	3.2456	7.5502	9.7863
O	L9	3.7159	10.3946	9.6017
O	L2	1.4080	9.2256	8.3160
O	L6	3.9805	6.2064	7.3368
O	L3	5.4356	8.5516	8.1904
O	L7	4.9833	8.0770	5.3768
O	L5	2.1428	7.8818	5.8664

TCTPR-9 Ideal structure CShM = 1.54674

Th	M	3.3017	8.2738	7.7027
Cl	L1	3.3295	10.4474	6.5184
O	L7	1.4986	6.6183	8.0717
O	L5	3.5126	7.3169	9.9760
O	L8	3.6596	10.0990	9.3362
O	L2	1.1638	9.4570	8.0994
O	L6	4.2375	5.9869	7.5519
O	L4	5.6783	8.3073	8.3949
O	L9	4.7470	8.1040	5.7000
O	L3	1.8888	8.1270	5.6753

JTDIC-9 Ideal structure CShM = 12.73988

Th	M	3.2179	8.3504	7.6366
Cl	L5	3.0005	9.6109	5.6835
O	L1	2.0073	6.3564	7.7325
O	L2	3.4353	7.0899	9.5897
O	L7	3.4231	9.5443	9.6324
O	L9	1.9876	10.3277	7.8016
O	L8	4.4482	6.3730	7.4716
O	L3	5.3231	8.3433	8.6458
O	L4	5.0620	8.3844	6.2053
O	L6	1.1126	8.3575	6.6274

HH-9 Ideal structure CShM = 11.47900

Th	M	3.2750	8.4199	7.6152
Cl	L7	2.9098	10.4157	6.4210
O	L2	1.0584	7.7551	7.1820
O	L9	3.8404	7.3154	9.6161
O	L4	3.7732	9.6678	9.5485
O	L3	1.5566	9.0030	9.1152
O	L8	3.3422	6.0675	7.6828
O	L5	5.4916	9.0847	8.0485
O	L6	4.9934	7.8368	6.1152
O	L1	2.7768	7.1720	5.6820

MFF-9 Ideal structure CShM = 0.71964

Th	M	3.3160	8.3704	7.6532
Cl	L9	3.6302	10.5719	6.5398
O	L6	1.4342	6.8513	8.2476
O	L7	3.7700	7.3408	9.8748
O	L3	3.3914	9.8240	9.6713
O	L4	1.0556	9.3345	8.0441
O	L8	4.0393	6.0125	7.3242

O	L2	5.6914	8.7853	8.2666
O	L1	4.7773	7.6538	5.7711
O	L5	1.9121	7.9933	5.6337

Structure **6-ThCl(DMF)$_4$Mo$_5$** (2)

Th	-0.1392	19.1209	10.7133
Cl	-0.4867	21.8483	11.4027
O	1.8319	17.6038	10.7281
O	-0.1881	17.4845	8.8583
O	0.0252	20.2018	8.5034
O	2.0215	20.3029	10.3958
O	-0.7967	16.9249	11.5176
O	-2.3227	19.2276	9.7343
O	-1.8882	19.1974	12.4345
O	0.9456	19.2626	12.8649

EP-9 Ideal structure CShM = 37.40139

Th	M	-0.0997	19.1175	10.7153
Cl	L1	0.4459	20.8624	11.4617
O	L4	0.9164	18.3216	9.2207
O	L5	-0.1034	17.5080	9.5710
O	L3	1.4607	19.5076	9.5697
O	L2	1.2749	20.5110	10.4547
O	L6	-1.1215	17.4475	10.4568
O	L7	-1.6615	18.1684	11.4635
O	L8	-1.4707	19.3333	12.1201
O	L9	-0.6384	20.3973	12.1194

OPY-9 Ideal structure CShM = 22.31762

Th	M	0.0539	19.0993	10.5572
Cl	L2	0.1630	21.3038	10.4097
O	L5	1.0996	17.5577	11.7503
O	L7	-1.1462	17.5232	9.5726
O	L9	-0.9919	20.6409	9.3640
O	L3	1.2540	20.6754	11.5417
O	L6	-0.0552	16.8948	10.7046
O	L8	-1.5342	19.0749	9.0173
O	L1	-1.4823	19.2811	12.1385
O	L4	1.6419	19.1237	12.0970

HBPY-9 Ideal structure CShM = 19.45030

Th	M	-0.0997	19.1175	10.7153
Cl	L2	0.5938	21.0749	11.5552
O	L1	1.8741	18.1949	11.2355
O	L5	-0.3772	17.6052	9.0862
O	L4	0.5264	19.2465	8.5684
O	L3	0.9585	20.7906	9.6672
O	L6	-1.0719	17.1027	10.8308
O	L9	-2.0736	20.0400	10.1951
O	L7	-1.0345	18.1174	12.4884
O	L8	-0.2932	19.8851	12.8108

JTC-9	Ideal structure	CShM =	16.62179	
Th	M	-0.3671	19.5169	10.3945
Cl	L1	0.1573	21.1602	12.0037
O	L9	1.6930	18.4473	10.8155
O	L4	-0.8915	17.8735	8.7853
O	L5	1.1240	19.0879	8.6175
O	L6	1.6484	20.7312	10.2267
O	L8	-0.3225	17.2329	10.9833
O	L3	-2.3826	18.3025	10.5623
O	L2	-1.8582	19.9459	12.1715
O	L7	0.2018	18.8763	12.5925

JCCU-9	Ideal structure	CShM =	10.53818	
Th	M	0.1188	19.0827	10.4894
Cl	L1	-0.2070	20.9613	11.7182
O	L4	2.2306	18.3222	10.8163
O	L6	0.4445	17.2041	9.2607
O	L8	-0.1830	19.5550	8.2915
O	L2	1.6030	20.6731	9.8471
O	L5	-1.3655	17.4922	11.1317
O	L7	-1.9931	19.8432	10.1625
O	L9	-2.0662	19.4305	12.7480
O	L3	0.4205	18.6103	12.6874

CCU-9	Ideal structure	CShM =	8.91418	
Th	M	-0.0854	19.1152	10.7004
Cl	L1	-0.1467	21.2726	11.7086
O	L6	2.1818	18.3981	10.8427
O	L8	0.4525	17.2272	9.3510
O	L7	-0.2206	19.4935	8.3524
O	L5	1.5087	20.6644	9.8441
O	L4	-1.4051	17.1772	11.1211
O	L3	-2.2071	19.8775	9.9313
O	L9	-1.7309	19.3766	12.4029
O	L2	0.6553	18.5723	12.8984

JCSAPR-9	Ideal structure	CShM =	1.88721	
Th	M	0.1245	19.0827	10.4805
Cl	L2	-0.5762	21.2485	11.1836
O	L5	1.9131	17.5280	10.7258
O	L7	-0.1833	17.4983	8.7280
O	L1	0.0083	20.3777	8.4841
O	L3	2.1047	20.4074	10.4819
O	L6	-0.8472	17.1764	11.5285
O	L8	-2.1941	19.1914	9.9434
O	L9	-2.1180	19.4308	12.8286
O	L4	0.7707	19.2335	12.7687

CSAPR-9	Ideal structure	CShM =	0.50355	
Th	M	-0.0981	19.1172	10.7136
Cl	L2	-0.5591	21.5210	11.1692
O	L7	1.8594	17.5790	10.6995
O	L8	-0.1626	17.5560	8.7754
O	L9	0.0263	20.3313	8.5436
O	L6	2.0484	20.3543	10.4677
O	L4	-0.8733	16.9059	11.5547

O	L5	-2.3974	19.1943	9.7621
O	L1	-1.8061	19.3831	12.5053
O	L3	0.9651	19.2326	12.9618

JTCTPR-9 Ideal structure CShM = 3.52221

Th	M	-0.0997	19.1175	10.7153
Cl	L1	-0.5360	21.2382	11.1458
O	L4	1.7173	17.9336	10.3026
O	L8	-0.5079	17.1958	8.6119
O	L2	0.3715	19.9468	8.7245
O	L9	2.2818	20.7334	10.7460
O	L6	-0.8077	17.1740	11.4866
O	L5	-2.1534	19.1871	9.9084
O	L7	-2.0731	19.4232	12.7880
O	L3	0.8098	19.2251	12.7239

TCTPR-9 Ideal structure CShM = 1.50433

Th	M	-0.0997	19.1175	10.7153
Cl	L1	-0.3059	21.5119	11.3154
O	L7	1.7510	17.4964	11.0029
O	L5	0.0091	17.6293	8.7381
O	L8	-0.2458	20.4768	8.6497
O	L2	2.0643	20.2566	10.3218
O	L6	-1.0300	16.8976	11.3005
O	L4	-2.3612	18.8845	9.7317
O	L9	-1.8044	19.3792	12.4933
O	L3	1.0252	19.5249	12.8842

JTDIC-9 Ideal structure CShM = 13.09135

Th	M	-0.0266	19.2111	10.7709
Cl	L5	-0.0912	20.9087	12.3668
O	L1	1.2061	17.3106	11.3199
O	L2	0.0380	17.5135	9.1749
O	L7	0.0282	19.8804	8.5388
O	L9	1.1903	21.1402	10.2906
O	L8	-1.2436	17.2820	11.2511
O	L3	-1.9716	18.8702	9.5323
O	L4	-2.0454	19.5058	11.8982
O	L6	1.9184	19.5520	12.0094

HH-9 Ideal structure CShM = 12.13998

Th	M	-0.0866	19.2811	10.7660
Cl	L7	0.0933	21.5158	11.4584
O	L2	2.0477	18.8398	11.6357
O	L9	-0.3608	17.6998	9.0541
O	L4	-0.3236	19.9894	8.5415
O	L3	1.8106	19.5481	9.4112
O	L8	-0.1238	16.9915	11.2785
O	L5	-2.2208	19.7223	9.8963
O	L6	-1.9838	19.0140	12.1207
O	L1	0.1505	18.5728	12.9904

MFF-9 Ideal structure CShM = 0.66386

Th	M	-0.1214	19.2226	10.7371
Cl	L9	-0.6029	21.6156	11.2224
O	L6	1.8372	17.6825	10.7878

O	L7	-0.2582	17.6647	8.7969
O	L3	0.0699	20.1380	8.4294
O	L4	2.1653	20.1558	10.4203
O	L8	-0.8653	16.9862	11.5401
O	L2	-2.3948	19.3775	9.7335
O	L1	-1.8228	18.9254	12.5305
O	L5	0.9955	19.4064	12.9549

Table S9: Crystal data and structure refinement for **7-TpTh(DMF)$_2$Mo$_5$**

Empirical formula	C22.03 H43.41 B Cl0.65 Mo5 N9 O20.67 Th	
Formula weight	1510.82	
Temperature	100.00(10) K	
Wavelength	1.54184 Å	
Crystal system	triclinic	
Space group	*P*-1	
Unit cell dimensions	*a* = 9.41160(10) Å	α = 71.2030(10)°
	b = 14.59830(10) Å	β = 82.9960(10)°
	c = 17.00110(10) Å	γ = 88.7880(10)°
Volume	2194.35(3) Å^3	
Z	2	
Density (calculated)	2.287 Mg/m^3	
Absorption coefficient	23.237 mm^{-1}	
F(000)	1440	
Crystal color, morphology	light violet, plate	
Crystal size	0.093 x 0.053 x 0.022 mm^3	
Theta range for data collection	2.766 to 80.233°	
Index ranges	$-12 \le h \le 11$, $-18 \le k \le 17$, $-21 \le l \le 21$	
Reflections collected	74849	
Independent reflections	9427 [*R*(int) = 0.0472]	
Observed reflections	9196	
Completeness to theta = 74.504°	99.8%	
Absorption correction	Multi-scan	
Max. and min. transmission	1.00000 and 0.71375	
Refinement method	Full-matrix least-squares on F^2	
Data / restraints / parameters	9427 / 83 / 631	
Goodness-of-fit on F^2	1.107	
Final *R* indices [*I*>2sigma(*I*)]	*R*1 = 0.0290, *wR*2 = 0.0800	
R indices (all data)	*R*1 = 0.0297, *wR*2 = 0.0805	
Extinction coefficient	0.000298(18)	
Largest diff. peak and hole	1.487 and -1.177 e.Å^{-3}	

Table S10: Continuous Shape Measures (CShM) analysis for **7-TpTh(DMF)$_2$Mo$_5$**. Positions in Cartesian coordinates (x,y,z).

Structure	**7-TpTh(DMF)$_2$Mo$_5$**		
Th	9.2852	11.4428	11.3560
O	7.0529	12.4108	11.7080
O	9.3391	13.1579	13.0930
O	10.7170	13.2822	10.7303
O	8.4049	12.4744	9.3213
O	10.6390	10.7995	9.4475
O	11.3818	11.1972	12.5594
N	7.4440	9.7259	10.6632
N	8.2933	10.3566	13.5291
N	10.0918	8.9197	11.5195

EP-9	Ideal structure	CShM =	36.02475	
Th	M	9.2649	11.3767	11.3927
O	L1	7.5491	12.0140	12.1997
O	L2	8.1749	13.0460	11.5551
O	L4	10.4251	12.6495	10.3752
O	L3	9.3107	13.2970	10.8346
O	L5	10.9966	11.4064	10.3920
O	L6	10.7579	10.1494	10.8770
N	L8	8.6232	9.6777	12.2311
N	L9	7.7262	10.6837	12.4666
N	L7	9.8205	9.4667	11.6033

OPY-9	Ideal structure	CShM =	22.14399	
Th	M	9.1990	11.5878	11.3743
O	L2	7.1542	11.0051	12.0092
O	L4	9.7547	11.9456	13.4927
O	L6	11.2439	12.1705	10.7395
O	L8	8.6434	11.2300	9.2560
O	L7	10.2521	11.7468	9.4276
O	L5	11.0379	12.2528	12.4233
N	L9	7.3602	10.9228	10.3254
N	L3	8.1460	11.4288	13.3211
N	L1	9.8576	9.4768	11.5582

HBPY-9	Ideal structure	CShM =	19.36787	
Th	M	9.2649	11.3767	11.3927
O	L2	7.6869	12.9288	11.0132
O	L3	8.0680	12.5510	12.8865
O	L1	10.8393	12.9780	11.3953
O	L8	8.4941	12.1378	9.4256
O	L7	9.8817	10.7737	9.3193
O	L5	10.5684	10.0930	12.6951
N	L9	7.6905	9.7754	11.3901
N	L4	9.3504	11.2890	13.6350
N	L6	10.8048	9.8637	10.7744

JTC-9	Ideal structure	CShM =	16.13124	
Th	M	9.1290	11.2798	10.8365
O	L1	6.8443	11.6821	11.3247
O	L9	9.7574	12.9321	12.4161
O	L5	10.5345	13.0728	10.1809

O	L6	8.2498	13.4751	10.6691
O	L4	11.4137	10.8776	10.3483
O	L8	10.6367	10.7369	12.5836
N	L2	7.7235	9.4868	11.4922
N	L7	8.3519	11.1391	13.0718
N	L3	10.0082	9.0846	11.0040

JCCU-9 Ideal structure CShM = 10.19179

Th	M	9.1776	11.6804	11.3628
O	L2	7.0532	12.4066	10.9818
O	L4	8.5202	13.0341	13.0719
O	L6	10.5792	13.4702	11.4957
O	L8	9.1123	12.8427	9.4057
O	L7	9.8351	10.3268	9.6537
O	L5	11.3021	10.9543	11.7438
N	L1	7.7761	9.8906	11.2298
N	L3	9.2430	10.5181	13.3199
N	L9	10.0502	8.6434	11.6622

CCU-9 Ideal structure CShM = 9.04215

Th	M	9.2592	11.3966	11.3908
O	L5	7.1213	12.3507	10.9341
O	L6	8.4861	12.9503	13.0272
O	L8	10.5450	13.3988	11.5563
O	L7	9.1801	12.7992	9.4631
O	L3	9.9486	10.3491	9.3618
O	L4	11.5748	11.0635	11.8558
N	L1	7.4955	9.8147	11.1144
N	L2	9.1217	10.5291	13.6084
N	L9	9.9168	9.1149	11.6155

JCSAPR-9 Ideal structure CShM = 1.34511

Th	M	9.1732	11.6901	11.3606
O	L1	6.9225	12.3600	11.8255
O	L3	9.3408	13.2088	13.2034
O	L5	10.7399	13.3573	10.6563
O	L7	8.3216	12.5085	9.2784
O	L6	10.5045	10.6265	9.6793
O	L4	11.2252	11.1218	12.4546
N	L8	7.8052	9.9213	10.5061
N	L2	8.5258	10.4165	13.2814
N	L9	10.0903	8.5563	11.6817

CSAPR-9 Ideal structure CShM = 0.40243

Th	M	9.2642	11.3790	11.3925
O	L6	7.0036	12.3516	11.8081
O	L7	9.3306	13.1703	13.1292
O	L8	10.6727	13.3115	10.6777
O	L9	8.3457	12.4929	9.3566
O	L5	10.6331	10.6339	9.4431
O	L4	11.4520	11.1972	12.5798
N	L2	7.5824	9.8359	10.3829
N	L3	8.4014	10.3991	13.5196
N	L1	9.9633	8.9957	11.6379

JTCTPR-9 Ideal structure CShM = 3.05522

Th	M	9.2649	11.3767	11.3927
O	L1	7.4480	12.6223	11.6450
O	L7	9.4819	13.1833	13.6391
O	L3	10.1380	13.3312	10.8150
O	L9	8.2340	12.3298	8.8657
O	L4	10.4841	10.6837	9.6753
O	L6	11.2076	11.1786	12.4429
N	L2	7.7941	9.9748	10.5053
N	L5	8.5176	10.4696	13.2729
N	L8	10.0788	8.6170	11.6734

TCTPR-9 Ideal structure CShM = 1.00512

Th	M	9.2649	11.3767	11.3927
O	L1	7.0787	12.5055	11.7637
O	L8	9.4523	12.9328	13.3253
O	L4	10.5138	13.4140	10.6991
O	L9	8.3745	12.1952	9.2182
O	L6	10.8575	10.7935	9.5720
O	L5	11.5761	11.2853	12.3101
N	L3	7.4224	9.8851	10.6366
N	L2	8.1409	10.3768	13.3747
N	L7	9.9679	9.0021	11.6347

JTDIC-9 Ideal structure CShM = 11.83782

Th	M	9.1425	11.3442	11.4314
O	L5	6.8720	11.6330	10.8884
O	L1	9.9206	12.4794	13.3391
O	L8	10.5566	13.1861	11.0557
O	L4	8.6724	12.6630	9.5411
O	L3	10.6416	11.1689	9.6271
O	L2	11.4130	11.0554	11.9744
N	L9	7.7285	9.5022	11.8070
N	L6	7.6434	11.5195	13.2356
N	L7	10.0582	9.2154	11.0275

HH-9 Ideal structure CShM = 12.32435

Th	M	9.2046	11.4343	11.2423
O	L2	7.2226	12.1206	12.2992
O	L3	9.0400	13.5996	12.1372
O	L4	11.0220	12.9132	11.0804
O	L7	8.3816	12.2207	9.1880
O	L5	11.1867	10.7479	10.1855
O	L9	10.8261	11.4927	12.9405
N	L1	7.3873	9.9553	11.4043
N	L8	9.0088	10.0137	13.1025
N	L6	9.3693	9.2690	10.3475

MFF-9 Ideal structure CShM = 0.62121

Th	M	9.2279	11.3316	11.4857
O	L3	7.0133	12.4754	11.6084
O	L4	9.4213	13.3226	12.9778
O	L6	10.6938	13.2325	10.7959
O	L7	8.2857	12.3852	9.4265
O	L8	10.5928	10.6357	9.5160
O	L5	11.3767	11.1980	12.7473

N	L2	7.4805	9.8271	10.5317
N	L9	8.3796	10.3213	13.6025
N	L1	10.1773	9.0376	11.2355
